# Mucin-Type *O*-Glycosylation Proximal to β-Secretase Cleavage Site Affects APP Processing and Aggregation Fate

**DOI:** 10.3389/fchem.2022.859822

**Published:** 2022-04-08

**Authors:** YashoNandini Singh, Deepika Regmi, David Ormaza, Ramya Ayyalasomayajula, Nancy Vela, Gustavo Mundim, Deguo Du, Dmitriy Minond, Maré Cudic

**Affiliations:** ^1^ Department of Chemistry and Biochemistry, Charles E. Schmidt College of Science, Florida Atlantic University, Boca Raton, FL, United States; ^2^ College of Pharmacy and Rumbaugh-Goodwin Institute for Cancer Research, Nova Southeastern University, Fort Lauderdale, FL, United States

**Keywords:** Alzheimer’s disease, circular dichroism, proteolysis, aggregation kinetics, APP *O*-glycopeptides

## Abstract

The amyloid-β precursor protein (APP) undergoes proteolysis by β- and γ-secretases to form amyloid-β peptides (Aβ), which is a hallmark of Alzheimer’s disease (AD). Recent findings suggest a possible role of *O*-glycosylation on APP’s proteolytic processing and subsequent fate for AD-related pathology. We have previously reported that Tyr^681^-*O*-glycosylation and the Swedish mutation accelerate cleavage of APP model glycopeptides by β-secretase (amyloidogenic pathway) more than α-secretase (non-amyloidogenic pathway). Therefore, to further our studies, we have synthesized additional native and Swedish-mutated (glyco)peptides with *O*-GalNAc moiety on Thr^663^ and/or Ser^667^ to explore the role of glycosylation on conformation, secretase activity, and aggregation kinetics of Aβ40. Our results show that conformation is strongly dependent on external conditions such as buffer ions and solvent polarity as well as internal modifications of (glyco)peptides such as length, *O*-glycosylation, and Swedish mutation. Furthermore, the level of β-secretase activity significantly increases for the glycopeptides containing the Swedish mutation compared to their nonglycosylated and native counterparts. Lastly, the glycopeptides impact the kinetics of Aβ40 aggregation by significantly increasing the lag phase and delaying aggregation onset, however, this effect is less pronounced for its Swedish-mutated counterparts. In conclusion, our results confirm that the Swedish mutation and/or *O*-glycosylation can render APP model glycopeptides more susceptible to cleavage by β-secretase. In addition, this study sheds new light on the possible role of glycosylation and/or glycan density on the rate of Aβ40 aggregation.

## Introduction

Alzheimer disease (AD) is one of the most common neurodegenerative disorders linked to aging (van Cauwenberghe et al., 2016; Alzheimer’s Association, 2020). It has a profound effect on the economy, health-care system, and the society, and is projected to increase even further as the population ages (Hurd et al., 2013). Genetic, biochemical, and behavioral research suggest that physiologic generation of the Aβ-forming fibrils stems from the proteolytic processing of the amyloid precursor protein (APP), a type 1 transmembrane glycoprotein, by β-secretase (BACE-1) (Hardy and Higgins, 1992; O’Brien and Wong, 2011; Selkoe and Hardy, 2016). This pathway co-exists with the nonamyloidogenic pathway, that is, initiated by α-secretase within the Aβ domain and precludes Aβ formation.

Despite the ongoing debates about the validity of amyloid cascade hypothesis, targeting amyloidogenic processing of APP is still considered a valid strategy to develop disease-modifying AD therapies (Zhao et al., 2020). New evidence continues to emerge to support the idea that deficiencies in APP trafficking and clearance of Aβ peptides is the initiating event of AD pathogenic processes (Tan and Gleeson, 2019; Zhao et al., 2020). Knowing the importance of protein glycosylation in mediating a plethora of biological functions (Krištić and Lauc, 2017; Akasaka-Manya and Manya, 2020) and considering the fact that most known AD-related molecules are either modified with glycans or play a role in glycan regulation, glycobiology may represent an interesting new insight into the understanding of AD, and a potential for new therapeutic approaches (Haukedal and Freude, 2021). The altered glycan profile of APP in the brain and cerebrospinal fluid (CSF) of AD patients versus healthy controls (Påhlsson et al., 1992; Chun et al., 2017; Boix et al., 2020; Moran et al., 2021) has been reported. Particularly, changes in *O*-glycosylation have been related to differences in APP processing and Aβ generation (Kitazume et al., 2010; Akasaka-Manya et al., 2017; Liu et al., 2017). APP695 is modified by a number of *O*-glycosylation moieties in several sites, both for mucin-linked *O*-glycans (*O*-GalNAc or *N*-acetylgalactosamine) and *O*-GlcNAc (*N*-acetylglucosamine) as observed in Chinese hamster ovary cells (CHO) and human CSF (Perdivara et al., 2009; Halim et al., 2011). *O*-GlcNAcylation has been shown to influence APP cleavage by increasing the nonamyloidogenic processing by α-secretase and reducing the secretion of Aβ *in vitro* and *in vivo* (Jacobsen and Iverfeldt, 2011; Yuzwa and Vocadlo, 2014; Chun et al., 2015, 2017). *O*-GalNAcylation is more abundant on APP, with extended and/or sialylated *O*-glycans occupying the region close to the β-secretase cleavage site (M^671^∼D^672^) of APP (Shi et al., 2021), suggesting its possible role in APP ectodomain shedding and Aβ production (Akasaka-Manya et al., 2017; Nakamura and Kurosaka, 2019). Two *O*-glycosylation sites, Thr^663^ and Ser^667^, located at the *N*-terminal side of β-secretase cleavage site have been reported to contain α-linked terminal GalNAc structure (Shi et al., 2021). Glycosylation on this region has been found to suppress the APP processing (Chun et al., 2015; Akasaka-Manya et al., 2017). In addition, a recent study has shown that the unique Tyr-*O*-glycosylation induces Aβ42 to form less stable fibrils, that are more susceptible towards degradation by extracellular degradation enzymes (Liu et al., 2021). The sialic acid-capped glycans, as found in the CSF samples, would likely further promote inhibition of formation of the typical β sheet-derived fibrils (Liu et al., 2021). We have previously demonstrated that a simple *O*-GalNAc modification on the Tyr^681^ residue of Aβ can induce a conformational change, provide protection from β-secretase, and slightly improve the nonamyloidogenic processing by α-secretase (Singh et al., 2021). However, in the presence of the Swedish mutation, the amyloidogenic processing by β-secretase was drastically increased (Singh et al., 2021). To date, a stimulating and inhibiting effects of glycosylation on enzyme activity have been reported (Goettig, 2016; Goth et al., 2018). Thus, a better understanding of the role of *O*-glycosylation on the balance between production and clearance of Aβ peptides is necessary to decipher the role of *O*-glycosylation in the initiating events of AD pathogenic processes.

In this study, we synthesized APP model (glyco)peptides containing the Aβ-(1–9) fragment, with extended *N*-terminal domain to incorporate the β-secretase cleavage site with or without the Swedish mutation (Lys^670^Asn/Met^671^Leu) and Thr^663^and/or Ser^667^ *O*-glycosites, respectively. These analogues were characterized for their secondary structure content using CD spectroscopy. The roles of *O*-glycosylation and/or Swedish mutation on proteolytic processing by β-secretase and the aggregation kinetics of Aβ40 in the absence and presence of APP (glyco)peptides were explored, respectively. Our results demonstrate a unique role of mucin-type *O*-glycosylation on APP’s secondary structure, proteolytic cleavage, and aggregation properties and offer an important insight into glycosylation driven changes of the intrinsic properties of APP derived peptides.

## Methods

### Materials

Tentagel S RAM resin was obtained from Advanced ChemTech (Louisville, KY). Fmoc-protected amino acids, and coupling reagents, 1-hydroxybenzotriazole (HOBt) and 2-(6-chloro-1H-benzotriazol-1-yl)-1,1,3,3-tetramethylaminium hexafluorophosphate (HCTU), for peptide synthesis, were purchased from Chem-Impex (Wood Dale, IL). *N, N′*-Diisopropylethylamine (DIPEA) was purchased from Acros Organics (Thermo Fisher Scientific, Waltham, MA). Trifluoroacetic acid (TFA), thioanisole, and all solvents (DCM, DMF, acetonitrile, and water) were of HPLC grade and purchased from Fisher Scientific (Atlanta, GA) or Sigma-Aldrich (St. Louis, MO). PBS buffer was prepared using sodium phosphate (mono- and dibasic) from Fisher Scientific (Pittsburg, PA). The *O*-glycosylated GalNAc building blocks of Ser **1** and Thr **2** for glycopeptide synthesis were prepared as published previously by our group (Singh et al., 2020; Beckwith et al., 2021). Recombinant human BACE-1 (rhBACE-1) and BACE-1 fluorogenic peptide substrate IV (MCA-Ser-Glu-Val-Asn-Leu-Asp-Ala-Glu-Phe-Arg-Lys(DPN)-Arg-Arg-NH2) were from R&D Systems (catalog #ES004 and #931-AS, respectively).

### Synthesis of APP (Glyco)peptides

All peptide analogs of APP were synthesized using standard Fmoc chemistry and solid-phase peptide synthesis (SPPS) on a PS3 automated peptide synthesizer (Protein Technologies Inc., Tucson, AZ). The amino acid couplings on the synthesizer were done using a 4-fold excess of amino acids, HOBt, and HCTU, in the presence of 0.4 M*N*-methylmorpholine (NMM) in DMF. The Fmoc protecting group was removed using 20% piperidine in DMF. For glycopeptides, at the desired site of glycosylation, the Fmoc-protected pentafluorophenyl ester of Ser-*O*-GalNAc **1**) and/or Thr-*O*-GalNAc **2**) was coupled manually using a 1.5-fold excess, in the presence of DIPEA (pH 8) for 16 h. After coupling was confirmed using the ninhydrin test, the remainder of the peptide’s amino acid sequence was completed on the PS3. All the (glyco)peptides were cleaved from the resin using a TFA/thioanisole/water mixture in 95:2.5:2.5 ratio for 3 h, followed by precipitation in cold methyl-*tert-*butyl-ether (MTBE) to precipitate the crude (glyco)peptide. For glycopeptides, the acetylated crude was deprotected using 0.01 M NaOH solution for 15 min to remove all the *O*-acetyl groups on the glycan moiety attached to the peptide sequence. Lastly, the crude was lyophilized to yield the final crude deacetylated glycopeptide or its nonglycosylated counterpart.

### Purification and Characterization of APP (Glyco)peptides

Purification of all (glyco)peptides and their corresponding analyses were performed on a 1,260 Agilent Infinity system. The analytical RP-HPLC method uses a Phenomenex Aeris Peptide C18 column (150 mm × 4.6 mm, 3.6 *μ*m, 100 Å) at 0.8 ml/min flow rate or a Vydac Denali C18 column (250 mm × 4.6 mm, 5 *μ*m, 120Å) at 1 ml/min flow rate, with 0.1% TFA in water (A) and 0.1% TFA in acetonitrile (B) as the eluents. The elution gradient for analytical RP-HPLC purification was 0–60% B over 30 min. The preparative RP-HPLC method uses the Grace Vydac monomeric C18 column (250 mm × 22 mm, 15–20 μm, 300Å) at 10 ml/min flow rate, with 0.1% TFA in water (A) and 0.1% TFA in acetonitrile (B) as the eluents. The elution gradient for preparative RP-HPLC purification was 0–50% over 110 min. The (glyco)peptides were detected at 214 nm by using a UV-Vis detector (Agilent 1,260 Infinity DAD). Purified (glyco)peptides were characterized by matrix-assisted laser desorption ionization time-of-flight mass spectrometry (MALDI-TOF MS) with a Voyager-DE STR system or a Bruker Microflex system, using α-cyano-4-hydroxycinnamic acid as matrix.

### Conformational Analysis of APP (Glyco)peptides

All (glyco)peptides were analyzed for their secondary structure using circular dichroism (CD) spectroscopy on a Jasco-810 spectropolarimeter (Jasco, Easton, MD) in three solvent systems: water, 10 mM sodium phosphate buffer (pH 7.4), and 50% trifluoroethanol (TFE) in water (v/v) mixture. The CD spectra were recorded using a quartz cell of 1 mm optical path length over a wavelength range of 180–25 nm with a scanning speed of 100 nm/min and a response time of 4 s at 25°C. A concentration of 0.065 mg/ml, determined using the analytical RP-HPLC method, gave the lowest signal-to-noise ratio for all (glyco)peptides. All spectra were baseline-corrected to account for the signal contribution from solvent and then converted into molar ellipticity (deg cm^2^ dmol^−1^) (Sreerama and Woody, 2000). Lastly, the percentages of all secondary structures were determined using the BeStSel method (Micsonai et al., 2018; Singh et al., 2021).

### Proteolytic Activity and Analysis of APP (Glyco)peptides With BACE-1

All APP-based substrates were prepared as 10 mM stocks in DMSO. Before proteolysis, the activity of BACE-1 was verified by the reaction with the fluorogenic BACE-1substrate Mca-SEVNLDAEFRK(Dnp)RR-NH_2_ (Koike et al., 1999) as per the manufacturer’s instructions. For the proteolysis assay, APP-based substrates were diluted in BACE-1 activity buffer (0.1 M sodium acetate, pH 4.0) to the final assay concentration of 100 μM. BACE-1 was diluted to 50 nM final concentration. Reactions were incubated for 24 h at 37°C in the dark. After the incubation period, the enzyme cleavage solutions containing APP-based substrates and BACE-1 were analyzed using the analytical RP-HPLC method on the Aeris C18 column with 0.1% TFA in water (A) and 0.1% TFA in acetonitrile (B) as eluents and 0–60% B as the elution gradient over 30 min with a flow rate of 0.8 ml/min, and detection at 214 nm or the Vydac Denali C18 column with 0.1% TFA in water (A) and 0.1% TFA in acetonitrile (B) as eluents and 0–60% B as the elution gradient over 30 min with a flow rate of 1 ml/min, and detection at 214 nm. The identity of intact and cleaved (*N*- and *C*-terminal) fragments of (glyco)peptides in the absence and presence of BACE-1 (β-secretase) was confirmed by MALDI-TOF and their percentages were evaluated by the integration of the RP-HPLC peaks (averaged from two injections).

### Preparation of Aβ40 Peptide for Aggregation Kinetics Assay With APP (Glyco)peptides

Aβ40 peptide was synthesized on a PS3 solid phase peptide synthesizer (Protein Technologies Inc., Woburn, MA) using the standard Fmoc strategy. The resulting crude peptide was purified by reversed-phase high-performance liquid chromatography (RP-HPLC) using a C18 column and characterized by matrix-assisted laser desorption ionization (MALDI) mass spectrometry. The peptide was monomerized as described previously before use (Liu et al., 2018). Lyophilized peptide powder was dissolved in aqueous NaOH solution (2 mM), and the pH was adjusted to ∼11 by using 100 mM NaOH solution. The solution was sonicated for 1 h in an ice−water bath and then filtered through a 0.22 μm filter (Millipore) and kept on ice before use. The concentration of the peptide solution was determined by using the tyrosine UV absorbance at 280 nm (ε = 1280 M^−1^ cm^−1^).

### Aggregation Kinetics Assay of Aβ40 With APP (Glyco)peptides

The aggregation kinetics of Aβ40 in the absence and presence of APP (glyco)peptides was performed using ThT binding assay. The monomerized Aβ40 peptide solution was diluted to a final concentration of 10 µM in 50 mM phosphate buffer (pH 7.4) and 20 µM ThT dye. For the co-incubation assays with APP (glyco)peptides, APP stock solutions of 120 µM were added to the prepared Aβ40 for final (glyco) peptide concentrations of 10 µM or 50 µM and Aβ40 concentration of 10 µM in 50 mM phosphate buffer (pH 7.4) with 20 µM ThT dye. 100 µL of each prepared solution was run in triplicate of a 96-well microplate (Costar black, clear bottom). The plate was sealed with a microplate cover and loaded into a Gemini SpectraMax EM fluorescence plate reader (Molecular Devices, Sunnyvale, CA) and incubated at 37°C. The ThT fluorescence was measured from the bottom of the plate at 10 min intervals, with 5 s of shaking, and with an excitation and emission wavelengths of 440 and 480 nm, respectively. Error bars of triplicate samples are shown for the particular data points.

### AFM Analysis of Aβ40 With APP (Glyco)peptides

AFM was employed to monitor the morphological changes of Aβ40 incubated in the absence and presence of tyrosine glycosylated (**14** and **16**) and nonglycosylated APP analogues (**13** and **15**). Aliquots (15 µL) of Aβ40 solutions were collected directly from the aggregation kinetics assay and spotted onto the surface of the freshly cleaved mica surface (5 mm × 5 mm) on solid support at room temperature. Before the measurement, the samples were covered and dried in a vacuum desiccator overnight. The AFM images were acquired in tapping mode with an area of 4 μm^2^, using AFM workshop TT-2 (Hilton Head Island, SC) with MikroMash NSC 15/Al BS silicon cantilevers (MikroMash, Watsonville, CA). The AFM images were further visualized and analyzed using the Gwyddion software.

## Results and Discussion

### APP-Based (Glyco)peptide Synthesis and Characterization

**SCHEME 1:**
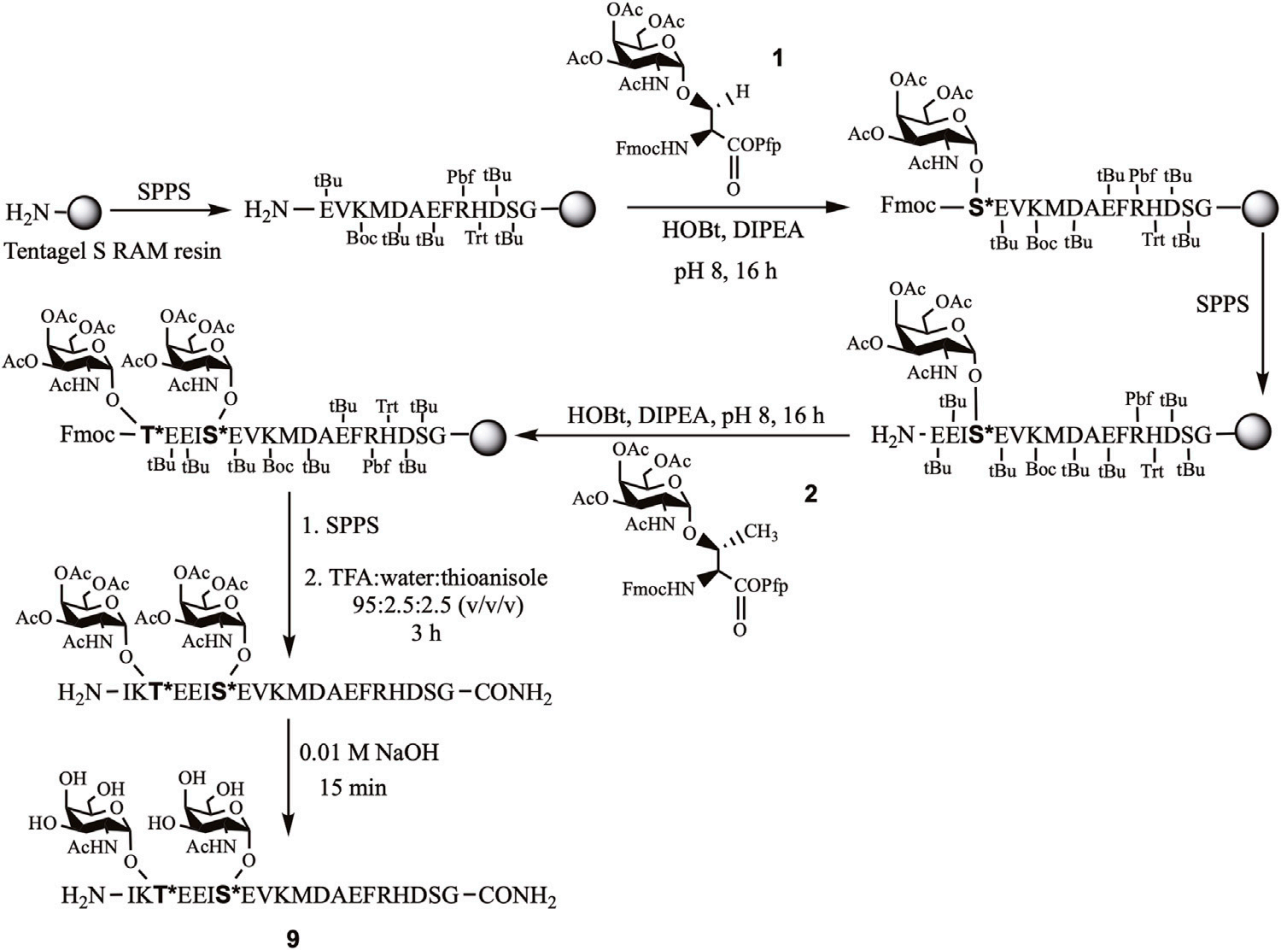
Stepwise synthesis of APP glycopeptide, APP^661-680^-T*, S* (**9**).

The short APP (glyco) peptide fragments, part of the APP 661–680 region, were prepared. The amino acid sequence included Aβ-(1–9) DAEFRHDSG at the *C*-terminal end and either EISEVKM or EISEVNL (NL = Swedish mutation) at the *N*-terminus to incorporate the β-secretase (BACE-1) cleavage site (M ∼ D or L ∼ D). Further extension of the backbone with the additional four amino acids (IKTE), furnished a platform for site-specific *O*-glycosylation of Thr^663^ and Ser^667^ residues that may impact APP’s proteolytic processing due to their proximity to the BACE-1 cleavage site (Goth et al., 2018; Nakamura and Kurosaka, 2019). Hence, APP glycopeptides bearing “mucin-type” *O*-glycosylation, α-*N*-acetylgalactosamine (GalNAc), on Thr^663^ and/or Ser^667^ and their nonglycosylated counterparts were prepared using standard Fmoc-based automated solid-phase peptide chemistry (Scheme 1). For glycopeptides, the building block approach was used for the incorporation of *O*-glycosylated Ser **1** and/or Thr **2** in the sequence. The organic synthesis of the building blocks was achieved using our previously published protocols (Singh et al., 2020; Beckwith et al., 2021). The purity of the Ser/Thr building blocks **1** and **2**, respectively, were confirmed by RP-HPLC and MALDI-TOF mass spectrometry. NMR spectra ascertained the α-linkage (Singh et al., 2020; Beckwith et al., 2021).

Automated solid-phase peptide synthesis (SPPS) approach on Tentagel S RAM resin was used to assemble the APP (glyco)peptides. For glycopeptides, the *O*-glycosylated Ser/Thr building blocks one and two, respectively, were manually coupled at the desired site of glycosylation, Thr^663^ and/or Ser^667^, of the growing peptide chain. After completion of the (glyco)peptide sequence, the resin was treated with trifluoroacetic acid (TFA), with water and thioanisole as scavengers. The crude acetylated glycopeptides were further deprotected under basic conditions to remove acetyl groups from the glycan moiety and obtain final deacetylated glycopeptides (**4, 6, 8, 9, 11**, and **12**). The corresponding nonglycosylated peptides (**3, 5, 7**, and **10**) were also prepared as above except for introducing usual Fmoc-Thr(tBu)/Ser(tBu)-OH amino acids, instead of their *O*-glycosylated analogs (Scheme 1). (Glyco)peptides **3–12** were obtained in high purity, as indicated by their RP-HPLC elution profiles and MALDI-TOF MS analysis (Table 1 and Supplementary Material S2–S11). As expected, the RP-HPLC analysis revealed difference in retention time (*t*_R_) between the Swedish-mutated peptide analogs and their native pairs. The Swedish-mutated (glyco)peptides **5, 6, 10–12** exhibited a 1.5 min (on average) longer *t*_R_ compared to their native counterparts **3, 4, 7-9**, respectively, due to increased hydrophobicity of the peptide sequence (Table 1) (Mant et al., 1989; Singh et al., 2021). In contrast, the addition of the GalNAc moiety at either Ser^667^ (**4** and **6**) or Thr^663^ (**8** and **11**) residue resulted in a decrease in the *t*_R_ by 0.4 min (on average) compared to their nonglycosylated counterparts **3, 5, 7**, and **10**, respectively, due to increased hydrophilicity of the glycopeptide sequences (Table 1) (Singh et al., 2021). The attachment of GalNAc moiety at both glycosylation sites (**12**), further decreased the *t*_R_ by 0.5 min compared to its monoglycosylated counterpart **11** (*t*_R_ = 18.9 min, Table 1).

**TABLE 1:** Characterization of APP (glyco)peptides **3–16** by analytical RP-HPLC and MALDI-MS^a^.

APP (Glyco)peptides	Sequence	RP-HPLC	MALDI-TOF MS (M+H)^+^	
		t_*R*_ (min)	Calculated (Da)	Observed (Da)
APP^665-680^ (**3**)	EISEVKM∼DAEFRHDSG	14.0	1848.98	1849.10
APP^665-680^-S* (**4**)	EIS*EVKM∼DAEFRHDSG	13.6	2051.98	2051.68
APP^665-680^(NL) (**5**)	EISEVNL∼DAEFRHDSG	15.6	1816.88	1816.83
APP^665-680^(NL)-S* (**6**)	EIS*EVNL∼DAEFRHDSG	15.0	2019.88	2018.54
APP^661-680^ (**7**)	IKTEEISEVKM∼DAEFRHDSG	14.6	2,320.54	2,320.19
APP^661-680^-T* (**8**)	IKT*EEISEVKM∼DAEFRHDSG	14.4	2,523.54	2,527.26
APP^661-680^-T*, S* (**9**)	IKT*EEIS*EVKM∼DAEFRHDSG	16.9b	2,726.54	2,728.80
APP^661-680^(NL) (**10**)	IKTEEISEVNL∼DAEFRHDSG	16.0	2,288.43	2,290.09
APP^661-680^(NL)-T* (**11**)	IKT*EEISEVNL∼DAEFRHDSG	15.6/18.9^b^	2,491.43	2,491.32
APP^661-680^(NL)-T*, S* (**12**)	IKT*EEIS*EVNL∼DAEFRHDSG	18.4^b^	2,694.43	2,696.55
APP^661-694^ (**13**)^c^	IKTEEISEVKM∼DAEFRHDSGYEVHHQK∼LVFFAED	17.6	4,062.09	4,062.27
APP^661-694^-Y* (**14**)^c^	IKTEEISEVKM∼DAEFRHDSGY*EVHHQK∼VFFAED	17.1	4,265.14	4,265.41
APP^661-694^(NL) (**15**)^c^	IKTEEISEVNL∼DAEFRHDSGYEVHHQK∼LVFFAED	18.2	4,030.02	4,029.37
APP^661-694^(NL)-Y* (**16**)^c^	IKTEEISEVNL∼DAEFRHDSGY*EVHHQK∼LVFFAED	17.8	4,231.99	4,232.71

a T*/S*/Y* = Thr^663^/Ser^667^/Tyr^681^ O-linked GalNAc, NL, swedish mutation, M∼D and L∼D = β-secretase cleavage site and K∼L = α-secretase cleavage site. RP-HPLC, conditions and MALDI-TOF MS, analyses are described in the Supplementary Material S2–S9. Retention times (*t*_R_) are given in minutes.

b RP-HPLC, conditions and MALDI-TOF MS, analyses are described in the Supplementary Material S9–S11.

c Reported in Singh et al., 2021.

### Conformational Properties of APP (Glyco)peptides

**FIGURE 1:**
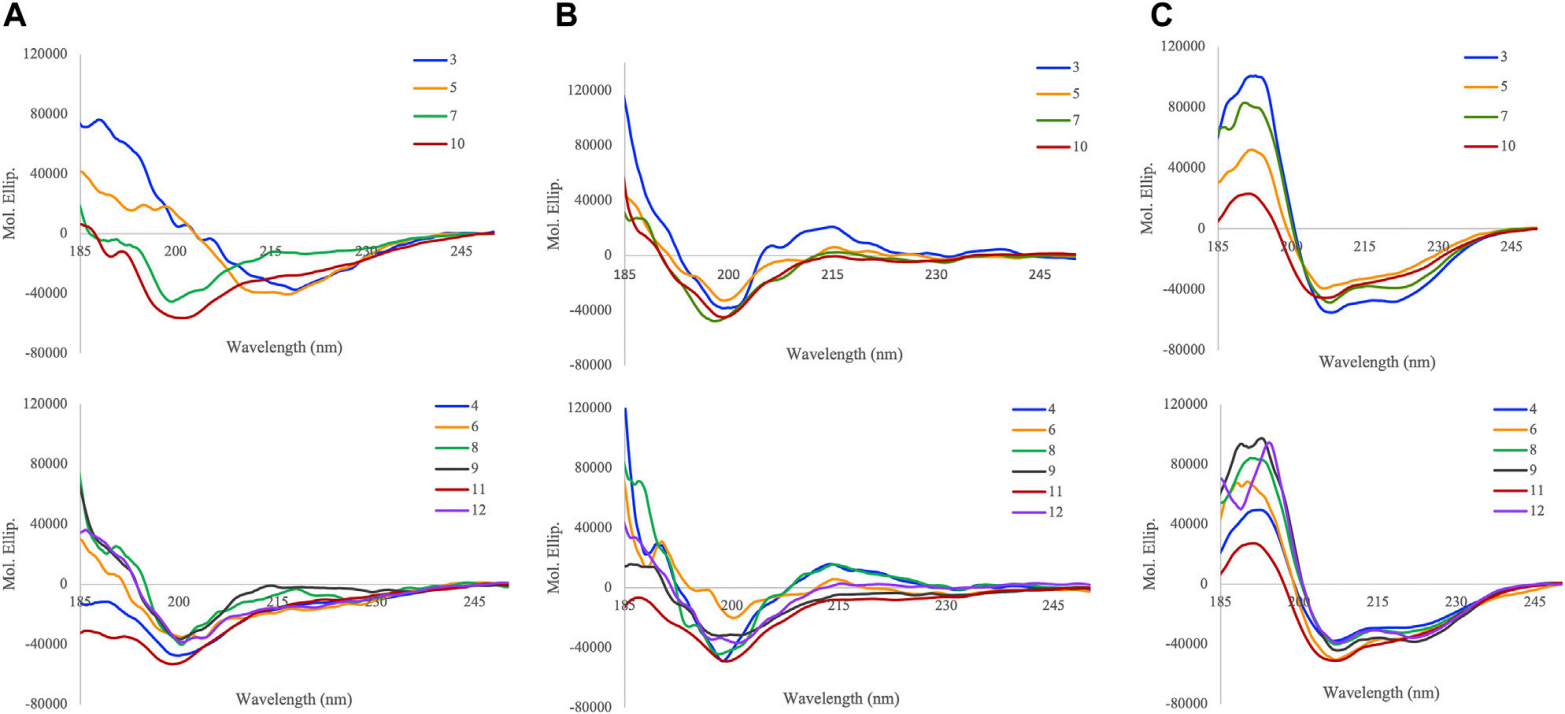
Circular dichroism spectra of APP (glyco)peptides **3–12** in **(A)** water **(B)** 10 mM sodium phosphate buffer, pH 7.4, and **(C)** TFE/water = 1:1 (v/v) at 25°C.

To study the role of *O*-glycosylation on the conformation of APP glycopeptides, circular dichroism (CD) spectroscopy was used to probe the secondary structure in three different solvents, water, sodium phosphate buffer (10 mM, pH 7.4), and 50% trifluoroethanol (TFE) in water (v/v) (Figure 1). The spectra were further analysed for secondary structure estimations by Beta Structure Selection (BeStSel) method, specifically designed for the analysis of beta sheet-rich proteins (Table 2) (Micsonai et al., 2015, 2018).

**TABLE 2:** Summary of the secondary content (%) present in APP (glyco)peptides **3–12** determined by BeStSel for CD spectra obtained in (A) water (B) 10 mM sodium phosphate buffer, pH 7.4, and (C) TFE/water = 1:1 (v/v)^a^.

APP (glyco)peptides	α-H (%)	β-AP (%)	β-P (%)	β-T (%)	RC (%)
(A)					
** **APP^665-680^ (**3**)	14.8	59.9	18.1	7.2	0.0
** **APP^665-680^-S* (**4**)	0.0	0.0	0.0	12.9	87.1
** **APP^665-680^(NL) (**5**)	34.5	21.9	35.9	0.0	7.7
** **APP^665-680^(NL)-S* (**6**)	19.2	30.8	0.0	8.6	41.4
** **APP^661-680^ (**7**)	0.0	8.3	0.0	6.6	85.1
** **APP^661-680^-T* (**8**)	1.4	5.4	0.0	5.7	87.4
** **APP^661-680^-T*, S* (**9**)	12.8	48.2	0.0	16.6	22.4
** **APP^661-680^(NL) (**10**)	0.0	9.3	0.0	0.0	90.7
** **APP^661-680^(NL)-T* (**11**)	0.0	0.0	0.0	12.5	87.5
** **APP^661-680^(NL)-T*, S* (**12**)	18.0	45.8	0.0	9.2	27.0
(B)					
** **APP^665-680^ (**3**)	6.1	39.8	0.0	19.8	34.3
** **APP^665-680^-S* (**4**)	9.3	33.6	0.0	14.9	42.2
** **APP^665-680^(NL) (**5**)	6.7	34.6	0.0	23.2	35.6
** **APP^665-680^(NL)-S* (**6**)	0.0	41.3	0.0	33.5	25.3
** **APP^661-680^ (**7**)	8.4	36.1	0.0	15.7	39.8
** **APP^661-680^-T* (**8**)	8.8	42.6	0.0	18.3	30.4
** **APP^661-680^-T*, S* (**9**)	9.5	24.6	0.0	17.1	48.7
** **APP^661-680^(NL) (**10**)	0.0	34.4	0.0	15.0	50.6
** **APP^661-680^(NL)-T* (**11**)	0.0	16.6	0.0	6.6	76.8
** **APP^661-680^(NL)-T*, S* (**12**)	3.6	47.9	0.0	17.2	31.2
(C)					
** **APP^665-680^ **(3)**	66.2	2.7	12.6	0.9	17.6
** **APP^665-680^-S* **(4)**	52.2	0.0	0.0	2.9	44.9
** **APP^665-680^(NL) **(5)**	59.9	3.5	3.2	0.0	33.3
** **APP^665-680^(NL)-S* **(6)**	66.7	0.0	0.7	0.0	32.6
** **APP^661-680^ **(7)**	88.7	0.0	0.0	3.2	8.1
** **APP^661-680^-T* **(8)**	85.7	0.0	0.0	6.0	8.3
** **APP^661-680^-T*, S* **(9)**	94.4	0.0	0.0	5.6	0.0
** **APP^661-680^(NL) **(10)**	36.8	9.7	7.5	0.0	46.0
** **APP^661-680^(NL)-T* **(11)**	40.3	6.1	4.7	0.0	48.9
** **APP^661-680^(NL)-T*, S* **(12)**	86.2	0.0	3.1	6.5	4.3

a The content is divided into α-helix (α-H), anti-parallel β-sheet (β-AP), parallel β-sheet (β-P), β-turn (β-T), and random coil (RC).

In water, the addition of *N*-terminal fragment, EISEVKM (native) or EISEVNL (Swedish-mutated), to Aβ-(1–9) in **3** and **5**, respectively, exhibited characteristics of β-sheet structure that closely resembled the CD spectra of other Aβ variants in water (J. and G., 1991; Juszczyk et al., 2005; Lambermon et al., 2005). However, further extension of the *N*-terminal fragment with the additional four amino acids (IKTE) in native **7** and Swedish-mutated **10**, respectively, increased the overall hydrophilicity of Aβ-(1–9) and caused a conformation shift from β-sheet to random coil (Figure 1A). This agrees with our previous work where addition of the *N*-terminal fragment, IKTEEISEVKM (native), to β-sheet-forming Aβ-(1–23) peptide in **13**, was also largely disordered (Singh et al., 2021). Deconvolution of the spectra revealed that native peptide **3** had the highest amount of β-structure (85.2%, anti-parallel β-sheet, parallel β-sheet, and β-turn), with the majority being anti-parallel β-sheet (59.9%), and the remaining being α-helix (14.8%) (Table 2). The presence of the Swedish double-mutation in **5**, resulted in an increase in α-helix and decrease in the β-structure amounts (34.5 and 57.8%, respectively). The distribution of α-helix and β-structure content in five is similar to the Swedish-mutated Aβ-(1–23) peptide **15**, (Juszczyk et al., 2005; Singh et al., 2021). CD spectra clearly indicates that regardless of the Swedish mutation, addition of the *N*-terminal fragment, IKTEEISEVKM or IKTEEISEVNL, to Aβ-(1–9) significantly increased the percentage of random coil in **7** and **10** (85.1 and 90.7%, respectively). Site-specific *O*-glycosylation of Ser^667^ in glycopeptides **4** and **6** caused their structure to be largely disordered (87.1% and 41.4%, respectively) with complete loss of parallel β-sheets compared to their nonglycosylated counterparts **3** and **5**, respectively (Figure 1A). This effect was more pronounced for the native glycopeptide **4** where β-structure along with α-helix was significantly reduced (12.9% and 0%, respectively). On the other hand, the Swedish-mutated glycopeptide **6** was less prone to change in its β-structure and α-helix amounts (39.4% and 19.2%, respectively) (Table 2). Also, the site-specific *O*-glycosylation of Thr^663^ in glycopeptides **8** and **11** did not significantly change the secondary conformation compared to their nonglycosylated counterparts **7** and **10**, respectively, that were already largely disordered in water (85–90%). Hence, we were able to confirm that the addition of a single GalNAc moiety in APP 661–694 derived glycopeptides is able to break ordered secondary structures and cause it to be disordered in water (Singh et al., 2021). Interestingly, by increasing the glycan valency in glycopeptides **9** and **12**, the amount β-structure increased (64.8 and 55.0%, respectively) and random coil decreased (22.4 and 27.0%, respectively). Therefore, glycan valency is an important determinant of the secondary structure of APP glycopeptides in water.

To evaluate secondary structure in a more physiologically relevant buffer setting, the CD spectra of (glyco)peptides were recorded in sodium phosphate buffer of low ionic strength (10 mM, pH 7.4). As expected, the (glyco)peptides indicated the presence of an unfolded state and were partially disordered with some β-structure properties. Deconvolution of the spectra revealed a prominent presence of random coil (25–77%) and antiparallel β-sheet (17–48%), followed by β-turn (7–34%), and lastly, α-helix (0–10%) (Figure 1B and Table 2) (Hortschansky et al., 2005; Tew et al., 2008; Singh et al., 2021). The Swedish-mutated diglycosyated peptide **12** showed the highest amount of antiparallel β-sheet (47.9%) whereas its monoglycosylated counterpart **11** had the highest amount of random coil (76.8%) in this solvent system. Notable, in the absence of parallel β-sheets, the ratio of the remaining structural elements varied depending on the modifications (Swedish mutation and/or glycosylation) incorporated into the peptide sequences (Figure 1B and Table 2). These findings are in agreement with the previously reported CD data for Aβ peptides of different length; the Aβ-(1–42) peptide exhibited slightly higher α-helix and random coil content compared to Aβ-(1–16) peptide that has higher β-sheet and β-turn content (Tew et al., 2008). Likewise, our previously reported (glyco)peptides containing longer Aβ-(1–23) fragment **(13–16)** showed higher α-helix and random coil, and lower antiparallel β-sheet content, in sodium phosphate buffer (Singh et al., 2021) compared to (glyco)peptides analysed in this study that contain shorter Aβ-(1–9) fragment **(3–12)**. Hence, Aβ occurs in various isoforms that differ by the number of residues at the *C*-terminal end of the peptides, which impacts the secondary structural preferences of the peptides in solution.

APP is an integral membrane protein whose behaviour can be modified by molecules such as trifluoroethanol (TFE) that partition the membrane-water interface and change the physiochemical properties of the lipid bilayer (Barry and Gawrisch, 1994; Özdirekcan et al., 2008). We have previously investigated the effects of TFE on a molecular level using CD spectroscopy to obtain an account of the α-helix-forming potential of model (glyco)peptides **13–16** containing the Tyr^681^ *O*-linked glycosylation in Aβ-(1–23) region of APP (Singh et al., 2021). Upon addition of TFE into water (1:1, v/v), the (glyco)peptides showed a significant increase in α-helix and random coil content and decrease in β-structure content (Figure 1C). Upon further analysis, shorter native peptide **3** had slightly higher α-helix content (66.2%) than its Swedish-mutated counterpart **5** (59.9%), however, this difference was larger between the longer native peptide **7** (88.7%) and its Swedish-mutated counterpart **10** (36.8%) (Table 2). Regardless of the length of Aβ fragment, the Swedish mutation significantly reduced the amount of α-helix and increased β-sheet and random coil secondary structure elements in this solvent system. Similarly, site-specific *O*-glycosylation of Thr^663^ and Tyr^681^ (Singh et al., 2021) slightly decreased the α-helix and increased the random coil content in native glycopeptides, **8** and **14** respectively, whereas it increased α-helix and decreased random coil content in Swedish-mutated glycopeptides, **11** and **16** respectively, with this effect being more pronounced for **16**. Lastly, regardless of the Swedish mutation, the attachment of two GalNAc moieties on Thr^663^ and Ser^667^ within the peptide sequence drastically increased the α-helix content in **9** and **12** (94.4% and 86.2%, respectively), in this solvent system. These findings suggest that in membrane-mimicking conditions, excess *O*-glycosylation can hamper the effects of the Swedish mutation on secondary structure and prompt it to become largely α-helical.

### BACE-1 Activity of APP (Glyco)peptides

Glycosylation can alter substrate recognition and impact enzyme activity in either a positive (enhancing) or negative (inhibiting) manner (Goettig, 2016; Goth et al., 2018). The mucin-type *O*-linked glycosylation of a protein may not only affect its conformation but also affect its transport and localization in the cell (Matsuura et al., 1989; Chen et al., 2002). Proteases are highly regulated by post-translational modifications and drive fate, localization, and activity of many proteins (Bond, 2019). Certain mutations can also affect the subcellular localization of the cleavage event by crucial proteases and mediate a different cellular mechanism for the protein (Haass et al., 1995). We have previously reported that the Swedish mutation is an important criterion for enhancing both ADAM10 (α-secretase) and BACE1 (β-secretase) cleavage rates of Aβ-(1–23) model (glyco)peptides **13–16**, where site-specific mucin-type *O*-linked glycosylation of Tyr^681^ residue in **16** further increased BACE-1 driven amyloid pathway (Singh et al., 2021). Therefore, similar enzyme cleavage assays with BACE-1 were set up to explore the effect of the Swedish mutation, length of the amino acid sequence, glycan position and valency on the proteolytic susceptibility of **3–12**. BACE1 produced two fragments upon cleavage of (glyco)peptides, for which yields were determined after 24 h treatment. The yields of intact peptide and fragments were evaluated by the RP-HPLC peaks integration (Table 3, and the Supplementary Material S20–S32).

**TABLE 3:** Proteolytic cleavage of APP (glyco)peptides **3–12** upon treatment with BACE-1 enzyme (KM∼D/NL∼D cleavage site)^a^.

APP (glyco)peptides	BACE-1 activity	
	Recovered (%)	Cleaved (%)
APP^665-680^ **(3)**	95.5	4.48
APP^665-680^-S* **(4)**	98.6	1.40
APP^665-680^(NL) **(5)**	3.82	96.2
APP^665-680^(NL)-S* **(6)**	7.02	93.0
APP^661-680^ **(7)**	96.6	3.40
APP^661-680^-T* **(8)**	91.5	8.46
APP^661-680^-T*, S* **(9)**	95.9	4.12
APP^661-680^(NL) **(10)**	20.3	79.7
APP^661-680^(NL)-T* **(11)**	1.90	98.1
APP^661-680^(NL)-T*, S* **(12)**	3.90	96.1
APP^661-694^ **(13)**	100^b^	0.0^b^
APP^661-694^-Y* **(14)**	100^b^	0.0^b^
APP^661-694^(NL) **(15)**	86.8^b^	13.1^b^
APP^661-694^(NL)-Y* **(16)**	57.4^b^	42.4^b^

a The values were calculated as described in the *Methods* with SD <5% and identity of the fragments was determined by RP-HPLC, analysis and confirmed by MALDI-TOF (see the Supplementary Material S20–S32).

b Reported in Singh et al., 2021.

Regardless of glycosylation, the native Aβ-(1–9) model (glyco)peptides **3** and **4** showed almost full recovery after incubation with BACE-1 (95.5% and 98.6%, respectively). The extension of the native (glyco)peptide sequence at the *N*-terminus with IKTEEISEVKM in **7** and **8** also resulted in a very low BACE-1 proteolysis, 3.4% and 8.5%, respectively. Increasing the glycan valency to two, in native glycopeptide **9**, did not significantly alter cleavage rates (95.9% recovery). It became evident that the presence of the Swedish mutation in five was a driving force for the near complete cleavage of the peptide (96.2%). Interestingly, the extension of the peptide sequence at the *N*-terminus with IKTEEISEVNL in **10** showed a lower amount of the peptide cleaved (79.7%) in comparison to shorter peptide fragment **5**. Thus, a longer *N*-terminal fragment flanking the Aβ region can hamper its proteolytic susceptibility and result in slower cleavage rates by BACE-1. Glycosylation had a more significant effect on the sequence carrying the Swedish mutation, where regardless of the *N*-terminal fragment length, glycan position and valency, BACE-1 treatment resulted in 93–98% cleaved products for glycopeptides **6** and **11–12**. This agrees with our previous findings that apart from the Swedish mutation, the presence of *O*-glycosylation can drive BACE-1 cleavage rates and result in increased Aβ production (Singh et al., 2021).

Other key aspects affecting BACE-1 cleavage rates in the presence of the Swedish mutation were the length of the *C*-terminal Aβ fragment and the glycan position relative to the cleavage site. For example, **16** contains Aβ-(1–23) *C*-terminal fragment and is cleaved to a much lesser extent (42.4%) by BACE-1 than **11** that contains Aβ-(1–9) *C*-terminal fragment (98.1%). Moreover, the relative position of the GalNAc moiety on Thr^663^ and Tyr^681^ in **11** and **16**, respectively, can also influence enzymatic activity. Even though **16** is cleaved to a lesser extent than **11** by BACE-1, the ∼3-fold increase in cleaved products compared to its non-glycosylated counterpart **15** is observed. This further points to a stronger effect of Tyr^681^ *O*-glycosylation on the *C*-terminal Aβ fragment in accelerating BACE-1 activity. Therefore, we can postulate that in the presence of the Swedish mutation, excess *O*-glycosylation proximal to BACE-1 cleavage site can significantly increase the cleavage propensity of the peptides for the amyloid pathway, with this effect being more pronounced when the glycosylation is on the *C*-terminal Aβ side of the cleavage site.

The subcellular localization of BACE-1 cleavage of Swedish-mutated APP differs greatly from that for native APP, where the former is localized to a post Golgi compartment for Aβ generation and outcompetes anti-amyloidogenic processing by α-secretases (Haass et al., 1995; Thinakaran et al., 1996). However, little is known regarding the regulatory role of mucin-type *O*-linked glycosylation of APP on BACE-1 activity, and these results are particularly interesting, since Aβ peptides in CSF of AD patients are heavily glycosylated by mucin-type *O*-linked glycans (Halim et al., 2011). Thus, we can speculate that both Swedish mutation and mucin-type *O*-linked glycosylation increase APP processing because the former provides a better cleavage site for BACE-1, and the latter changes the conformation and increases the sensitivity of the protein to BACE-1.

### Aggregation Kinetics of APP (Glyco)peptides on Aβ40 Fibrillogenesis

**FIGURE 2:**
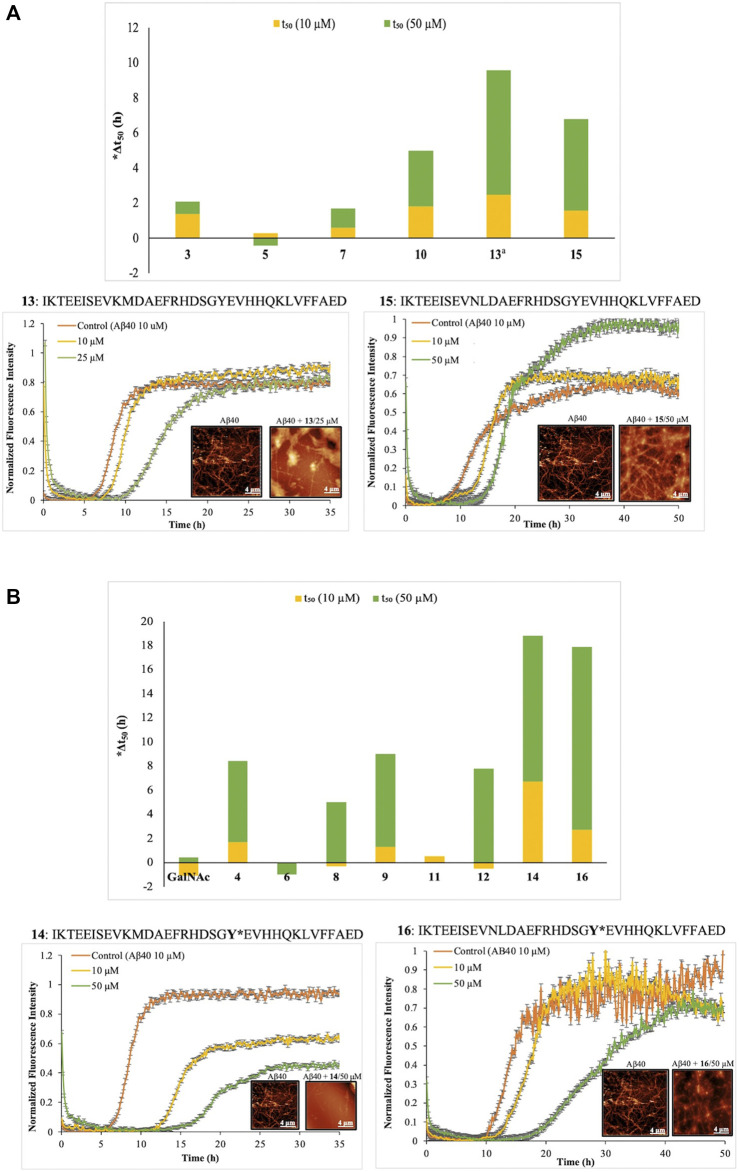
Effect of APP peptides **3, 5, 7, 10, 13**, and **15 (A)** and glycopeptides **4, 6, 8, 9, 11, 12, 14, 16 (B)** on the aggregation kinetics of Aβ40 (10 µM) using ThT fluorescence assay in phosphate buffer (50 mM, pH 7.4) at 37°C. The concentration of (glyco)peptides was 10 and 50 µM ^a^Peptide 13 was run at 10 and 25 µM *Aggregation half-time (Δt_50_) = APP (glyco)peptide t_50_—Aβ40 t_50_. The t_50_ values are means of triplicate kinetics results. Alongside are tapping mode atomic force microscopy images of Aβ40 fibril growth upon 24 h incubation with nonglycosylated peptides, **13** and **15 (A)** and Tyr-*O*-glycopeptides, **14** and **16 (B)**.

We adopted the widely used thioflavin T (ThT) assay (Xue et al., 2017) to investigate how *O*-glycosylation impacts the kinetics of fiber formation of Aβ40, a model peptide for studying the dynamics of protein aggregation. The aggregation kinetic profile of Aβ40 peptide (10 μM) exhibited a typical sigmoidal curve with three different regions: a lag phase associated with nucleation, a rapid growth phase for elongation and polymerization by fibrils, and a final saturation phase dominated with mature fibrils (Figure 2, Supplementary Material S33–S40). At the conditions used in this study, it has been shown that Aβ amyloidogenesis proceeds by a nucleation-dependent polymerization mechanism that involves key soluble oligomeric intermediates (Du et al., 2011; Elbassal et al., 2017). The half time (*t*_50_) of the growth phase of the Aβ40 amyloidogenesis was approximately 8 h (Figure 2, Supplementary Material S33–S40), where *t*_50_ is defined as the time at which the fluorescence intensity reaches the midpoint between the pre- and post-aggregation baselines.

Although recent studies have implicated small soluble oligomers, as the main culprits of Aβ toxicity and AD pathogenesis (Yang et al., 2017), very little is known about the exact mechanism of oligomeric assembly and the conformation of peptides in this early event of Aβ aggregation. In the presence of APP (glyco) peptides **3–16** at two concentrations (10 and 50 µM), the kinetics of aggregation of Aβ40 was described by a sigmoidal curve, with a lag phase that varied depending on the internal modifications of the (glyco)peptides such as the Swedish mutation, site-specific *O*-glycosylation, glycan valency and/or sequence length. The curves reached a plateau after approximately 24 h (Supplementary Material S33–S40). The nonglycosylated peptides **3**, **5**, and **7** displayed no marked effect either on the lag phase (Δt_50_ <2 h, Figure 2A) or the final ThT fluorescence intensity in comparison to control Aβ40 peptide alone (Supplementary Figures S1A–S3A, Supplementary Material S33–S35). Interestingly, we observed a slight delay in the aggregation process by the Swedish-mutated peptide **10** (Δt_50_ = 3.2 h at 50 μM, Figure 2A, Supplementary Figure S4A, Supplementary Material S36) with extended *N*-terminal domain. Increasing the C-terminal domain sequence in **13 and 15**, to include Aβ-(1–23) fragment, led to a much larger effect on the lag phase (Supplementary Figures S5A, S6A, Supplementary Material S38–S39), with Δt_50_ = 7.1 h for **13** (25 µM) and Δt_50_ = 5.2 h for **15** (50 µM) (Figure 2A), indicating an inhibitory effect on Aβ40 aggregation. Notably, we observed a complete saturation of the ThT signal for native peptide **13** at higher concentration (50 µM) (Supplementary Figures S5A,B, Supplementary Material S38). Hence, the aggregation of Aβ40 in the presence of nonglycosylated peptides **3, 5, 7, 10, 13**, or **15** displayed a longer lag phase when the length of the Aβ fragment was increased. The effect of the Swedish mutation was less clear and varied regardless of peptide length.

To evaluate Aβ40 aggregation in the presence of glycopeptides, we first performed kinetics with the free GalNAc sugar (10 and 50 µM) that showed minimal difference in Δt_50_ values (Δt_50_ <0.5 h, Figure 2B). The presence of a single GalNAc moiety on Ser^667^ in native glycopeptide four increased the lag phase substantially (Δt_50_ = 6.7 h at 50 μM, Figure 2B, and Supplementary Figure S1B in the Supplementary Material S33) and delayed Aβ40 aggregation much more compared to its non-glycosylated counterpart **3**. Extension of the *N*-terminal domain sequence in native glycopeptide eight by IKTE, slightly decreased the Δt_50_ value (Δt_50_ = 5 h at 50 μM, Figure 2B, and Supplementary Figure S3B in the Supplementary Material S35) compared to four. However, increasing the glycan valency in nine resulted in gain in inhibition of Aβ40 aggregation and further increase of the lag phase (Δt_50_ = 7.7 h at 50 μM, Figure 2B, Supplementary Figure S3C, Supplementary Material S36). Regardless of the length of the *N*-terminal domain sequence, the presence of the Swedish mutation in **6** and **11** completely suppressed the inhibitory effect of *O*-glycosylation on Aβ40 aggregation exhibited by **8** (Δt_50_ = 0 h at 50 μM, Figure 2B, and Supplementary Figure S2B, S4B in the Supplementary Material S34,S37). A drastic increase in the Δt_50_ value for diglycosylated and Swedish mutated peptide **12** (Δt_50_ = 7.8 h at 50 μM, Figure 2B, and Supplementary Figure S4C in the Supplementary Material S37) clearly indicated that *O*-glycosylation at multiple sites of attachment can overcome the effect of the Swedish mutation and cause Aβ40 to aggregate at slower rates. Consistent with their nonglycosylated versions **13** and **15**, the extension of the Aβ fragment and site-specific *O*-glycosylation of Tyr^681^ in **14** and **16** showed the largest difference in lag phase and strongest inhibition of Aβ40 aggregation profile, with Δt_50_ = 12.1 h for **14** (50 µM) and Δt_50_ = 15.2 h for **16** (50 µM) (Figure 2B, and Supplementary Figures S5C, S6B in the Supplementary Material S39–S40). Along with having a prolonged lag phase, **14** also reduces the final ThT fluorescence intensity dramatically (∼50%), suggesting a significant interference in Aβ40 aggregation. Therefore, our results suggest that *O*-glycosylation inhibits Aβ40 aggregation in a concentration-dependent manner, and this effect is more pronounced when the glycopeptides contain the longer Aβ-(1–23) fragment and GalNAc modification on Tyr^681^(**14** and **16**). It is also important to mention that by reducing the length of the Aβ fragment to Aβ-(1–9), we were able to detect the key differences between the Swedish-mutated (**6** and **11**) and mono-/diglycosylated analogues (**4, 8, 9**, and **12**) on Aβ40 aggregation kinetics.

The morphology of the tyrosine (glyco) peptide aggregates (**13–16**) co-incubated with Aβ40 was examined using atomic force microscopy (AFM). In the absence of (glyco)peptides, Aβ40 forms a dense meshwork of amyloid fibrils (Figure 2). In the presence of native peptide **13** (25 μM) lower density of Aβ40 fibrils is observed (Figure 2A). Similarly, addition of glycosylated counterpart **14** (50 μM), resulted in less fibrils formed, and the ones formed were shorter in size (Figure 2B). This is consistent with the ThT kinetics data for **13** and **14** at 25 and 50 μM, respectively. The addition of the Swedish-mutated peptide **15** (50 µM) to Aβ40 resulted in the fibrilar morphology similar to that of Aβ40 alone (Figure 2A). However, its glycosylated counterpart **16** was able to partially inhibit the fibril formation, resulting in thinly dispersed and less dense Aβ40 fibril (Figure 2B). Thus, as observed in ThT kinetics, the aggregation of Aβ40 can be delayed by **15** and **16** (50 µM), however, the inhibitory potency is not enough to prevent fibril formation under the current conditions.

Our work parallels and directly complements the study by Liu et al., 2021 that shows Aβ42 peptides bearing Tyr^681^ *O*-glycosylation significantly affect both the aggregation and degradation of Aβ42. Furthermore, similar inhibiting activity of Aβ40 and Aβ42 fibrillogenesis by glycation (Emendato et al., 2018; Milordini et al., 2020) and addition of polysaccharides, such as chitosan (Liu et al., 2015; Hao et al., 2017) and heparin sulfate (Wang et al., 2021) was reported.

## Conclusion

In summary, the dynamical interplay between *O*-glycosylation and aggregation affected the structure of peptides and slowed down the aggregation process. The presence of the Swedish mutation led to an increased amount of β-structure in physiological conditions, with the β-secretase activity being drastically increased, and the aggregation process remaining largely unaffected. However, this effect of the Swedish mutation on the (glyco)peptides was overcome by increasing the number of glycosylation sites near the β-secretase cleavage site, increasing the C-terminal domain (Aβ) sequence relative to the β-secretase cleavage site, and/or having Tyr^681^ glycosylated in the Aβ domain, resulting in glycosylation strongly inhibiting the aggregation process of Aβ40. Therefore, our studies demonstrate that *O*-glycosylation typically supports the non-amyloidogenic processing of APP, however, in FAD cases, it can incline towards the amyloidogenic processing of APP, where its fate lies upon the abundance and position of *O*-glycans relative to the β-secretase cleavage site.

## Data Availability

The original contributions presented in the study are included in the article/Supplementary Material, further inquiries can be directed to the corresponding author.
