# Supplementary material for: Mucin-Type O-Glycosylation Proximal to β-Secretase Cleavage Site Affects APP Processing and Aggregation Fate
Source: Front Chem. 2022 Apr 8;10:859822. doi: 10.3389/fchem.2022.859822 (PMC9023740; doi:10.3389/fchem.2022.859822)

Supplementary Material

Mucin-type *O*-Glycosylation Proximal to β-Secretase Cleavage Site Affects APP Processing and Aggregation Fate

YashoNandini Singh[1], Deepika Regmi[1], David Ormaza[1], Ramya Ayyalasomayajula[1], Nancy Vela[1], Gustavo Mundim[1], Deguo Du[1], Dmitriy Minond[2], and Maré Cudic[1]*

[1]Department of Chemistry and Biochemistry, Charles E. Schmidt College of Science, Florida Atlantic University, Boca Raton, Florida 33431, United States
[2]College of Pharmacy and Rumbaugh-Goodwin Institute for Cancer Research, Nova Southeastern University, Fort Lauderdale, Florida 33314, United States

*Correspondence: mcudic@fau.edu

List of Contents

1. RP-HPLC and MALDI-TOF MS spectra of APP (glyco)peptides **3-12** Pages **S2** – **S11**

2. Secondary structure predictions from the CD spectra of **3-12** using the BeStSel method Pages **S12 – S19**

3. Enzyme cleavage assays with APP (glyco)peptides **3-12** by RP-HPLC and MALDI-TOF MS analysis Pages **S20** – **S32**

4. Aggregation kinetic ThT fluorescence curves of APP (glyco)peptides **3-16** coincubated with Aβ40 (**Figures 1-6**) Pages **S33** – **S40**

1. RP-HPLC and MALDI-TOF MS spectra of APP (glyco)peptides **3-12**

(Glyco)peptide analogs were synthesized as shown in **Scheme 1**
S*/T*= Ser(-α-GalNAc) **1**/Thr(-α-GalNAc) **2**

RP-HPLC gradient on Phenomenex Aeris Peptide C18 column (150 x 4.6 mm, 3.6 μm, 100Å) for **3-8**, **10** and **11**:
Eluents were 0.1% TFA in water (A) and 0.1% TFA in acetonitrile (B). The elution gradient was 0-60%B in 30 minutes with a flow rate of 0.8 mL/min. Detection was at λ = 214 nm.

(a) APP$^{665\text{-}680}$, **3**

EISEVKMDAEFRHDSG

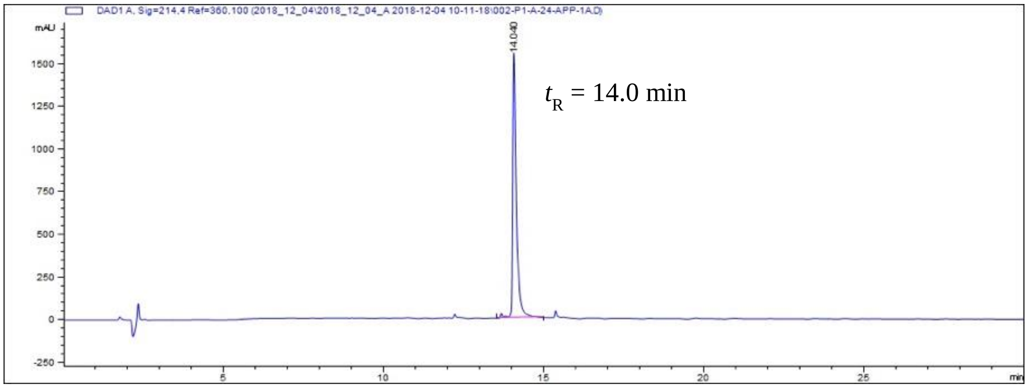

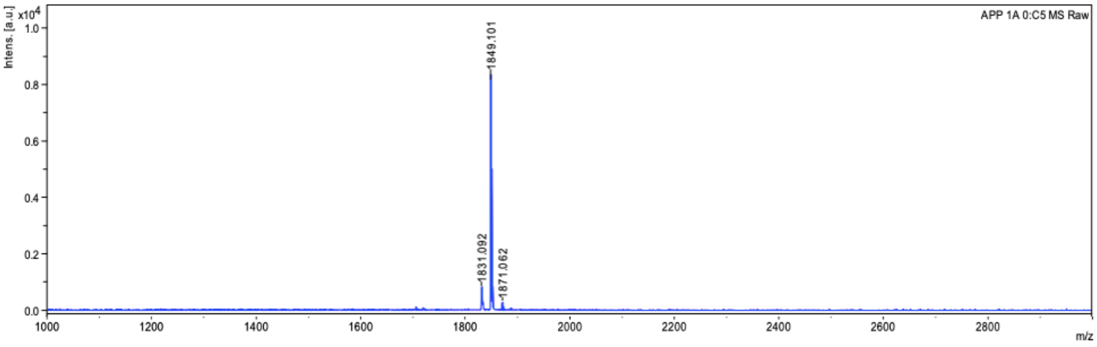

Expected $[M + H]^+$ = 1848.98 Da Observed $[M + H]^+$ = 1849.10 Da

(b) APP$^{665\text{-}680}$-S*, **4**

EI**S***EVKMDAEFRHDSG

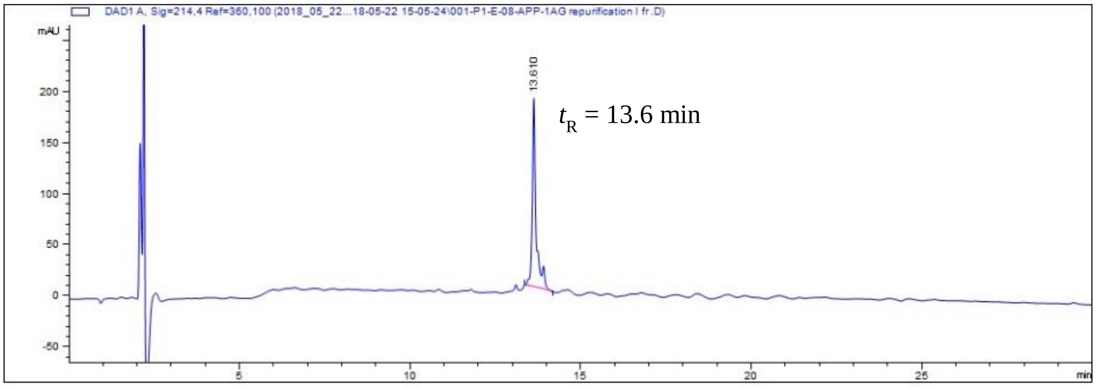

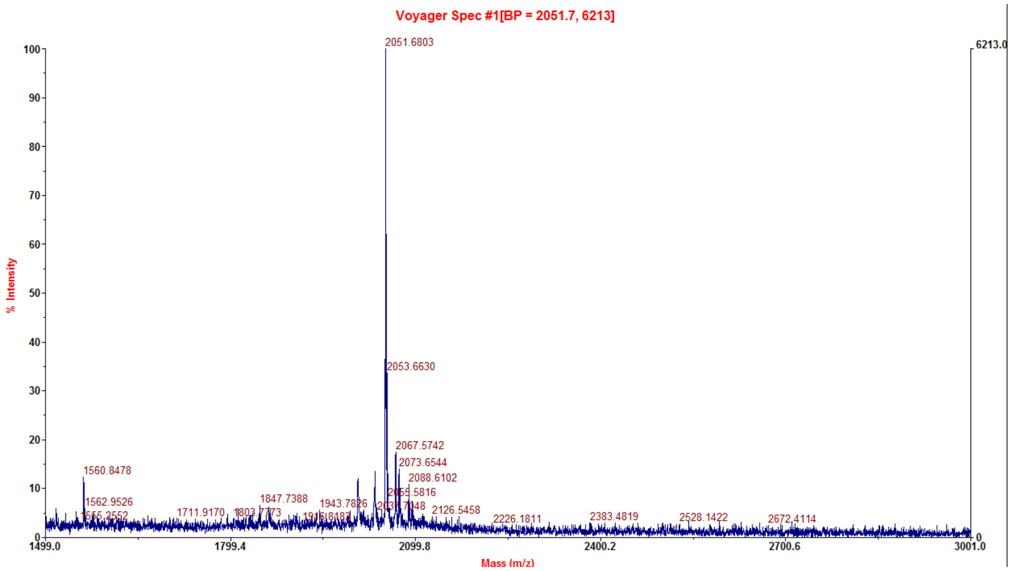

Expected [M + H]$^+$ = 2051.98 Da   Observed [M + H]$^+$ = 2051.68 Da

(c) APP$^{665-680}$(NL), **5**

EISEV**NL**DAEFRHDSG

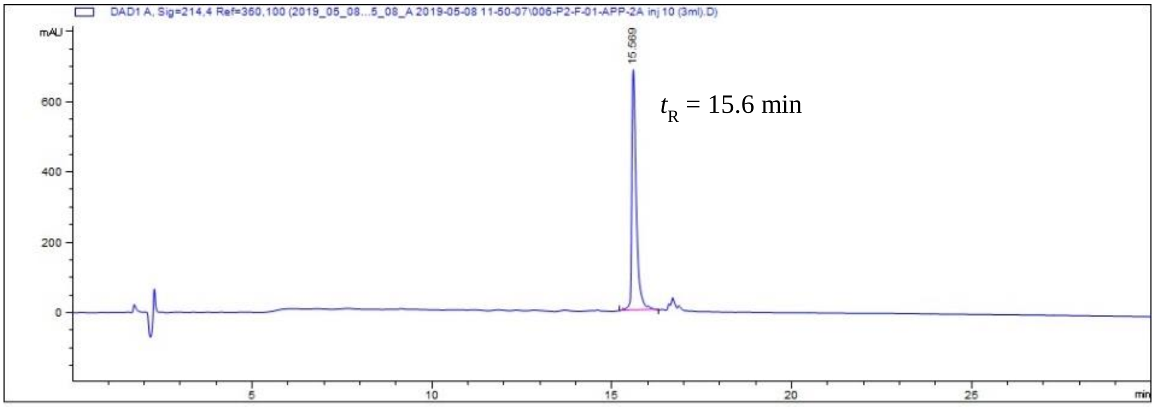

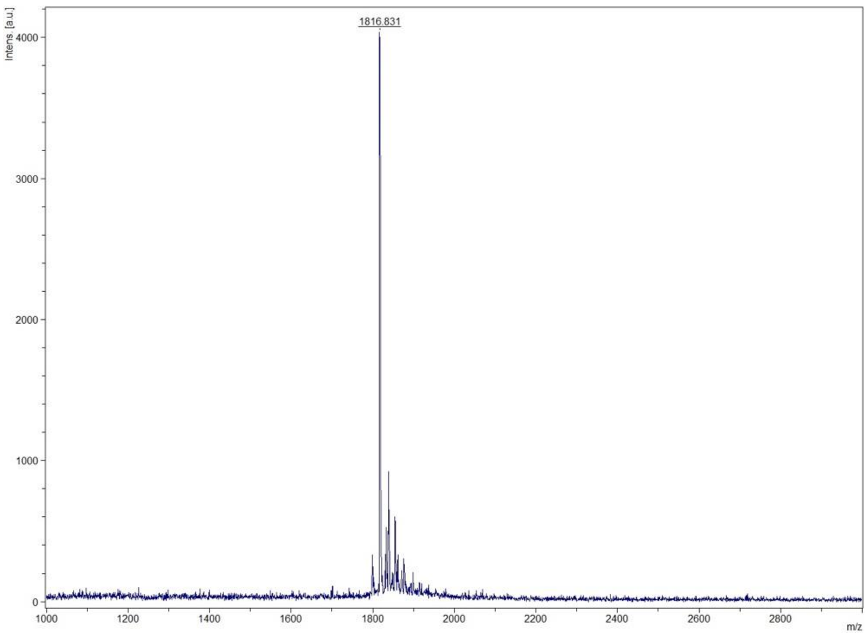

Expected $[M + H]^+$ = 1816.88 Da   Observed $[M + H]^+$ = 1816.83 Da

(d) APP$^{665\text{-}680}$(NL)-S*, **6**

EI**S***EV**NL**DAEFRHDSG

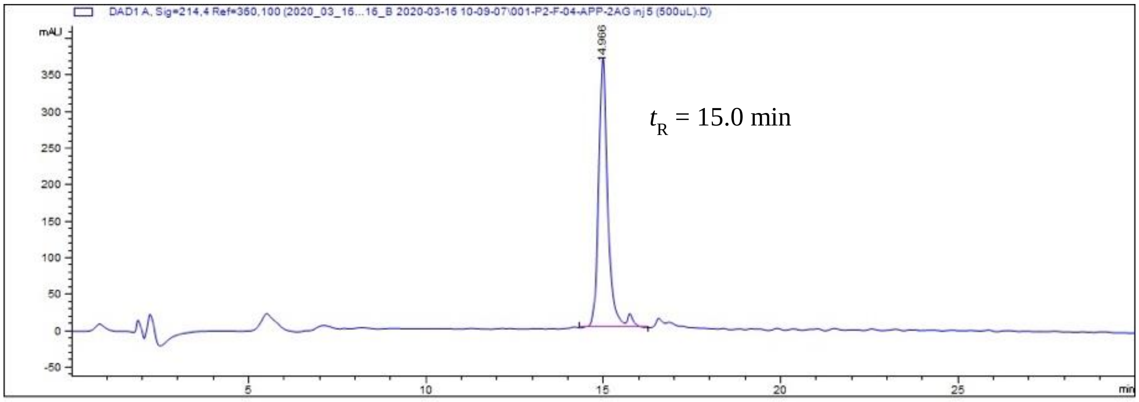

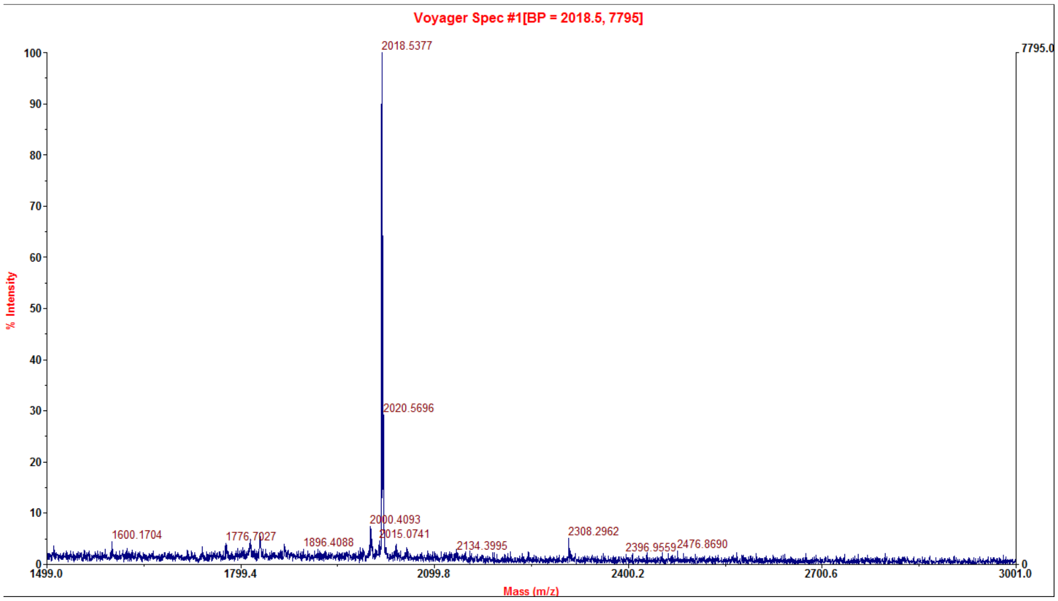

Expected [M + H]$^+$ = 2019.88 Da   Observed [M + H]$^+$ = 2018.54 Da

(e) APP$^{661\text{-}680}$, **7**

IKTEEISEVKMDAEFRHDSG

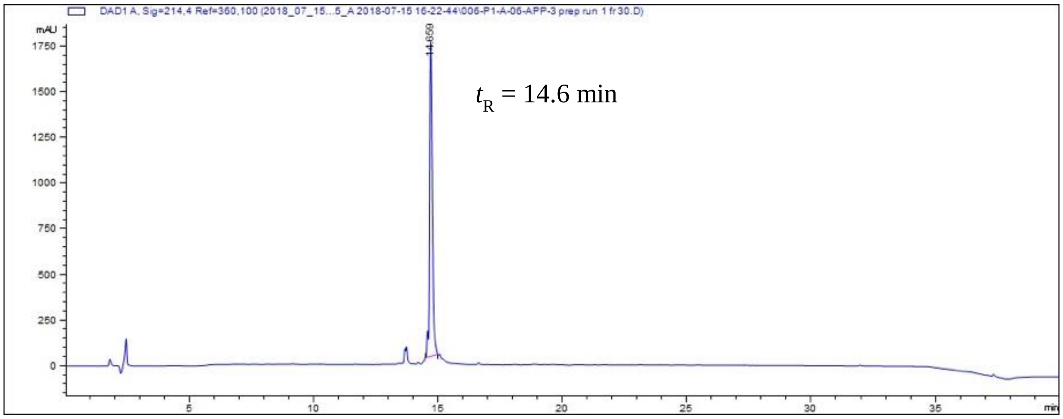

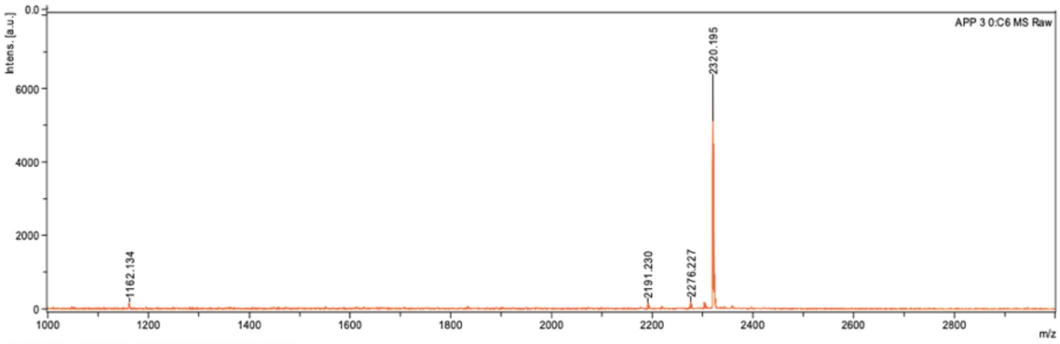

Expected $[M + H]^+$ = 2320.54 Da   Observed $[M + H]^+$ = 2320.19 Da

(f) APP$^{661\text{-}680}$-T*, **8**

IK**T***EEISEVKMDAEFRHDSG

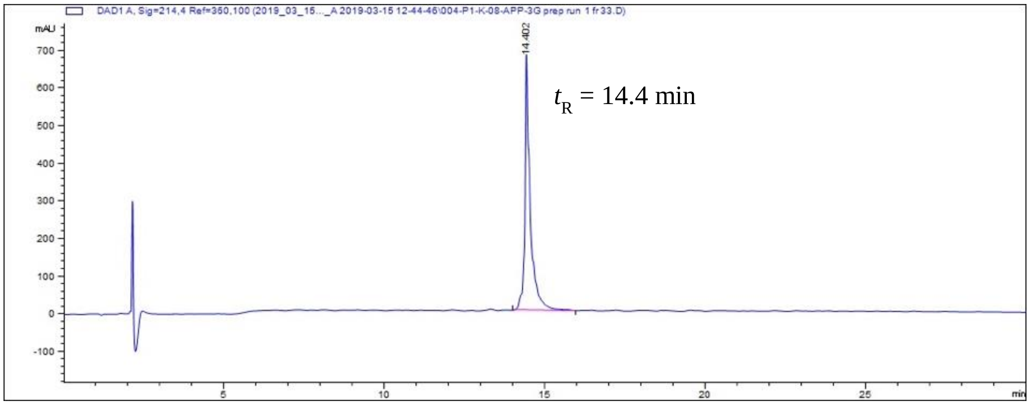

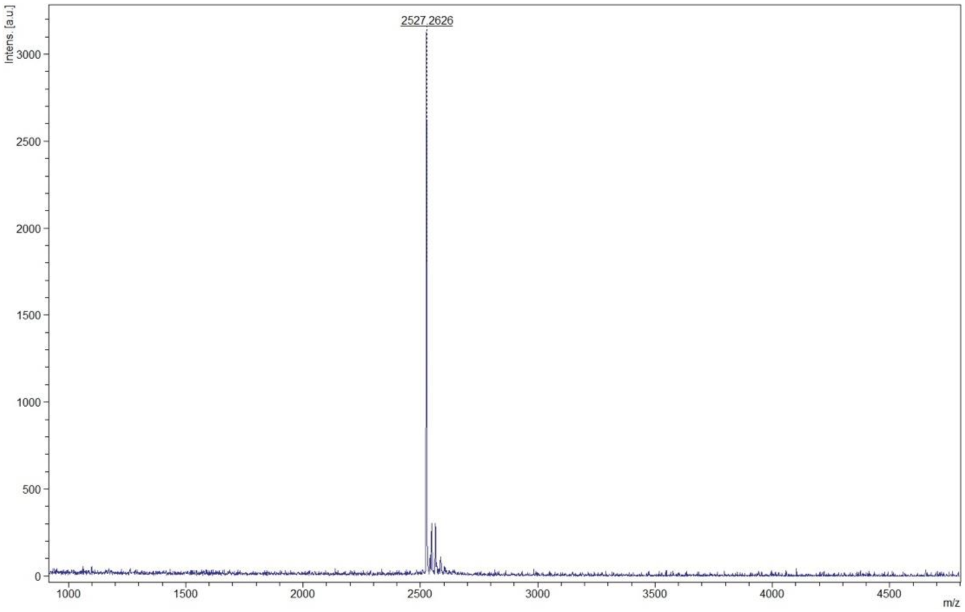

Expected $[M + H]^+$ = 2523.54 Da   Observed $[M + H]^+$ = 2527.26 Da

(g) APP$^{661-680}$(NL), **10**

IKTEEISEV**NL**DAEFRHDSG

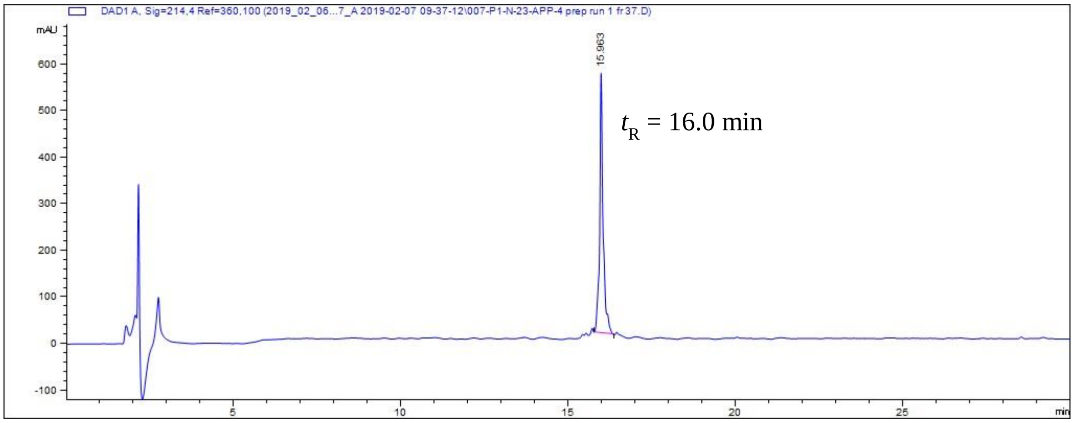

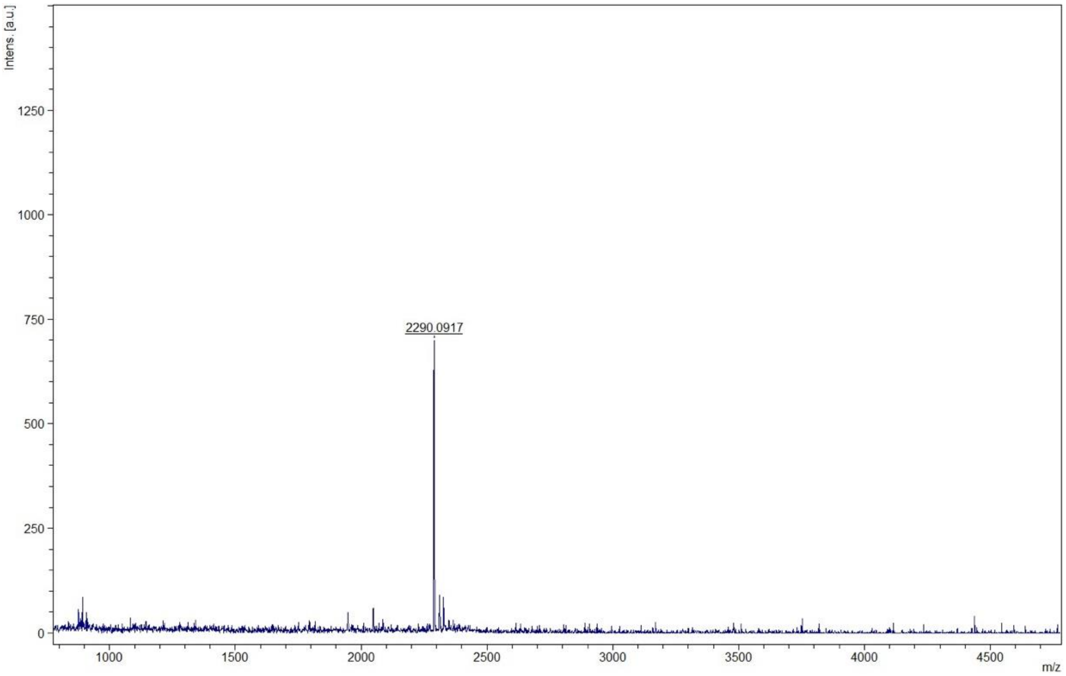

Expected [M + H]$^+$ = 2288.43 Da   Observed [M + H]$^+$ = 2290.09 Da

(h) APP$^{661\text{-}680}$(NL)-T*, **11**

IK**T***EEISEV**NL**DAEFRHDSG

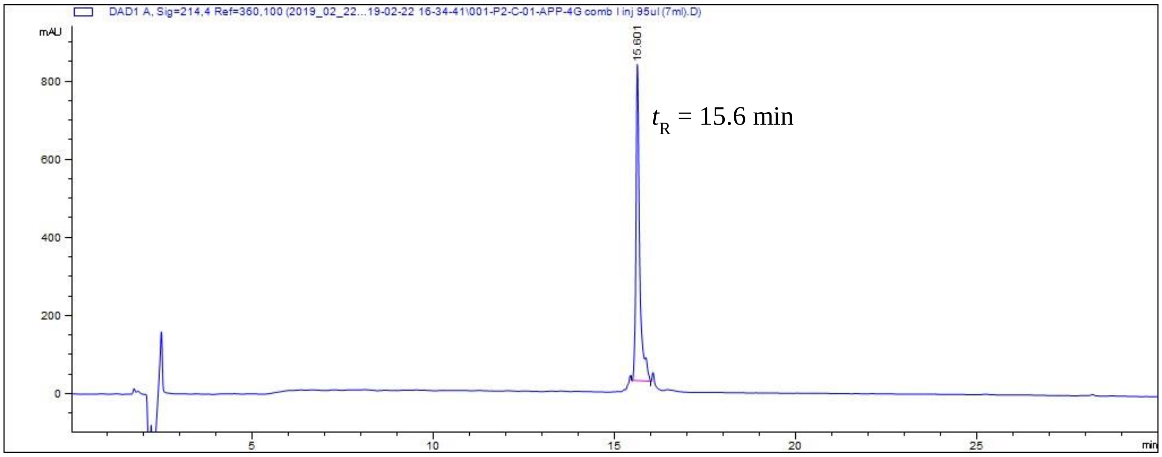

h) APP$^{661\text{-}680}$(NL)-T*, **11**
RP-HPLC gradient on Vydac Denali C18 column (250 x 4.6 mm, 5 μm, 120Å):
Eluents were 0.1% TFA in water (A) and 0.1% TFA in acetonitrile (B). The elution gradient was 0-60%B in 30 minutes with a flow rate of 1 mL/min. Detection was at λ = 214 nm.

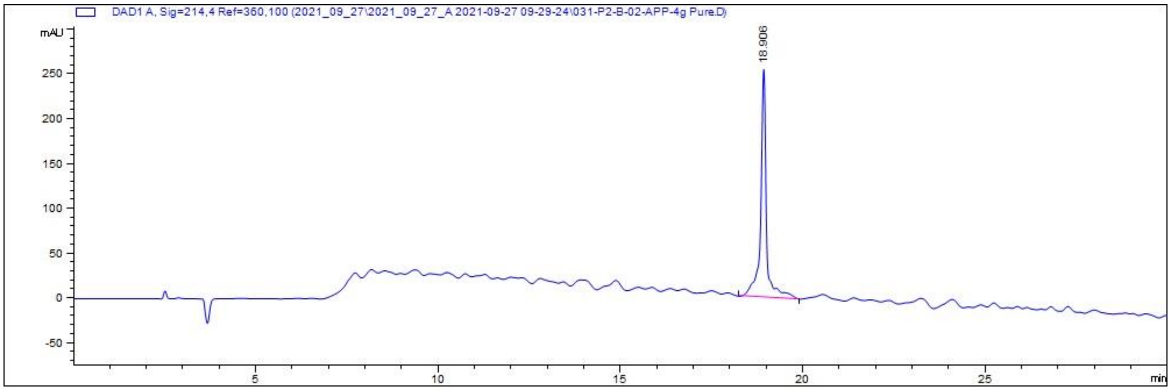

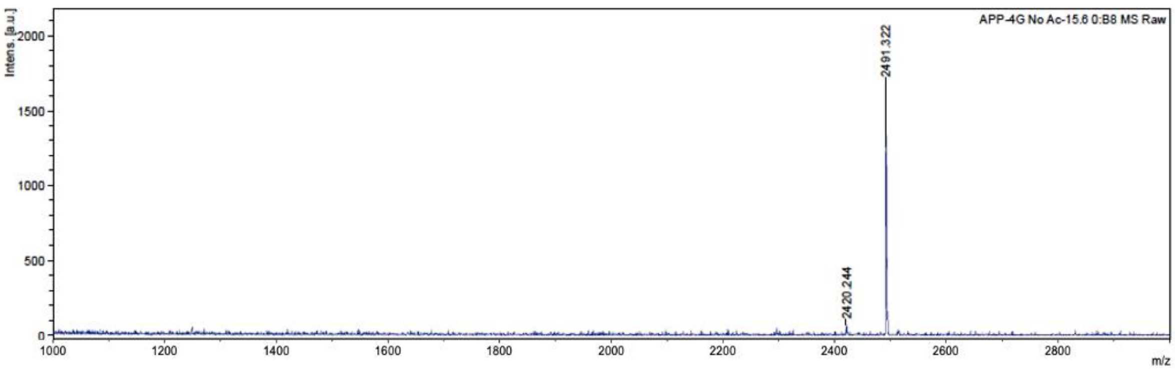

Expected $[M + H]^+$ = 2491.43 Da   Observed $[M + H]^+$ = 2491.32 Da

RP-HPLC gradient on Vydac Denali C18 column (250 x 4.6 mm, 5 μm, 120Å) for **9** and **12**: Eluents were 0.1% TFA in water (A) and 0.1% TFA in acetonitrile (B). The elution gradient was 0-60%B in 30 minutes with a flow rate of 1 mL/min. Detection was at λ = 214 nm.

(i) APP$^{661\text{-}680}$-T*, S*, **9**

IK**T***EEI**S***EVKMDAEFRHDSG

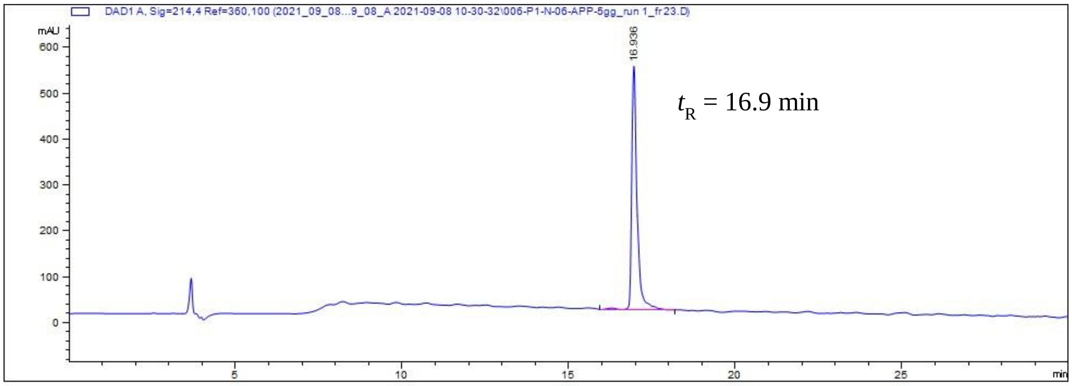

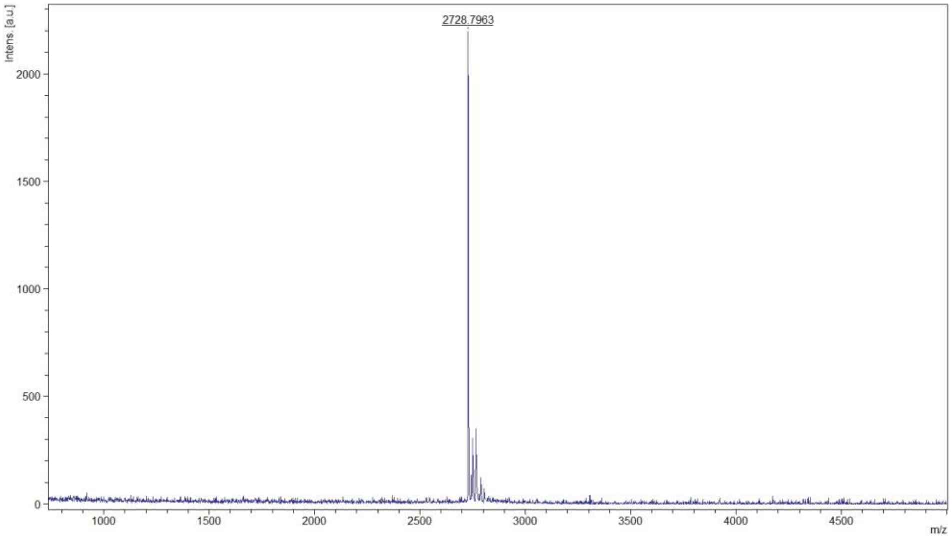

Expected $[M + H]^+$ = 2726.54 Da   Observed $[M + H]^+$ = 2728.80 Da

(j) APP$^{661\text{-}680}$(NL)-T*, S*, **12**

IK**T***EEI**S***EV**NL**DAEFRHDSG

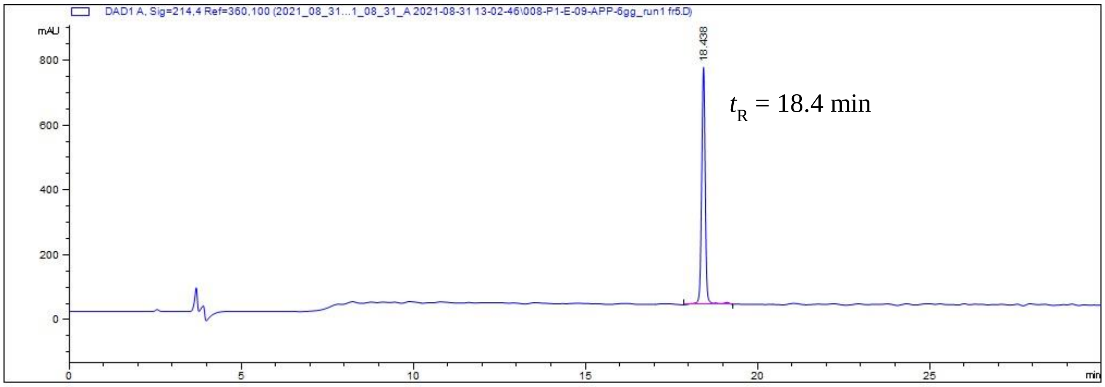

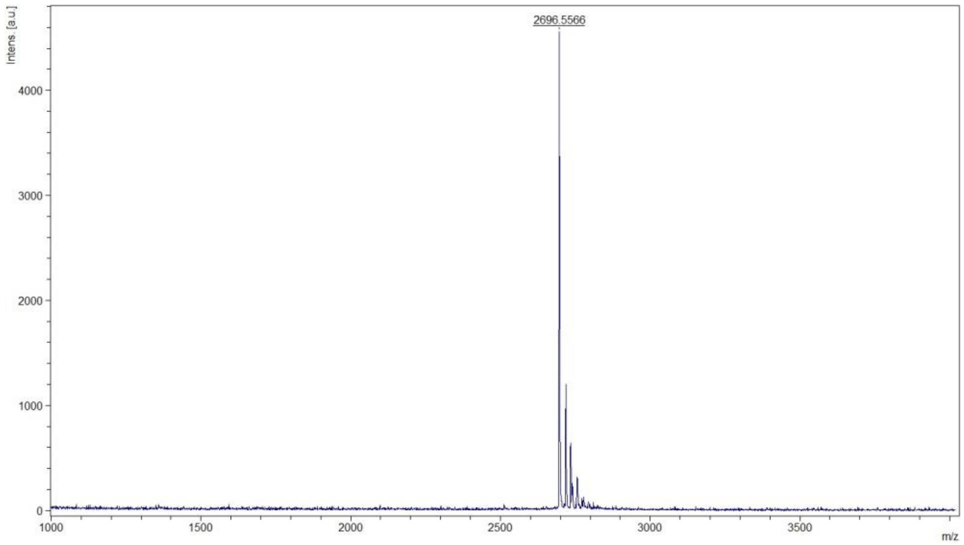

Expected $[M + H]^+$ = 2694.43 Da   Observed $[M + H]^+$ = 2696.55 Da

2. Secondary structure predictions from the CD spectra of **3-12** using the BeStSel method

I Nonglycosylated APP peptides (3, 5, 7, and 10)

(a) Water

APP$^{665\text{-}680}$ (**3**)

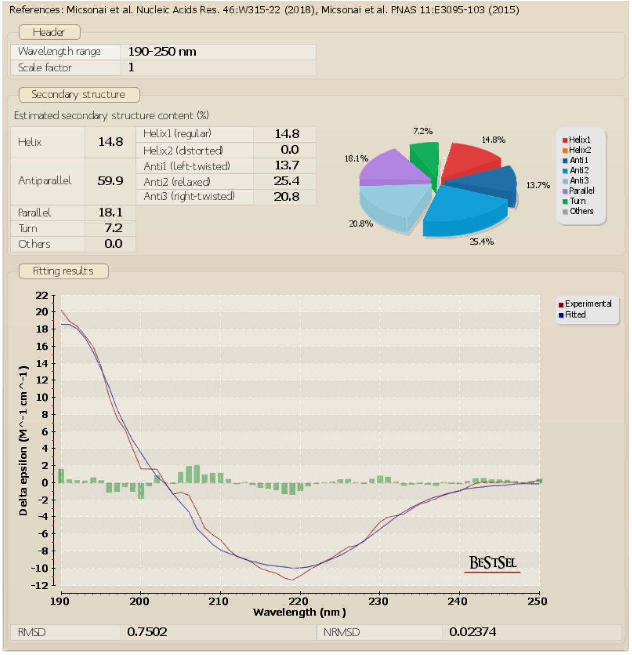

APP$^{665\text{-}680}$(NL) (**5**)

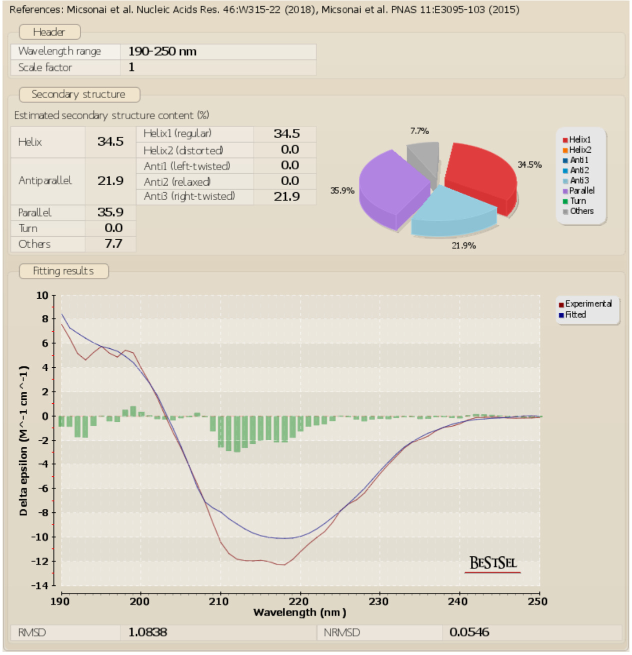

APP$^{661\text{-}680}$ (**7**)

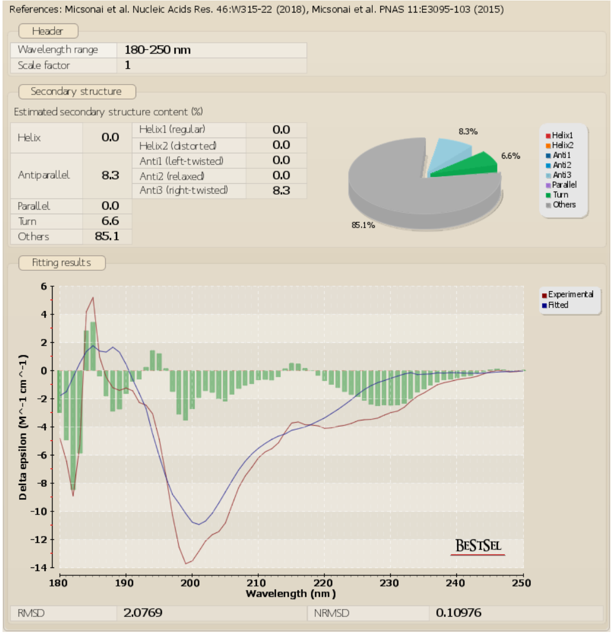

APP$^{661\text{-}680}$(NL) (**10**)

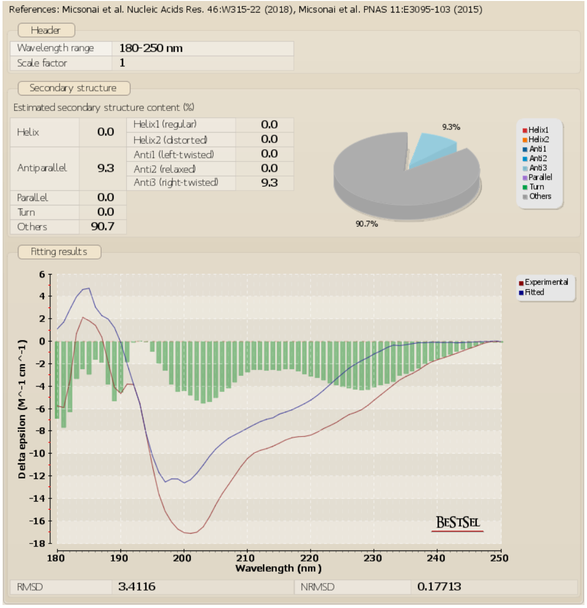

(b) 10 mM Sodium phosphate buffer, pH 7.4

APP$^{665\text{-}680}$ (**3**)

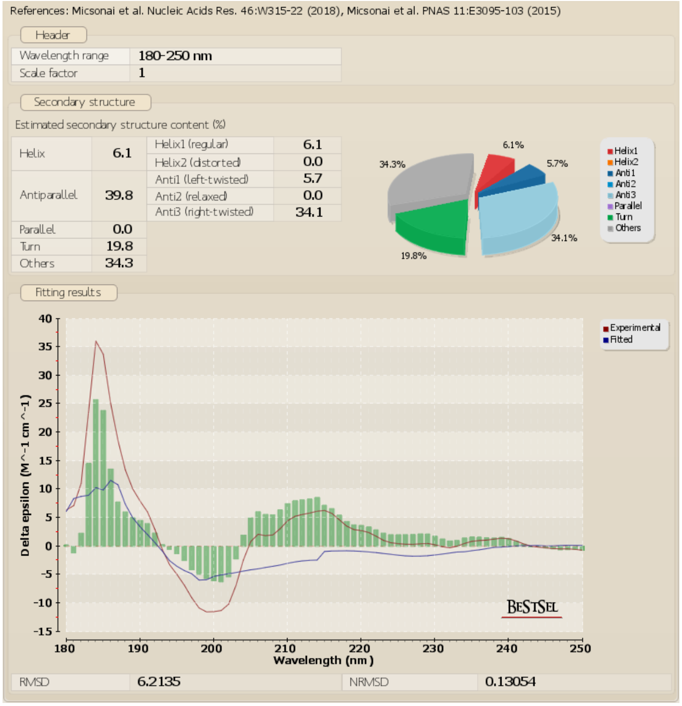

APP$^{665\text{-}680}$(NL) (**5**)

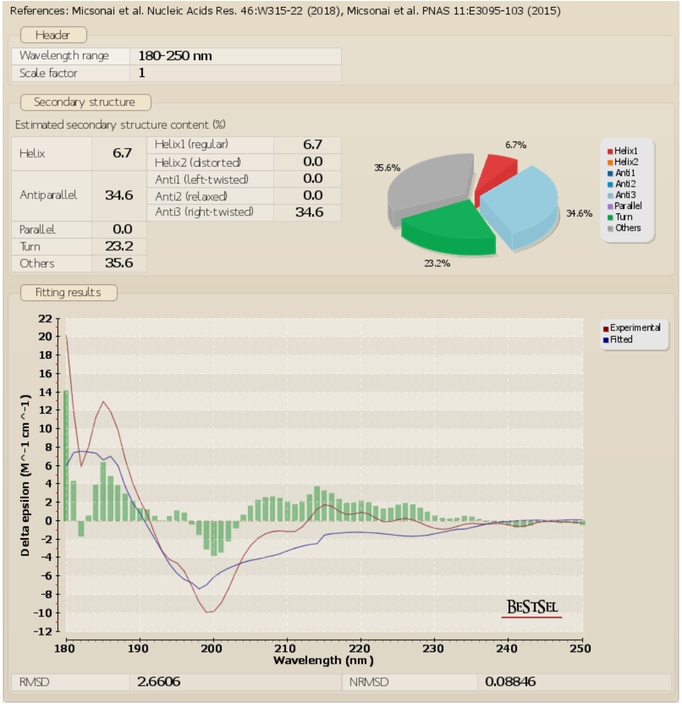

APP$^{661\text{-}680}$ (**7**)

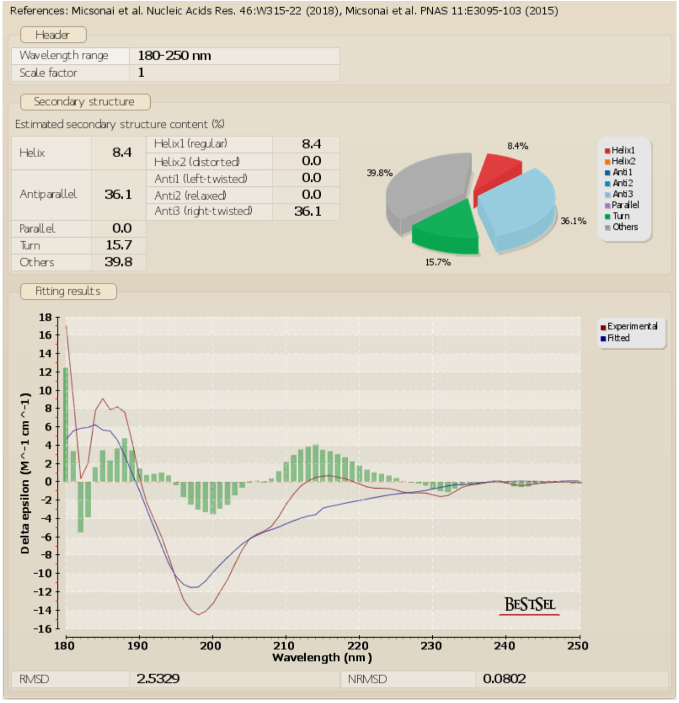

APP$^{661\text{-}680}$(NL) (**10**)

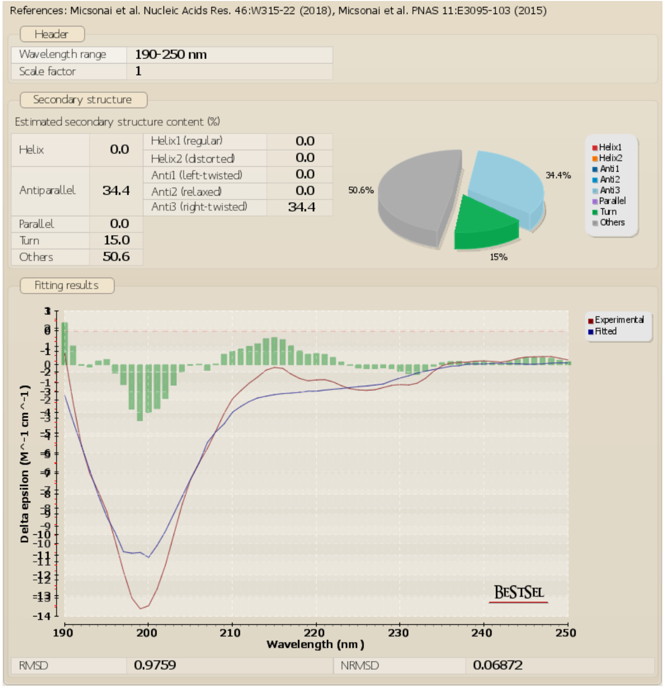

(c) TFE:water = 1:1 (v/v)

APP$^{665-680}$ (**3**)

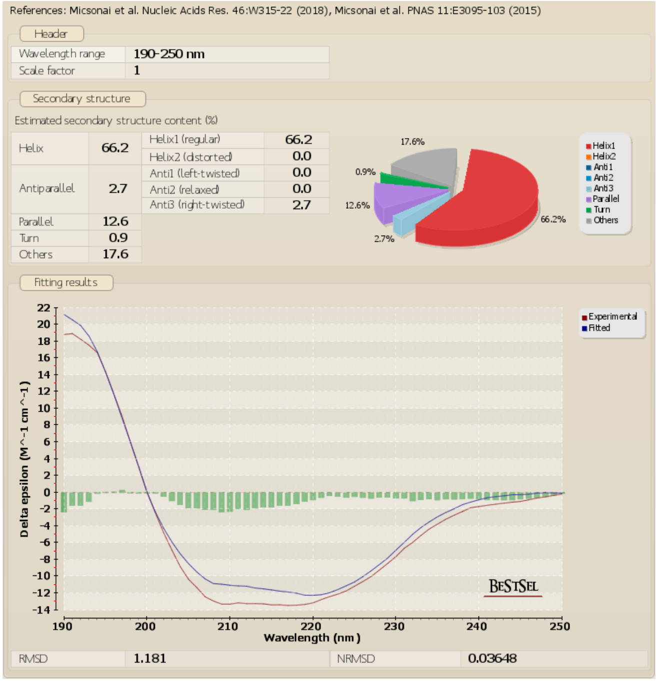

APP$^{665-680}$(NL) (**5**)

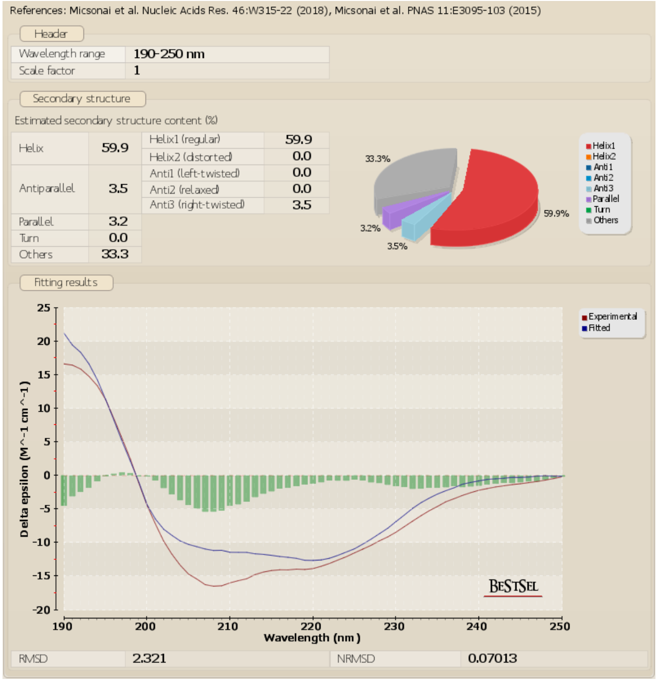

APP$^{661-680}$ (**7**)

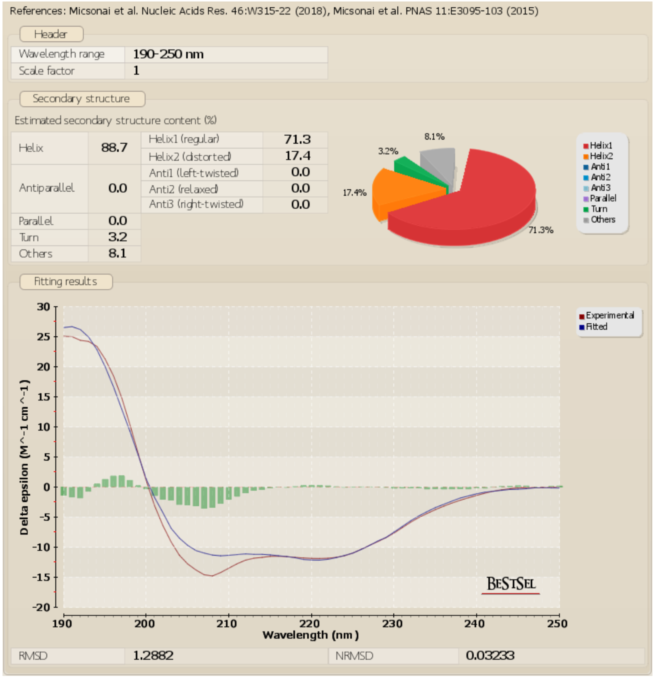

APP$^{661-680}$(NL) (**10**)

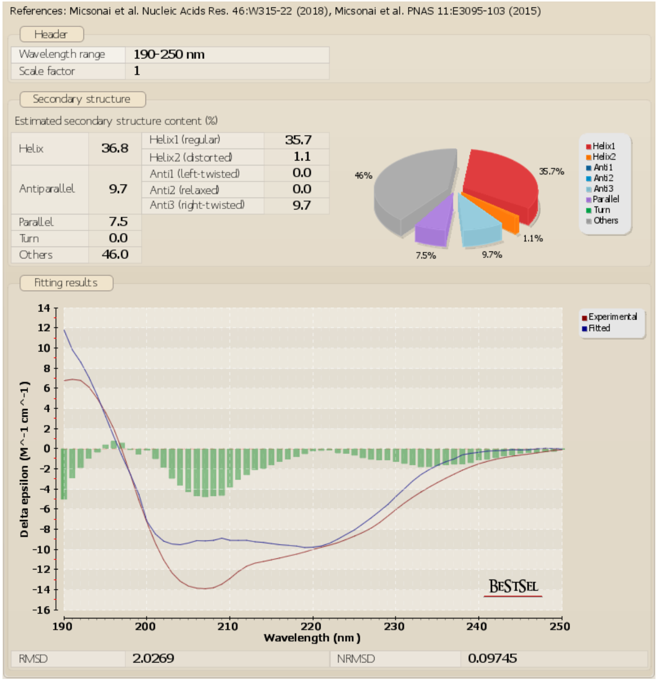

II APP glycopeptides (4, 6, 8, 9, 11, and 12)

(a) Water

APP$^{665\text{-}680}$-S* (**4**)

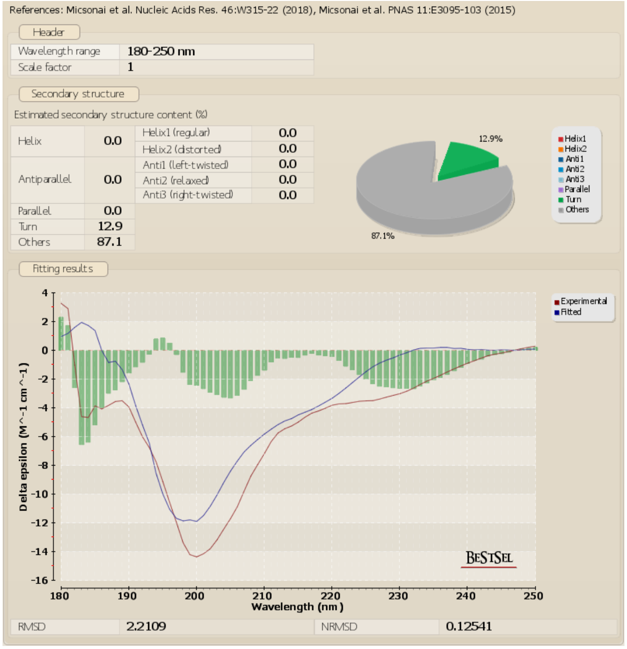

APP$^{665\text{-}680}$(NL)-S* (**6**)

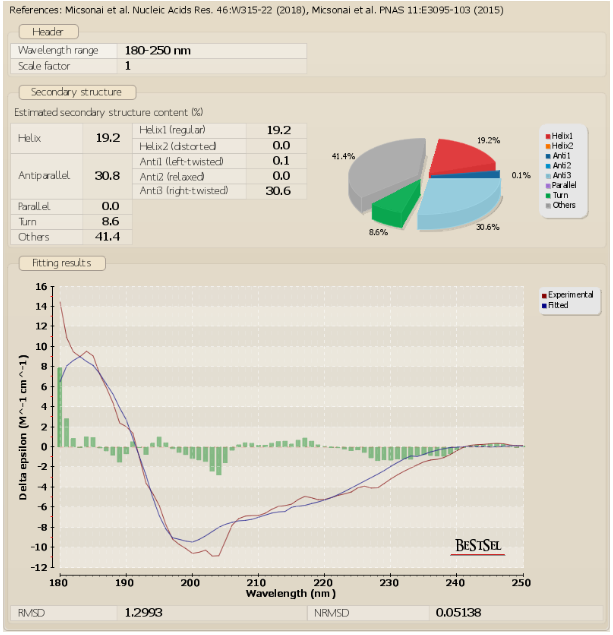

APP$^{661\text{-}680}$-T* (**8**)

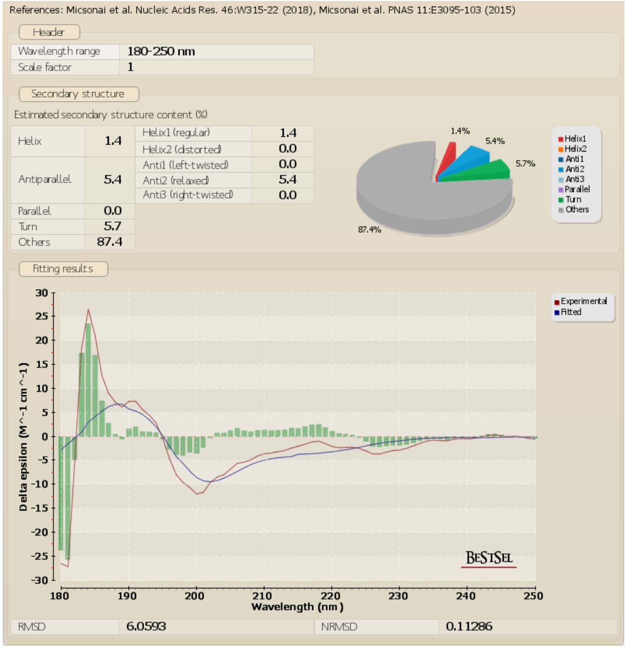

APP$^{661\text{-}680}$-T*, S* (**9**)

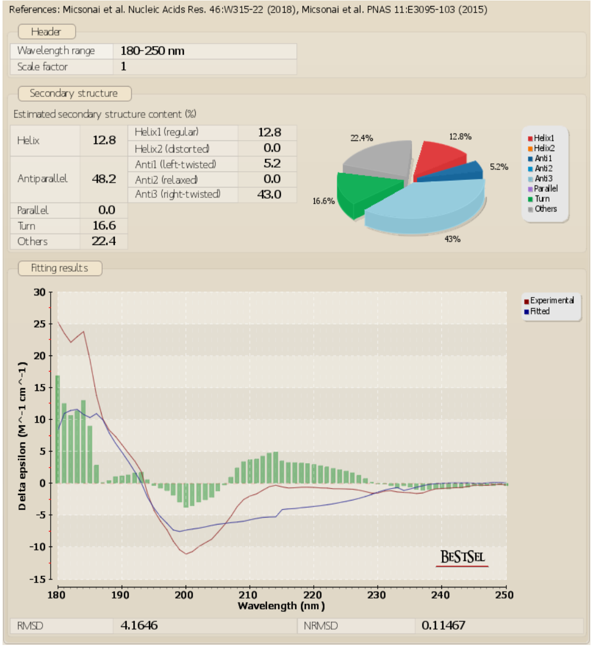

APP$^{661\text{-}680}$(NL)-T* (**11**)

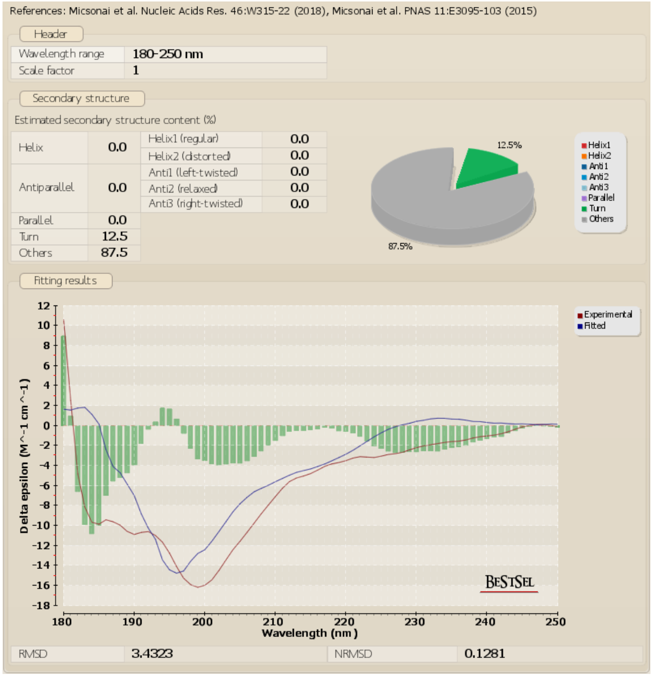

APP$^{661\text{-}680}$(NL)-T*, S* (**12**)

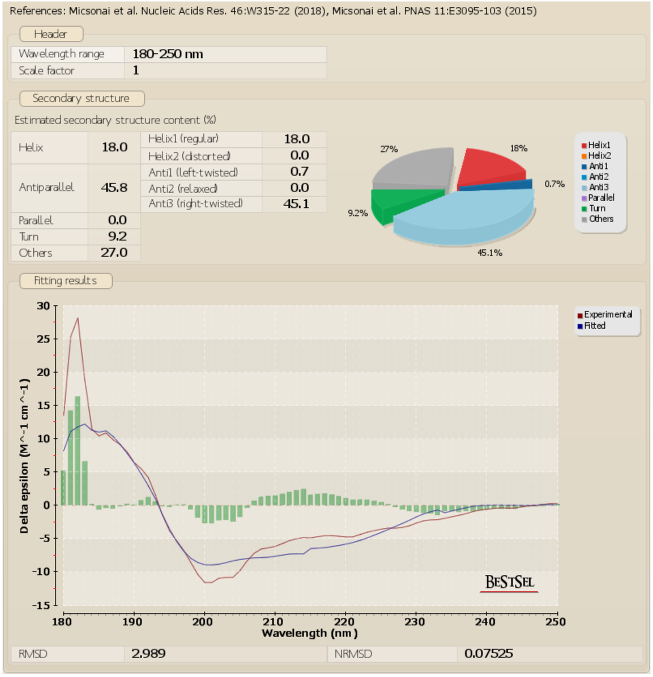

(b) 10 mM Sodium phosphate buffer, pH 7.4

APP$^{665\text{-}680}$-S* (**4**)

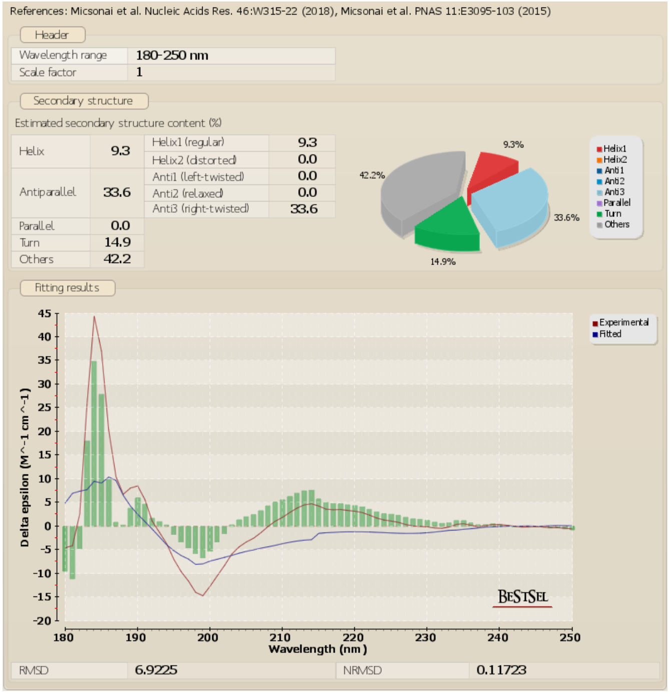

APP$^{665\text{-}680}$(NL)-S* (**6**)

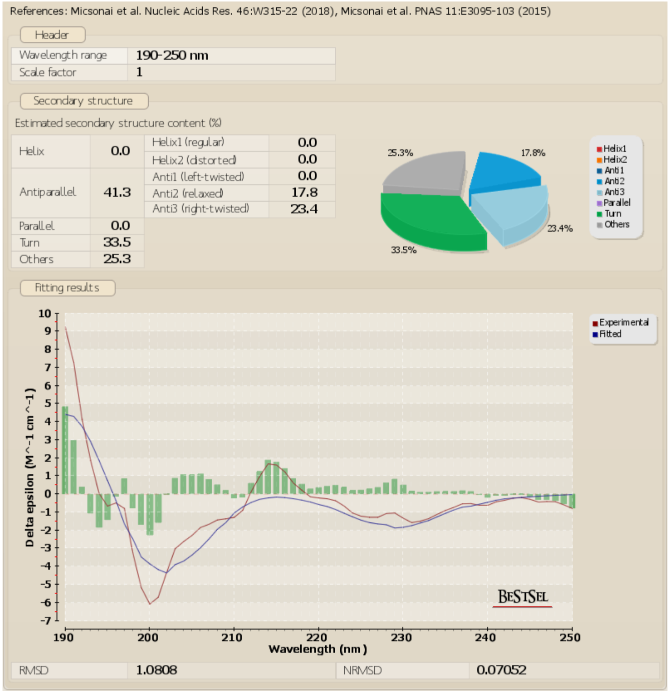

APP$^{661\text{-}680}$-T* (**8**)

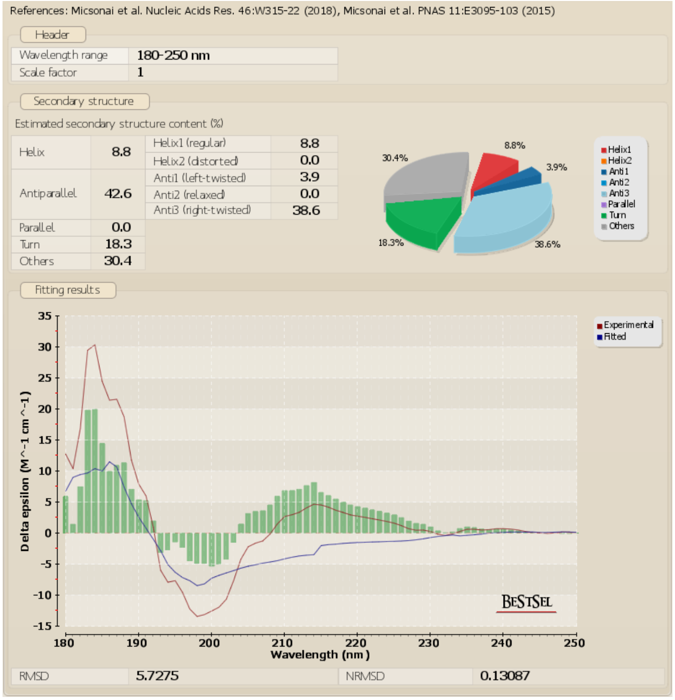

APP$^{661\text{-}680}$-T*, S* (**9**)

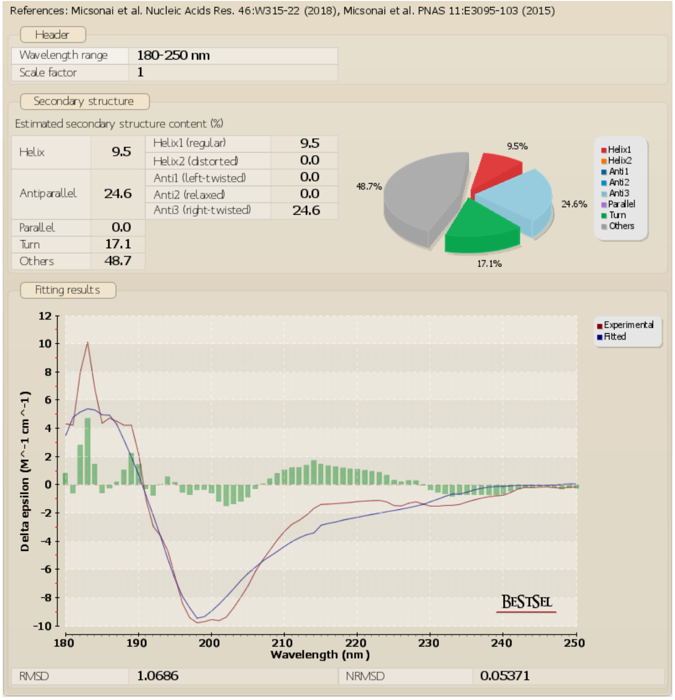

APP$^{661\text{-}680}$(NL)-T* (**11**)

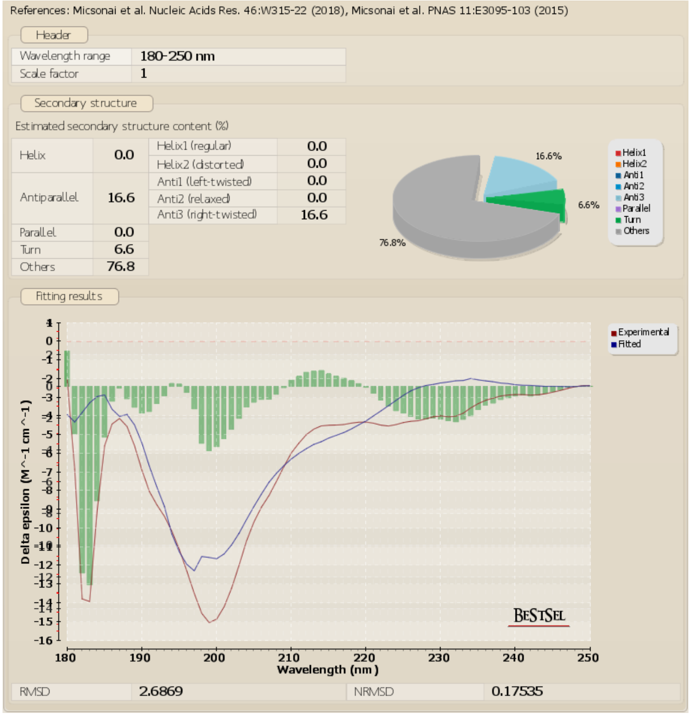

APP$^{661\text{-}680}$(NL)-T*, S* (**12**)

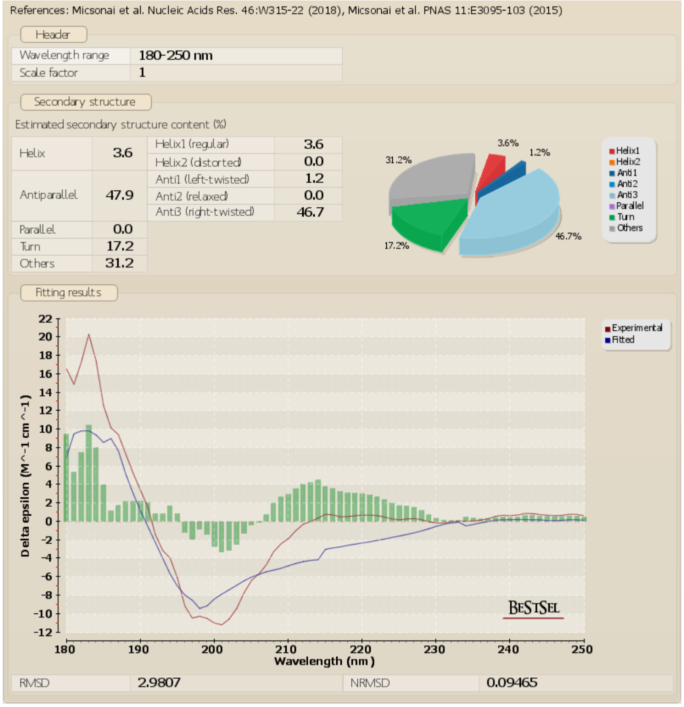

(c) TFE:water = 1:1 (v/v)

APP$^{665\text{-}680}$-S* (**4**)

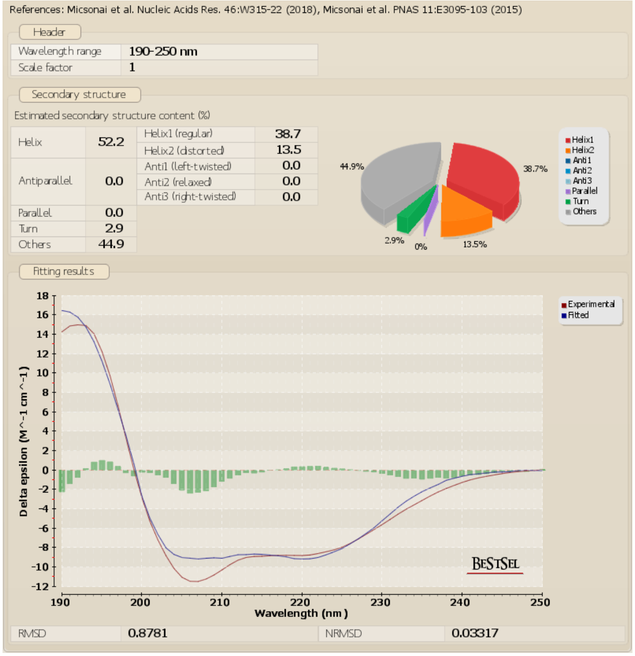

APP$^{665\text{-}680}$(NL)-S* (**6**)

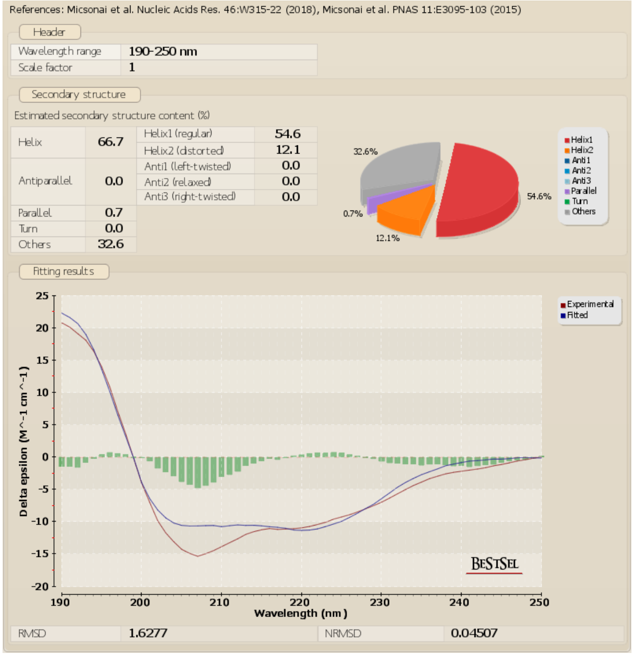

APP$^{661\text{-}680}$-T* (**8**)

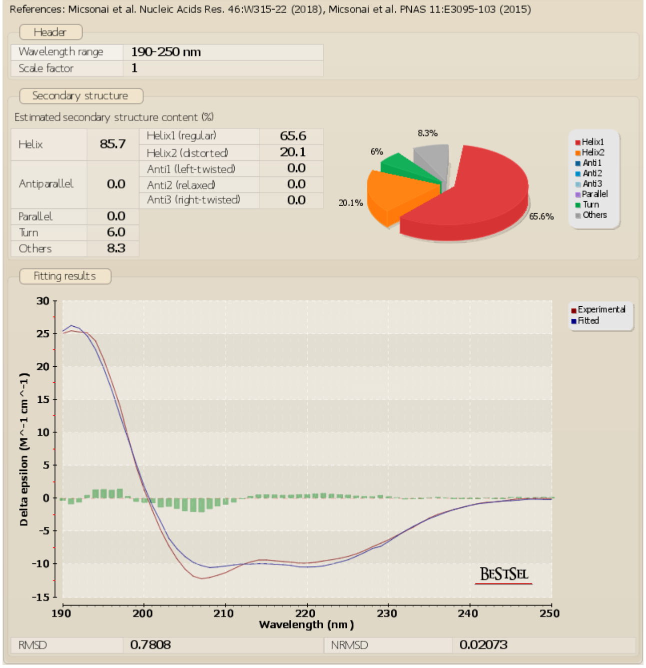

APP$^{661\text{-}680}$-T*, S* (**9**)

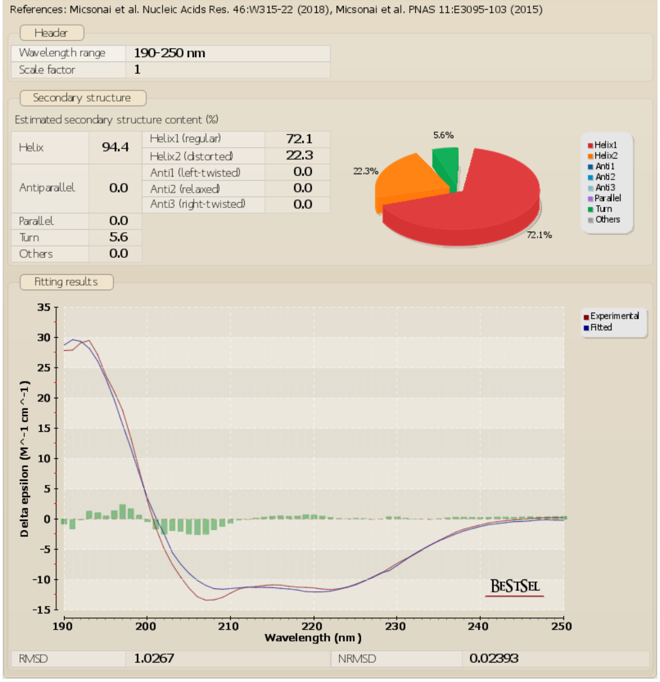

APP$^{661-680}$(NL)-T* (**11**)

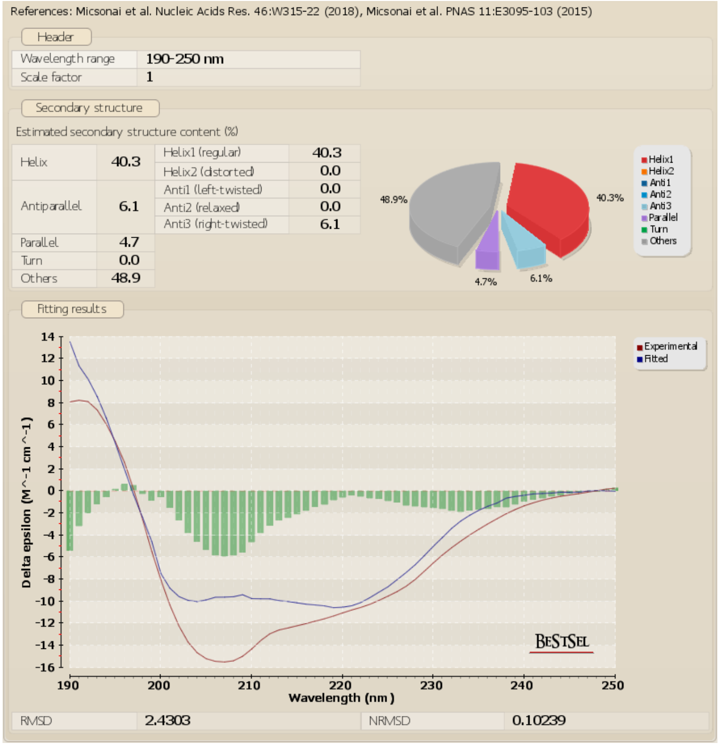

APP$^{661-680}$(NL)-T*, S* (**12**)

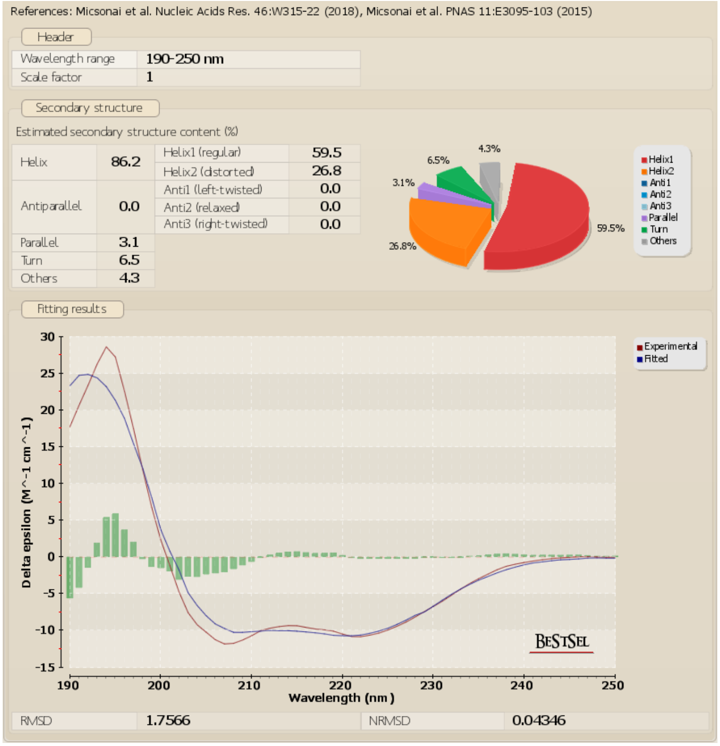

3. Enzyme cleavage assays with APP (glyco)peptides **3-12** by RP-HPLC and MALDI-TOF MS analysis

For the BACE1 enzyme cleavage assay, APP-based (glyco)peptide substrates were diluted in BACE1 activity buffer and incubated with BACE1 for 24 h at 37 °C in the dark. After the incubation period, the enzyme cleavage solutions containing APP-based (glyco)peptide substrates and BACE1 were analyzed using analytical RP-HPLC.

RP-HPLC gradient on Phenomenex Aeris Peptide C18 column (150 x 4.6 mm, 3.6 μm, 100Å): Eluents were 0.1% TFA in water (A) and 0.1% TFA in acetonitrile (B). The elution gradient was 0-60%B in 30 minutes with a flow rate of 0.8 mL/min. Detection was at λ = 214 nm.

S*/**T***= Ser(-α-GalNAc) **1**/Thr(-α-GalNAc) **2**

(a) APP$^{665\text{-}680}$, **3** in activity buffer

EISEVKM~DAEFRHDSG

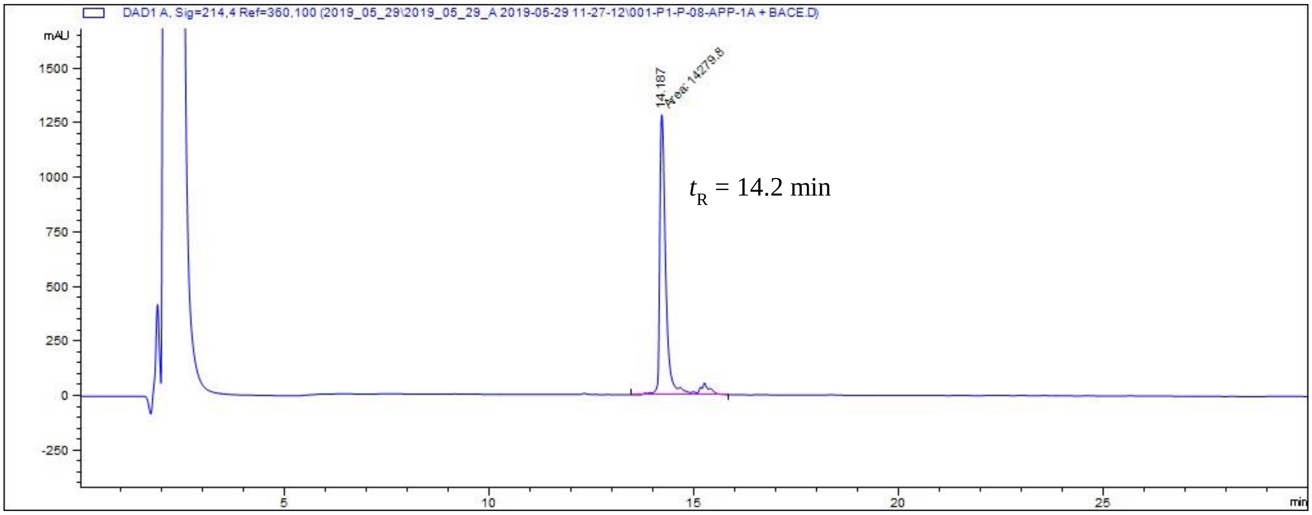

(b) APP$^{665\text{-}680}$ (**3**) + BACE1 in activity buffer

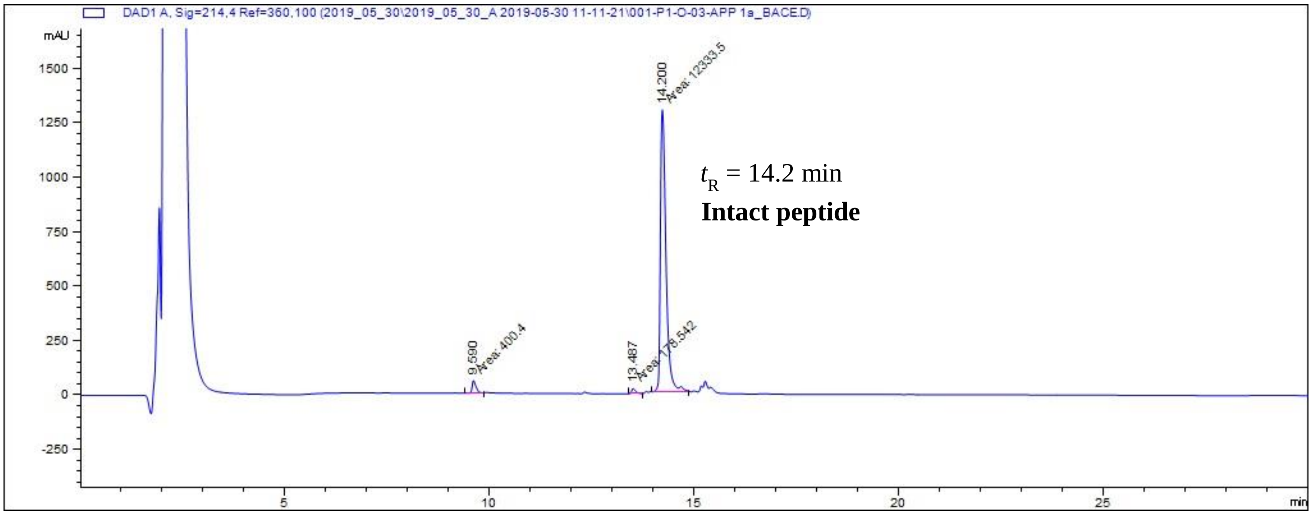

(c) APP$^{665\text{-}680}$-S*, **4** in activity buffer

EI**S***EVKM~DAEFRHDSG

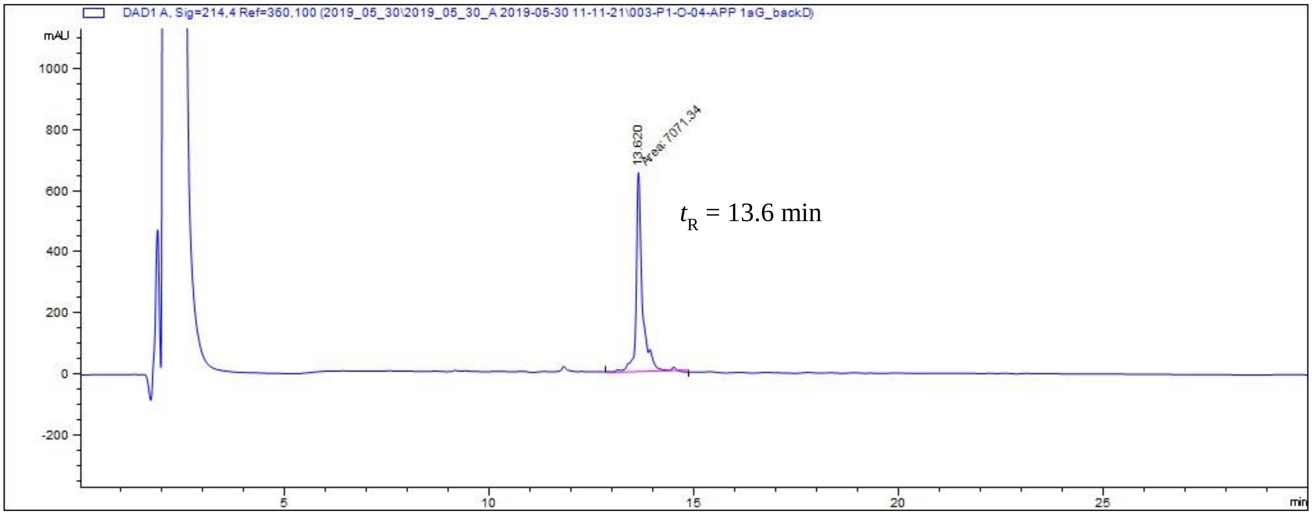

(d) APP$^{665\text{-}680}$-S* (**4**) + BACE1 in activity buffer

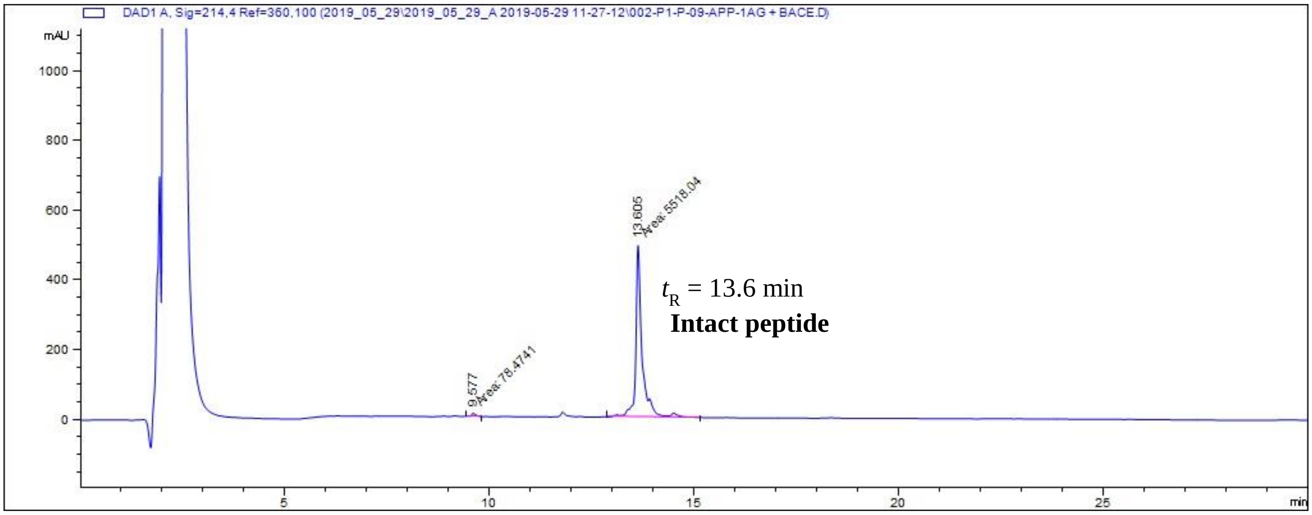

(e) APP$^{665\text{-}680}$(NL), **5** in activity buffer

EISEV**NL**~DAEFRHDSG

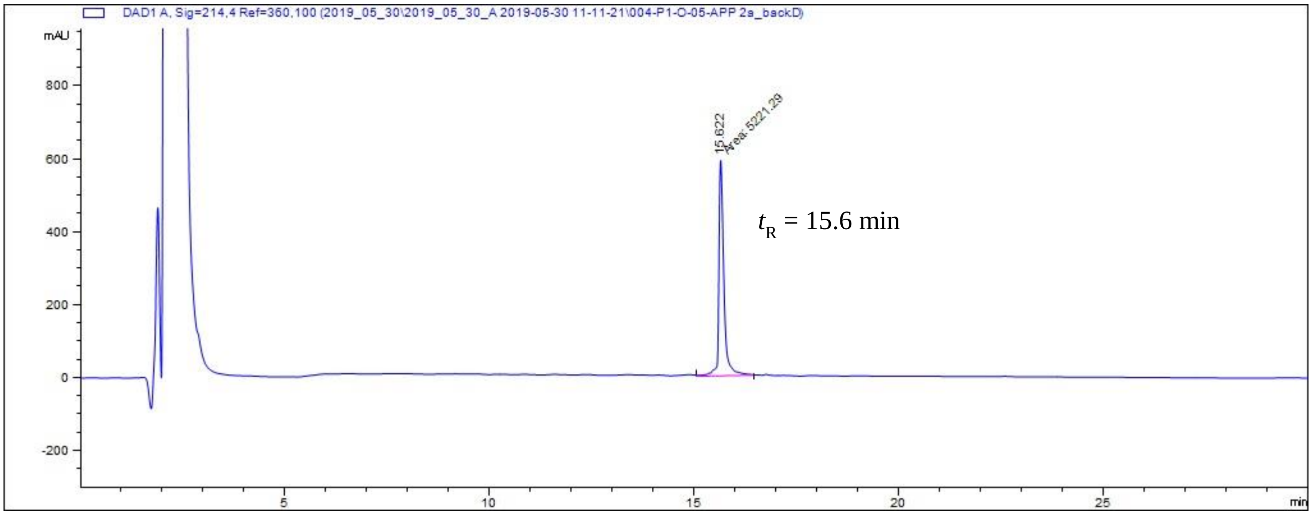

(f) APP$^{665\text{-}680}$(NL) (**5**) + BACE1 in activity buffer

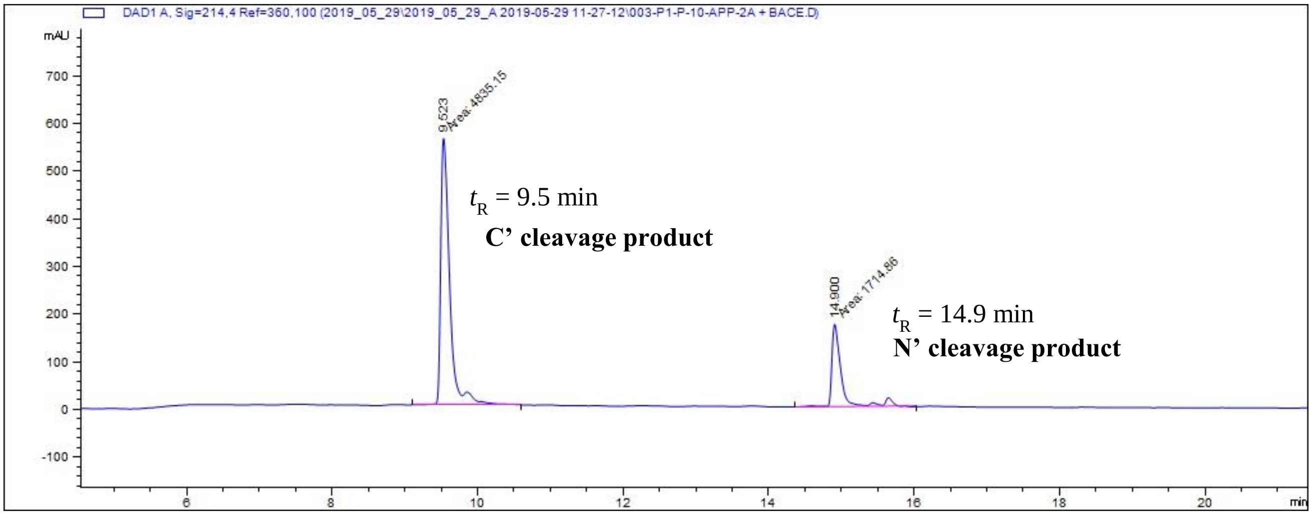

MALDI-TOF MS of BACE1 cleaved fragments of peptide **5**

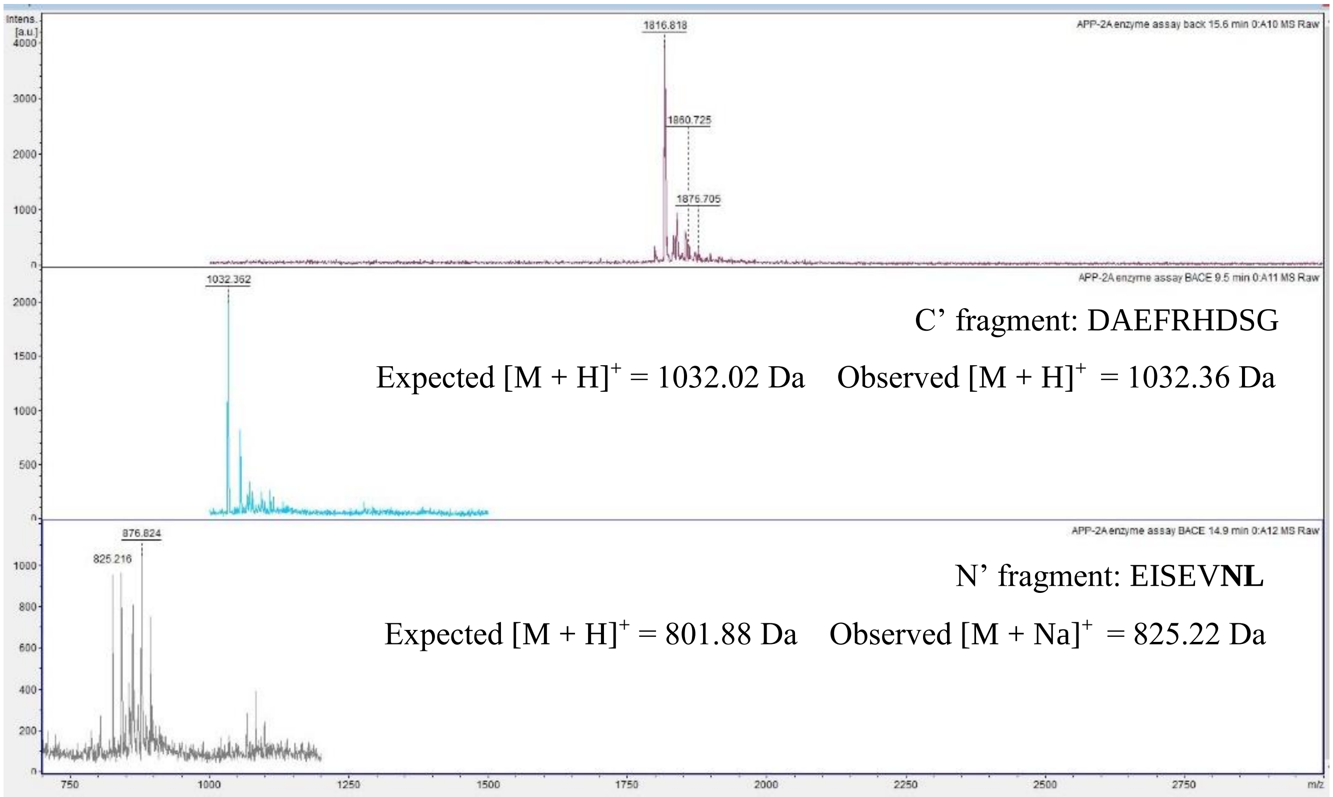

(g) APP$^{665\text{-}680}$(NL)-S*, **6** in activity buffer

EI**S***EV**NL**~DAEFRHDSG

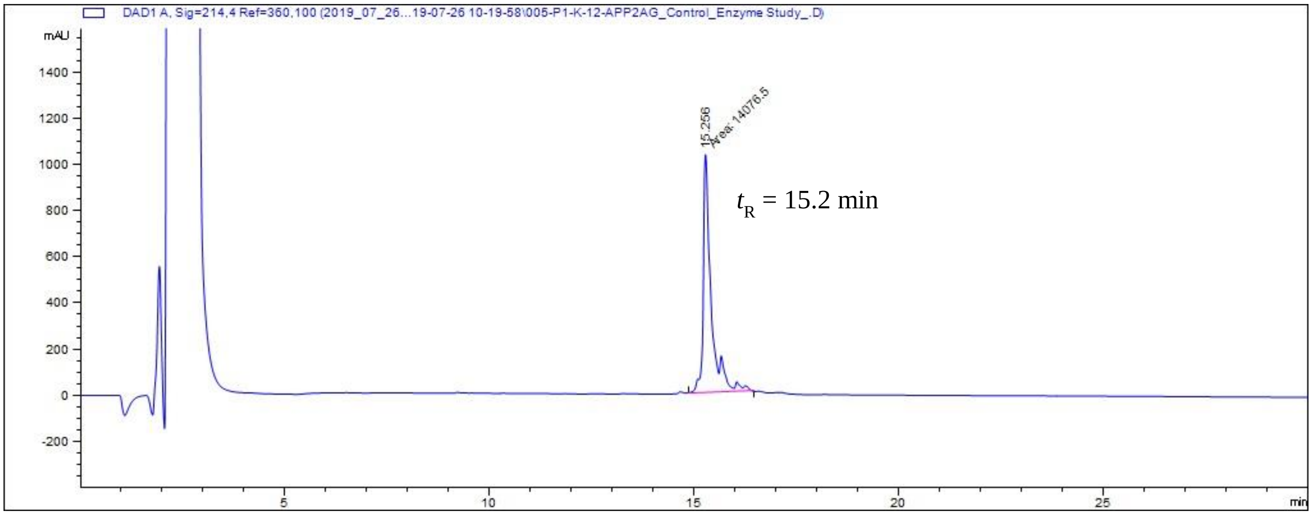

(h) $APP^{665\text{-}680}$(NL)-S* (**6**) + BACE1 in activity buffer

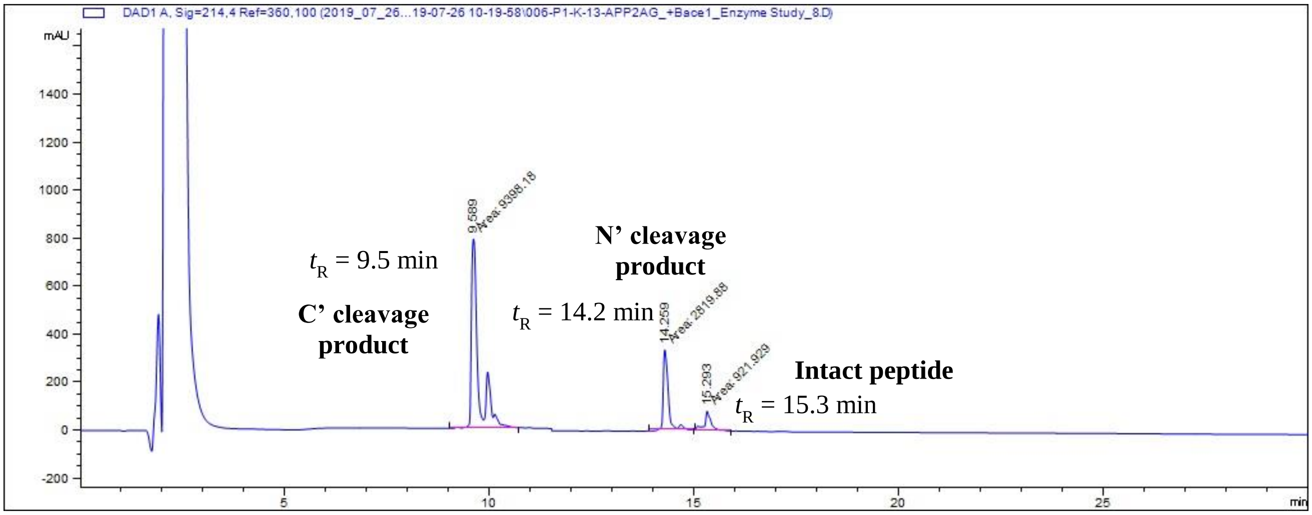

MALDI-TOF MS of BACE1 cleaved N' fragment of peptide **6**

N' fragment: EI**S***EV**NL**

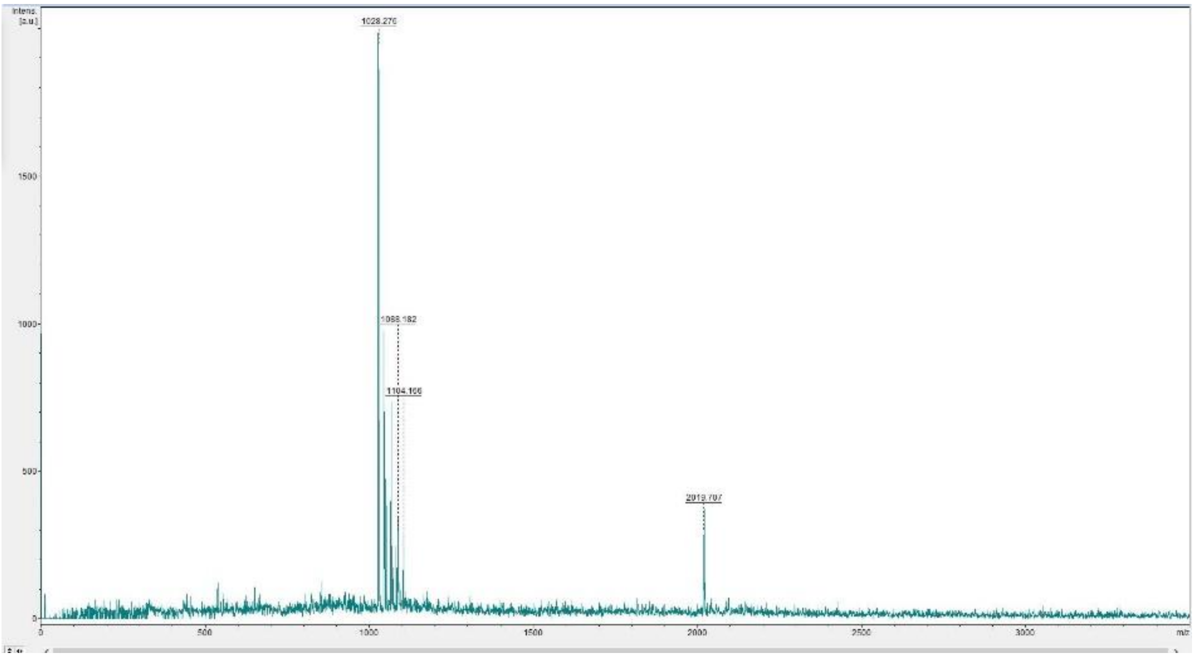

Expected $[M + H]^+$ = 1003.88 Da Observed $[M + Na]^+$ = 1028.28 Da

(i) APP$^{661\text{-}680}$, **7** in activity buffer

IKTEEISEVKM~DAEFRHDSG

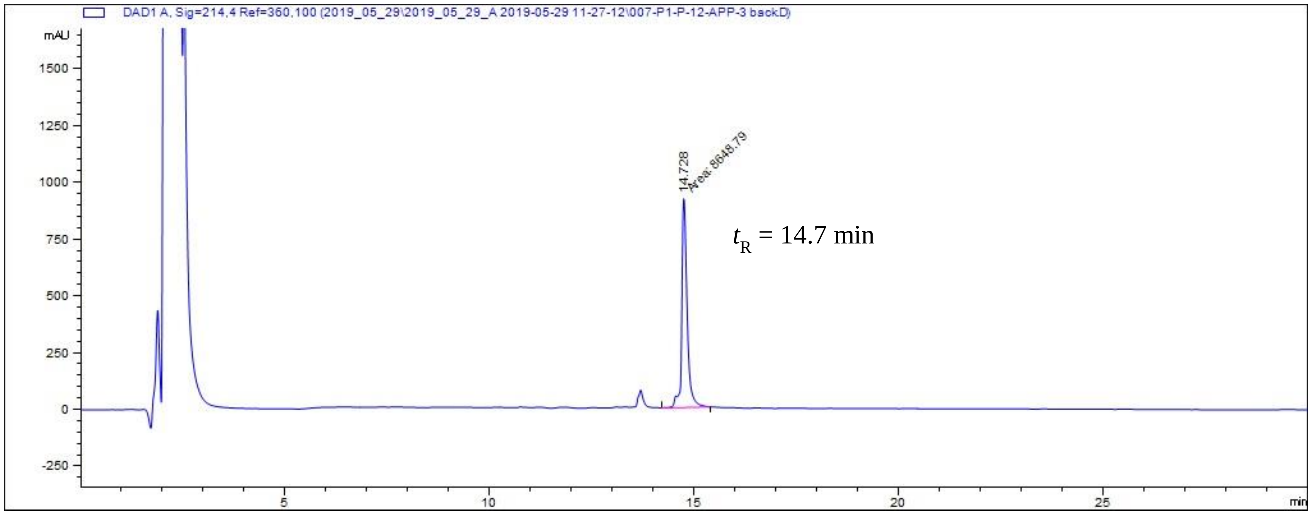

(j) APP$^{661\text{-}680}$ (**7**) + BACE1 in activity buffer

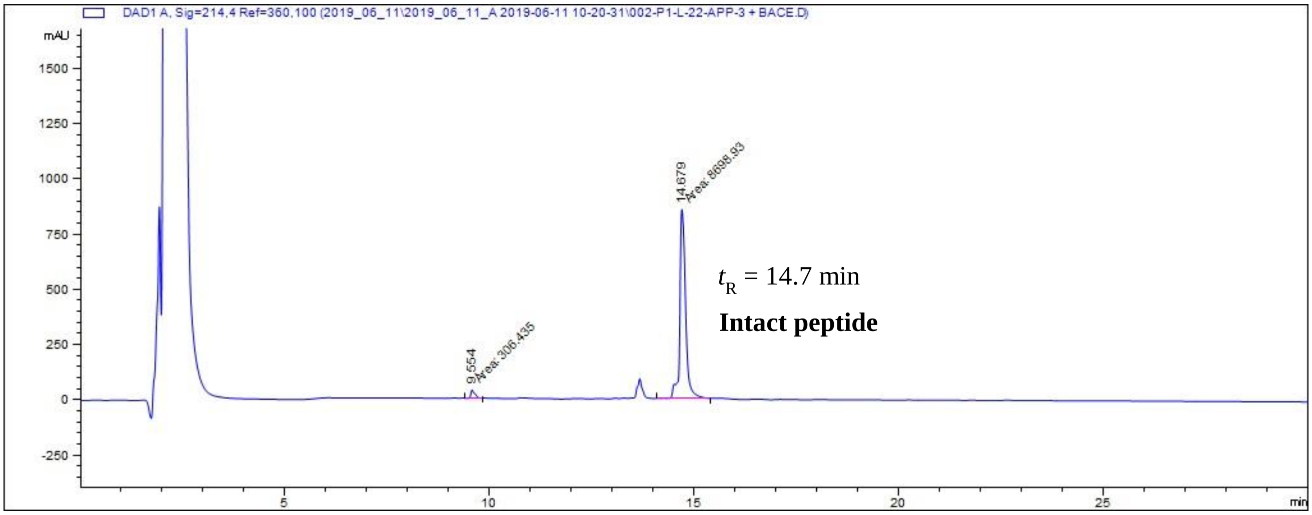

(k) APP$^{661-680}$-T*, **8** in activity buffer

IKTEEISEVKM~DAEFRHDSG

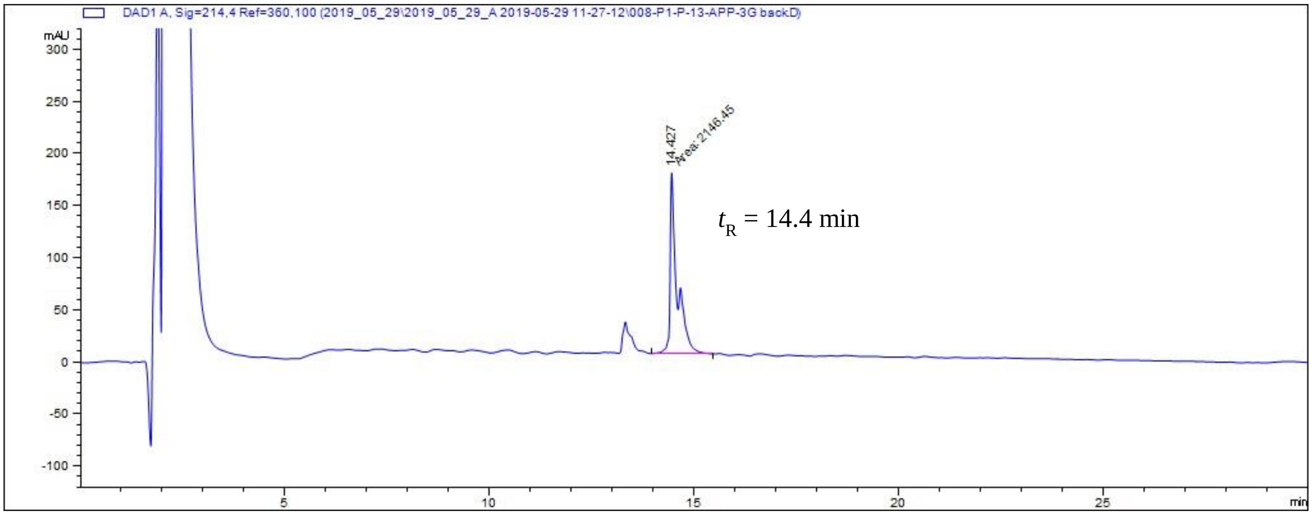

(l) APP$^{661-680}$-T* (**8**) + BACE1 in activity buffer

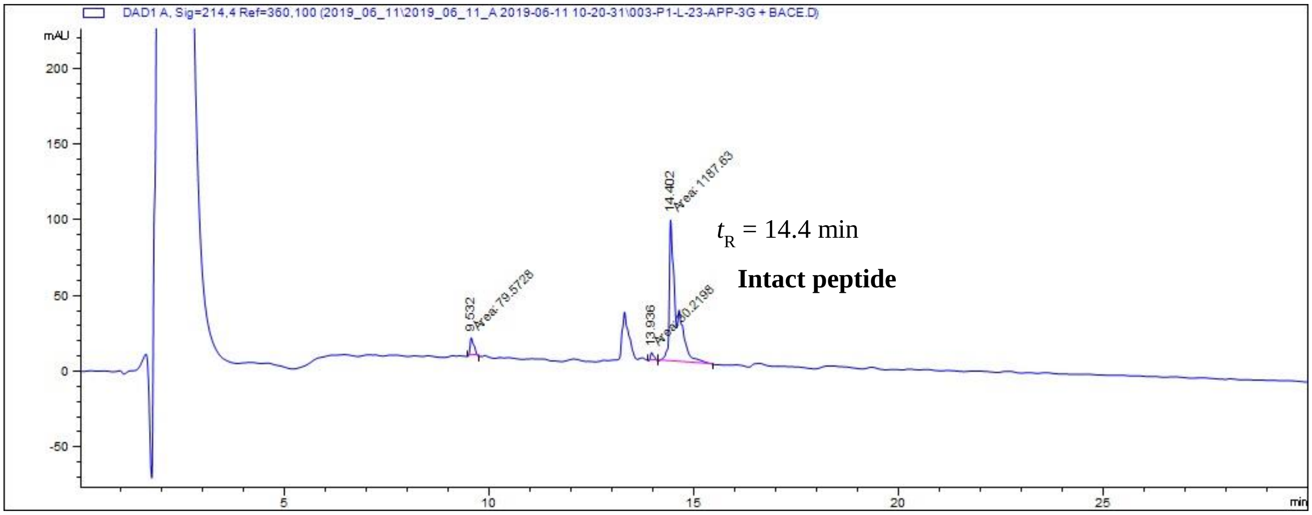

(m) APP$^{661\text{-}680}$(NL), **10** in activity buffer

IKTEEISEV**NL**~DAEFRHDSG

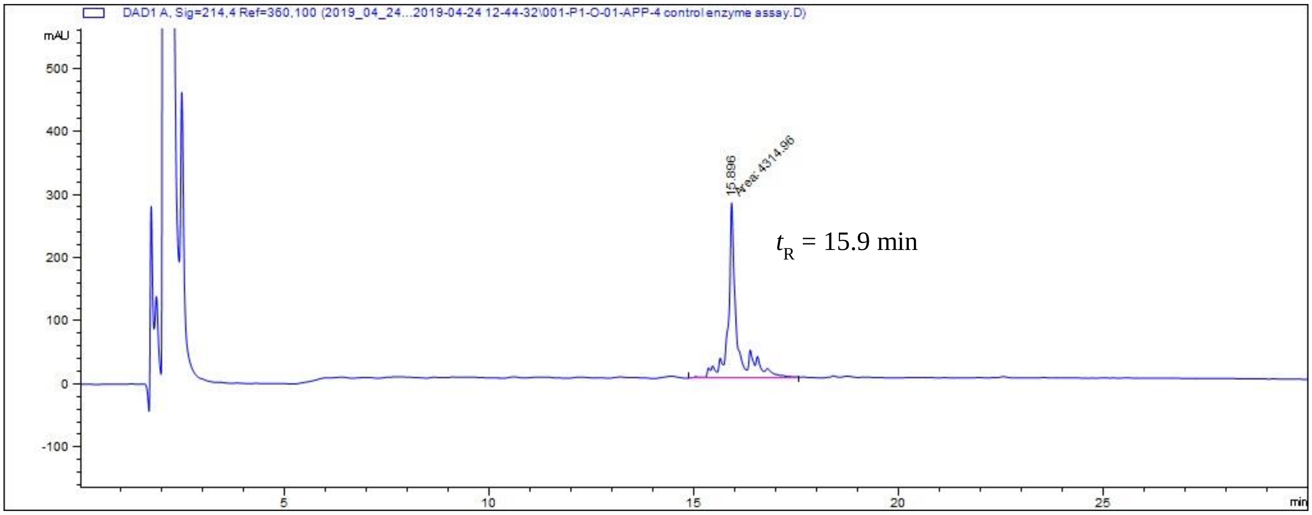

(n) APP$^{661\text{-}680}$(NL) (**10**) + BACE1 in activity buffer

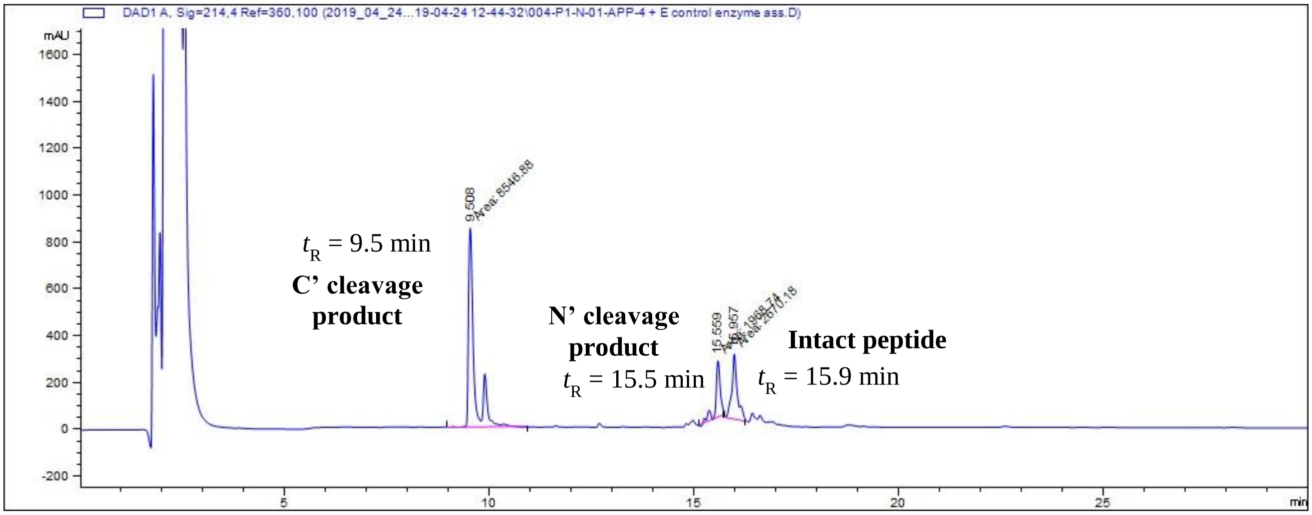

MALDI-TOF MS of BACE1 cleaved N’ fragment of peptide **10**

N’ fragment: IKTEEISEV**NL**

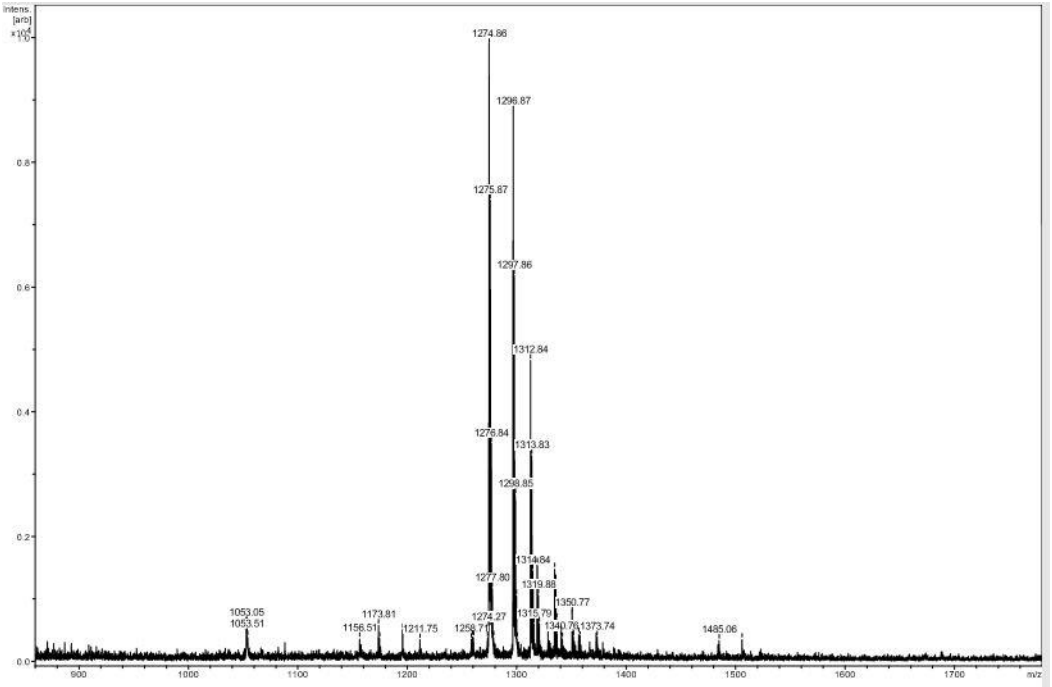

Expected $[M + H]^+$ = 1273.43 Da   Observed $[M + H]^+$ = 1274.86 Da

(o) APP$^{661\text{-}680}$(NL)-T*, **11** in activity buffer

IK**T***EEISEV**NL**~DAEFRHDSG

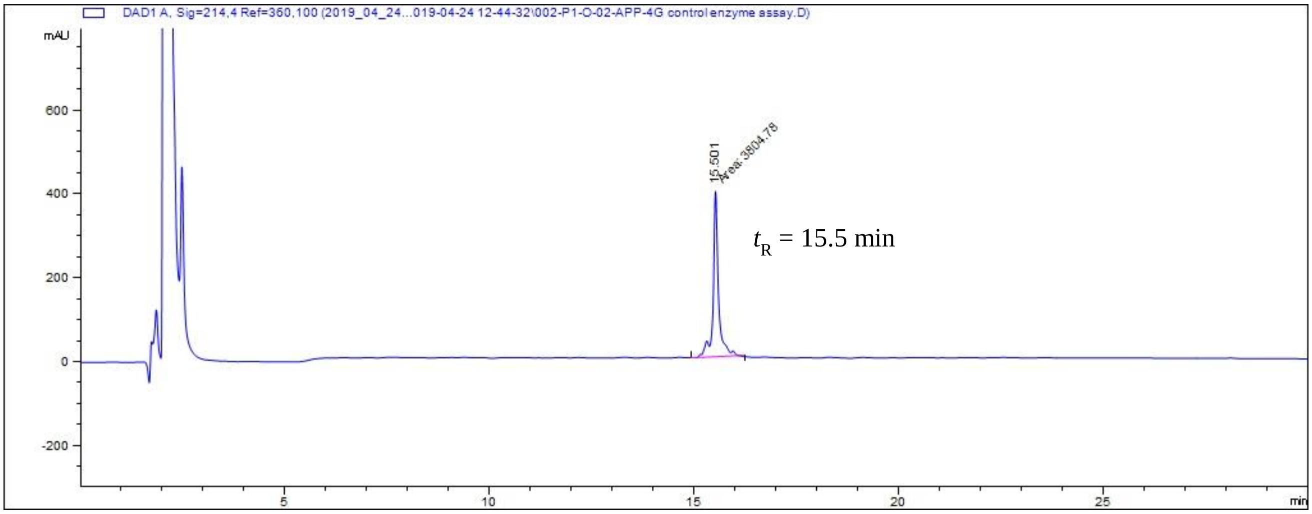

(p) APP$^{661\text{-}680}$(NL)-T* (**11**) + BACE1 in activity buffer

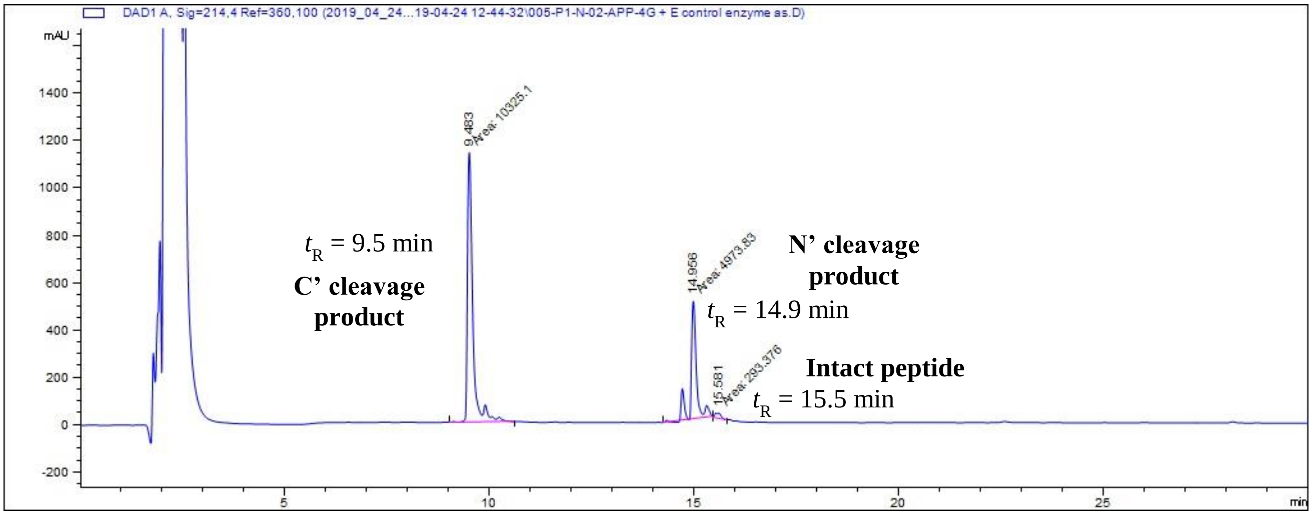

MALDI-TOF MS of BACE1 cleaved N' fragment of peptide **11**

N' fragment: IK**T***EEISEV**NL**

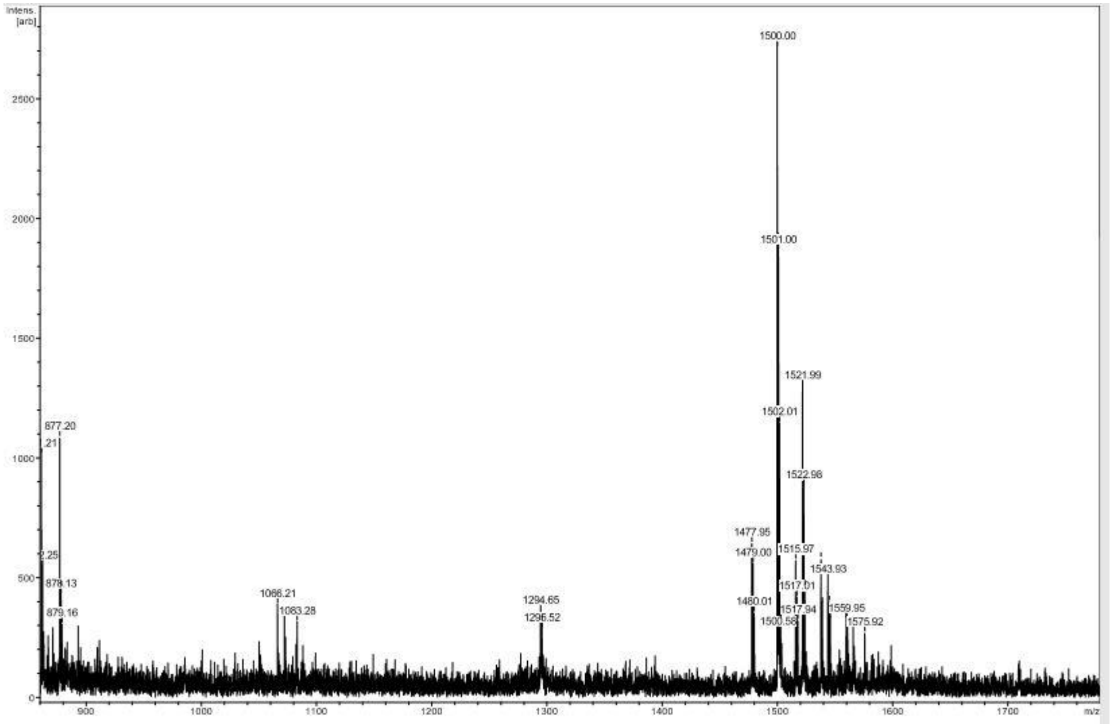

Expected [M + H]$^{+}$ = 1476.43 Da   Observed [M + Na]$^{+}$ = 1500.00 Da

RP-HPLC gradient on Vydac Denali C18 column (250 x 4.6 mm, 5 μm, 120Å):
Eluents were 0.1% TFA in water (A) and 0.1% TFA in acetonitrile (B). The elution gradient was 0-60%B in 30 minutes with a flow rate of 1 mL/min. Detection was at λ = 214 nm.

(q) APP$^{661\text{-}680}$-T*, S*, **9** in activity buffer

IK**T***EEI**S***EVKM~DAEFRHDSG

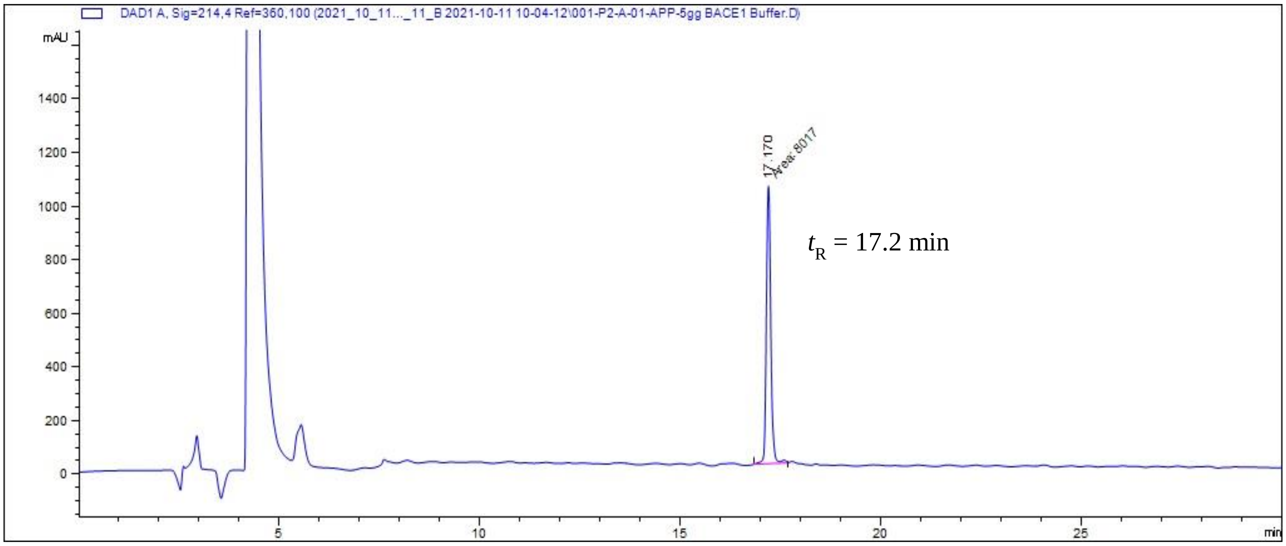

(r) APP$^{661\text{-}680}$-T*, S* (**9**) + BACE1 in activity buffer

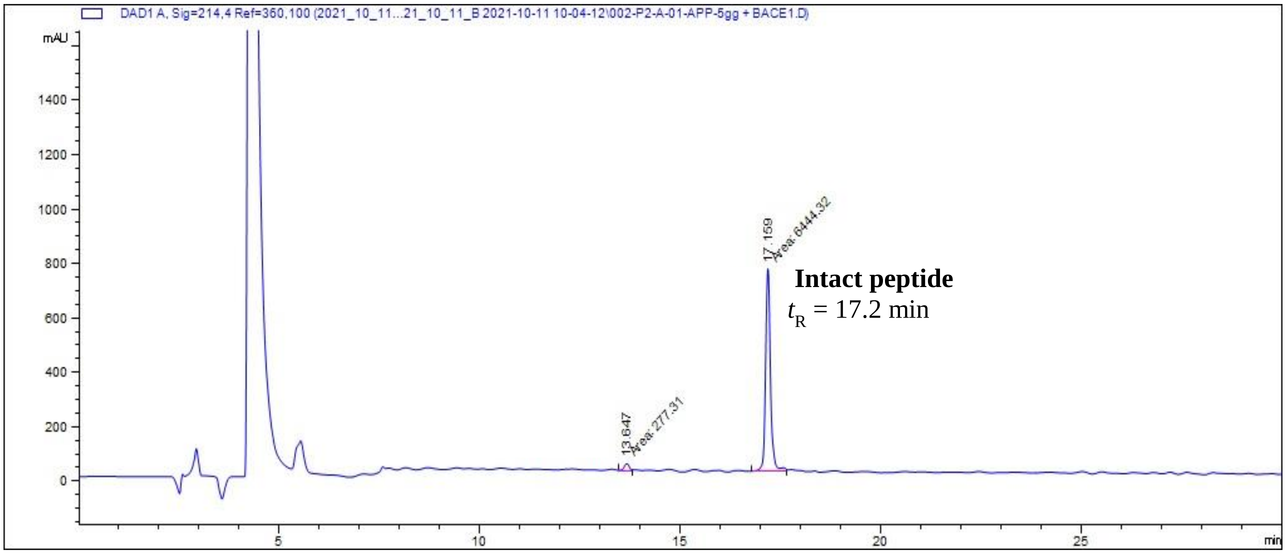

(s) APP$^{661\text{-}680}$(NL)-T*, S*, **12** in activity buffer

IK**T***EEI**S***EV**NL**~DAEFRHDSG

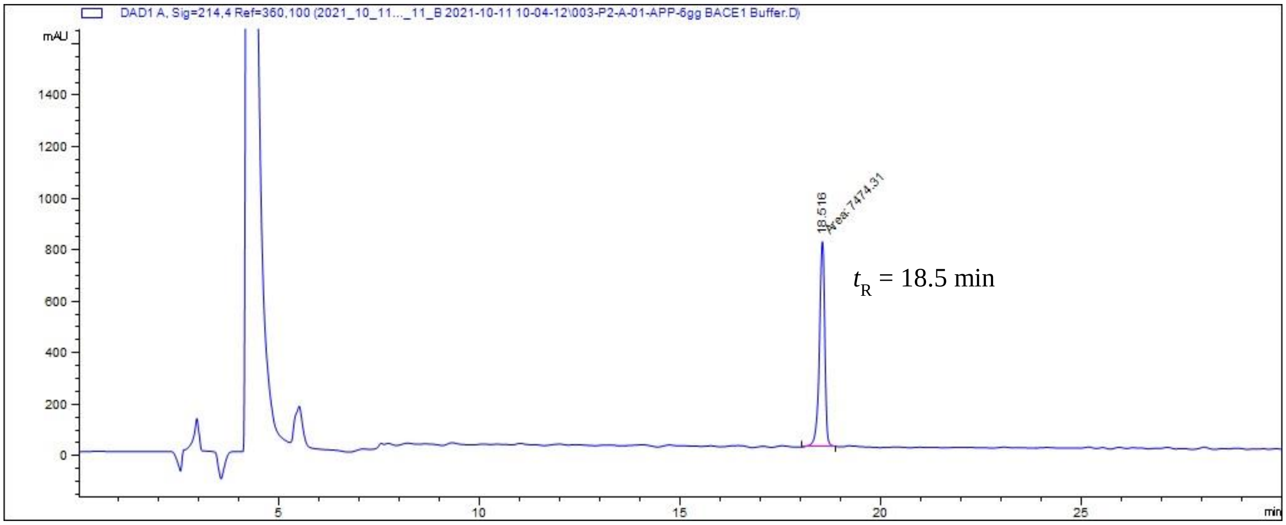

(t) APP$^{661\text{-}680}$(NL)-T*, S* (**12**) + BACE1 in activity buffer

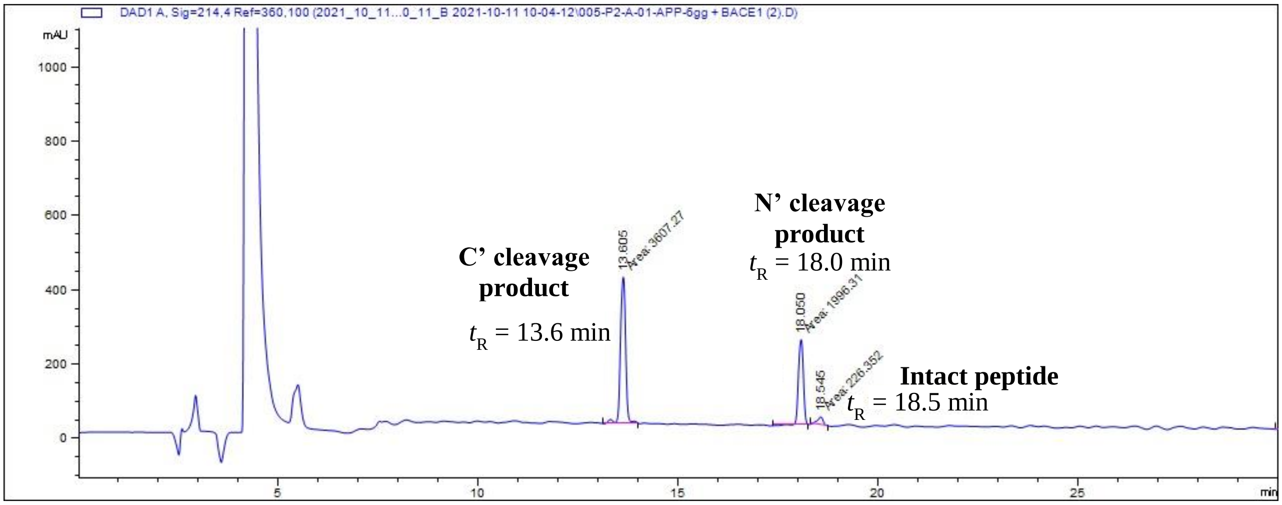

MALDI-TOF MS of BACE1 cleaved fragments of peptide **12**

N’ fragment: IK**T***EEI**S***EV**NL**

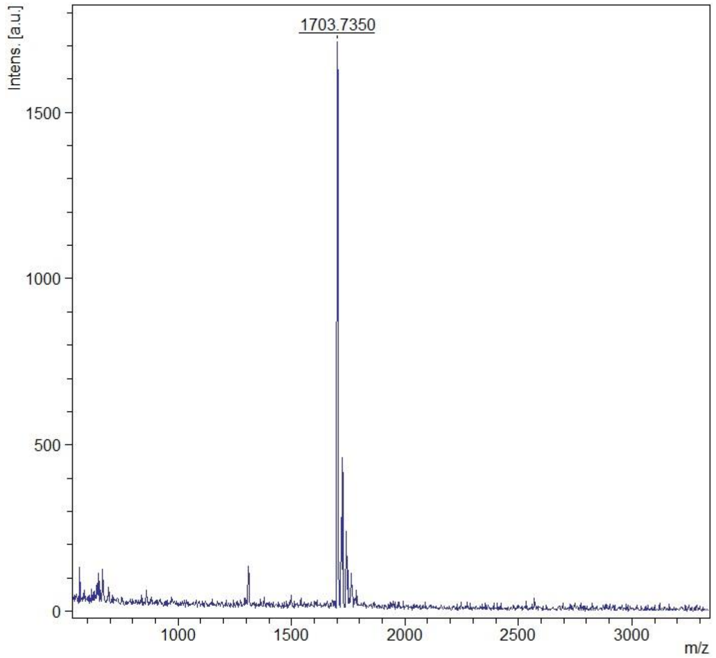

Expected $[M + H]^+$ = 1679.43 Da   Observed $[M + Na]^+$ = 1703.73 Da

C’ fragment: DAEFRHDSG

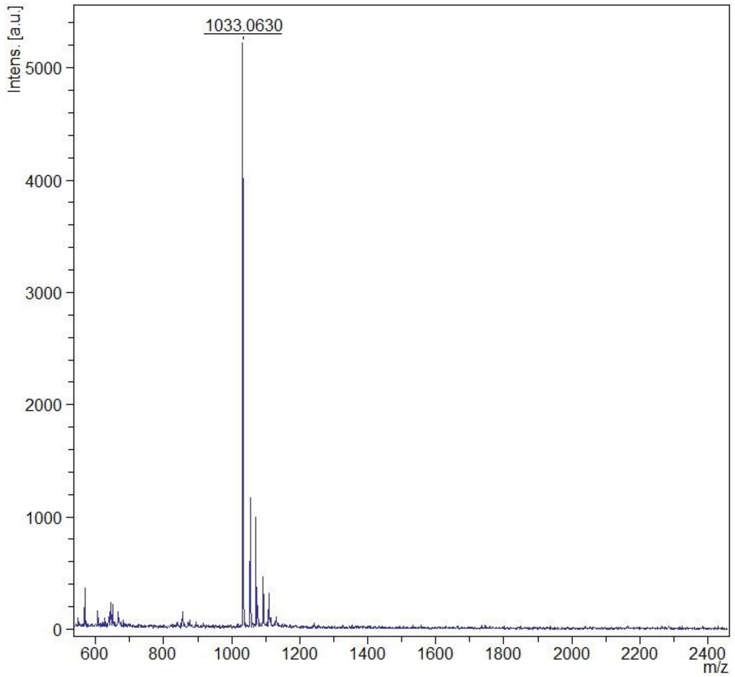

Expected $[M + H]^+$ = 1032.02 Da   Observed $[M + H]^+$ = 1033.06 Da

4. Aggregation kinetic ThT fluorescence curves of APP (glyco)peptides **3-16** coincubated with Aβ40

For the aggregation kinetic assay, APP (glyco)peptide stock solutions were added to the prepared Aβ40, phosphate buffer and ThT solution for final APP (glyco)peptide concentrations of 10 µM and 50 µM. 100 µL of prepared solution was distributed into each well and carried out in triplicate at 37 °C. ThT fluorescence was measured in 10 minute intervals.

Figure S1a. APP$^{665\text{-}680}$, **3** coincubated with Aβ40

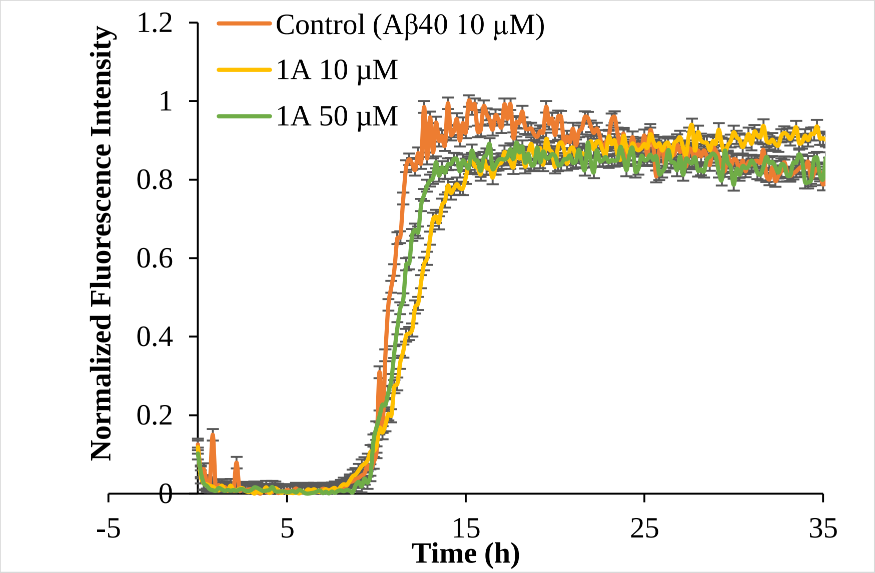

Figure S1b. APP$^{665\text{-}680}$-S*, **4** coincubated with Aβ40

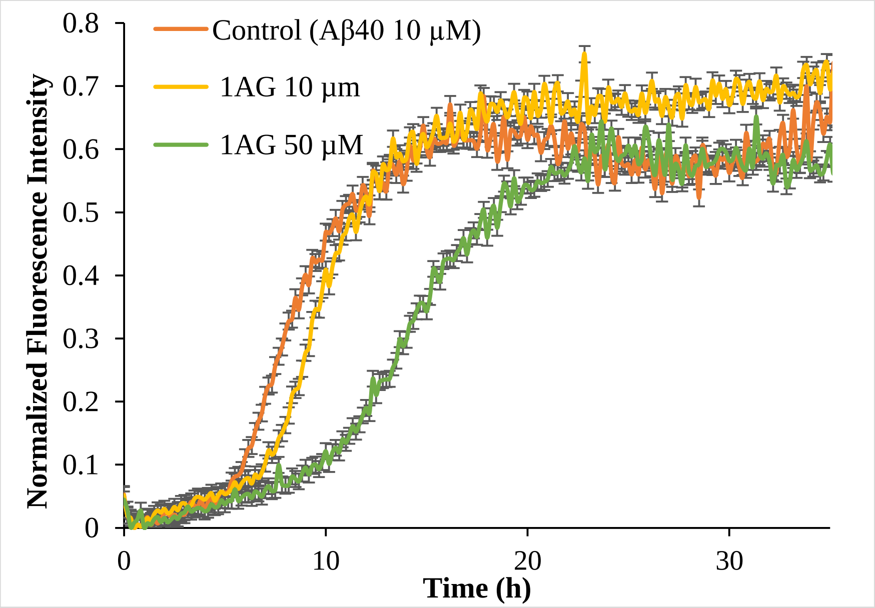

Figure S2a. APP$^{665-680}$(NL), **5** coincubated with Aβ40

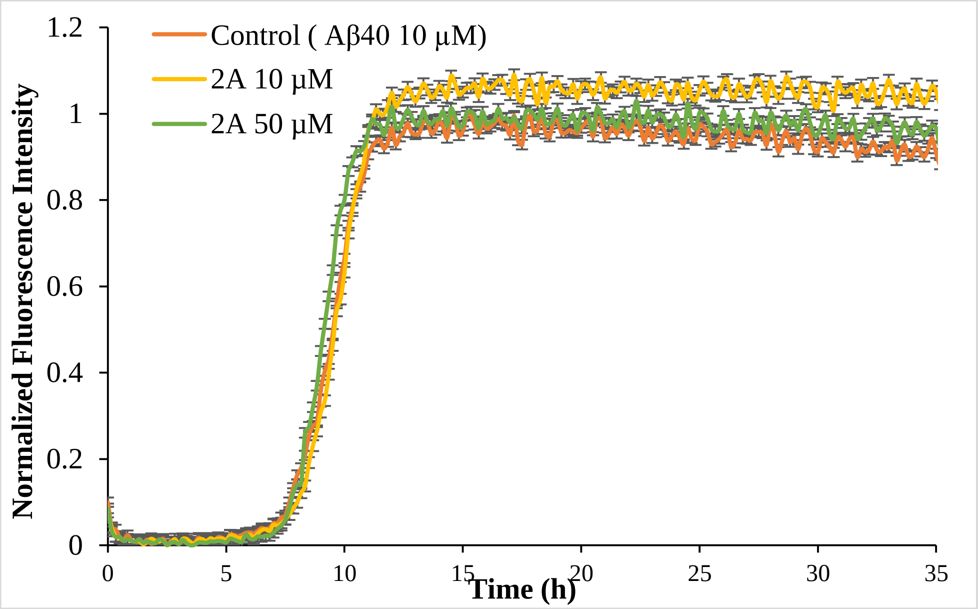

Figure S2b. APP$^{665-680}$(NL)-S*, **6** coincubated with Aβ40

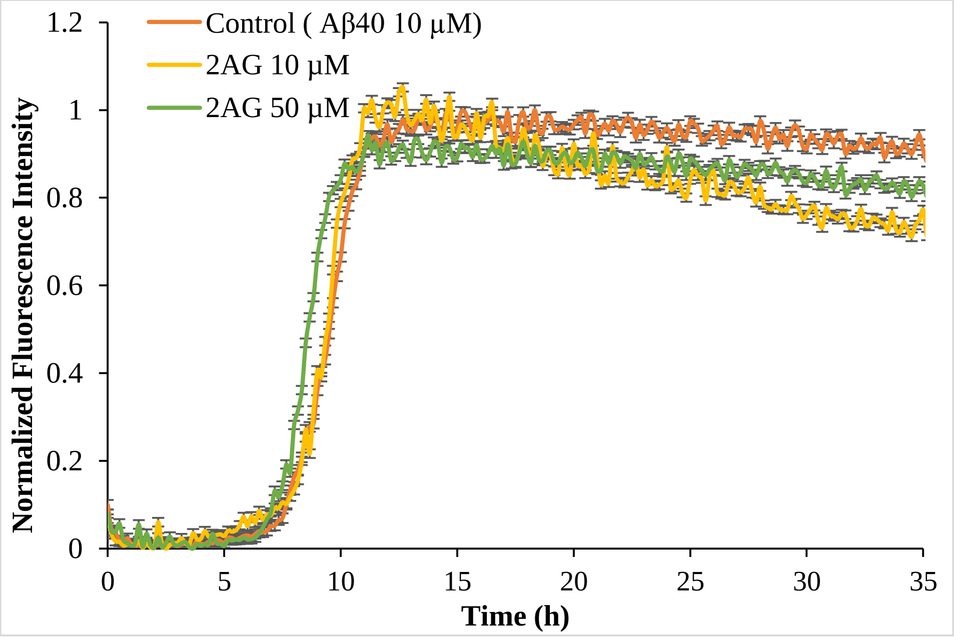

Figure S3a. APP$^{661-680}$, **7** coincubated with Aβ40

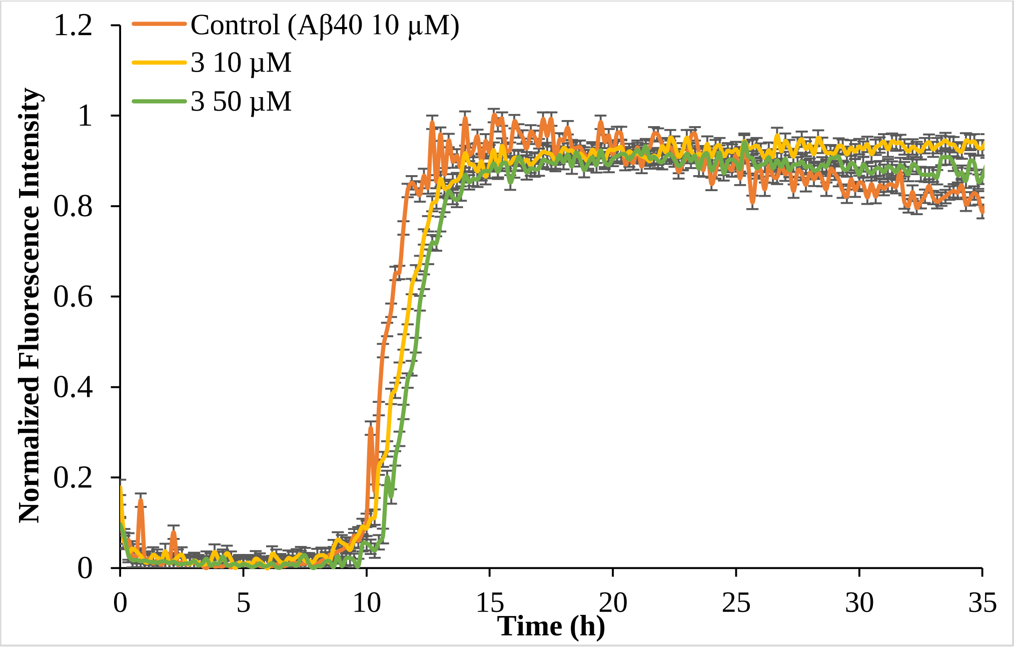

Figure S3b. APP$^{661-680}$-T*, **8** coincubated with Aβ40

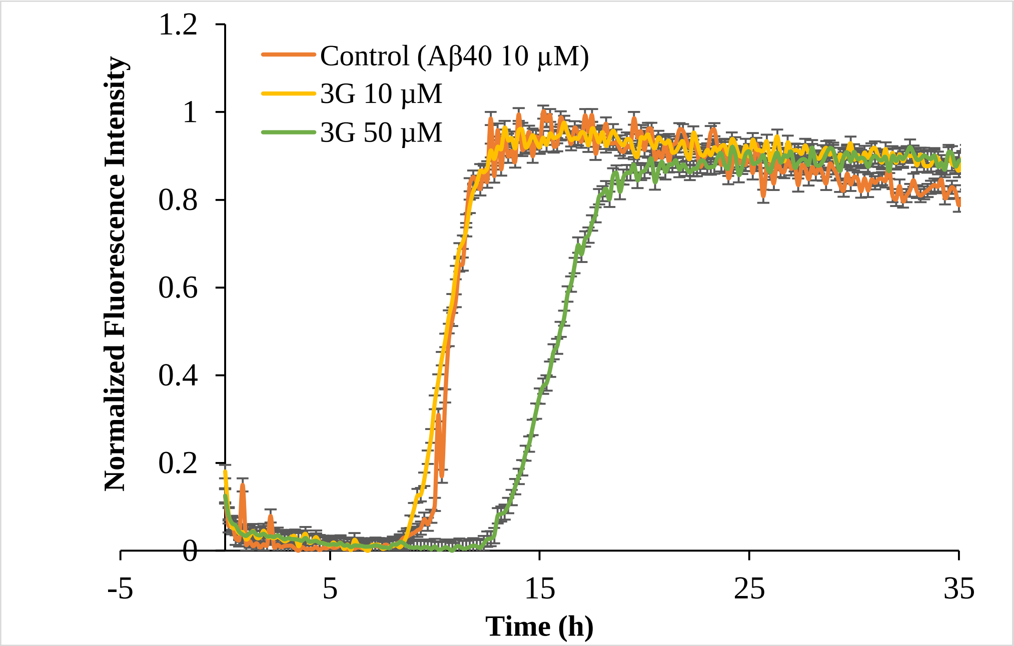

Figure S3c. APP$^{661\text{-}680}$-T*, S*, **9** coincubated with Aβ40

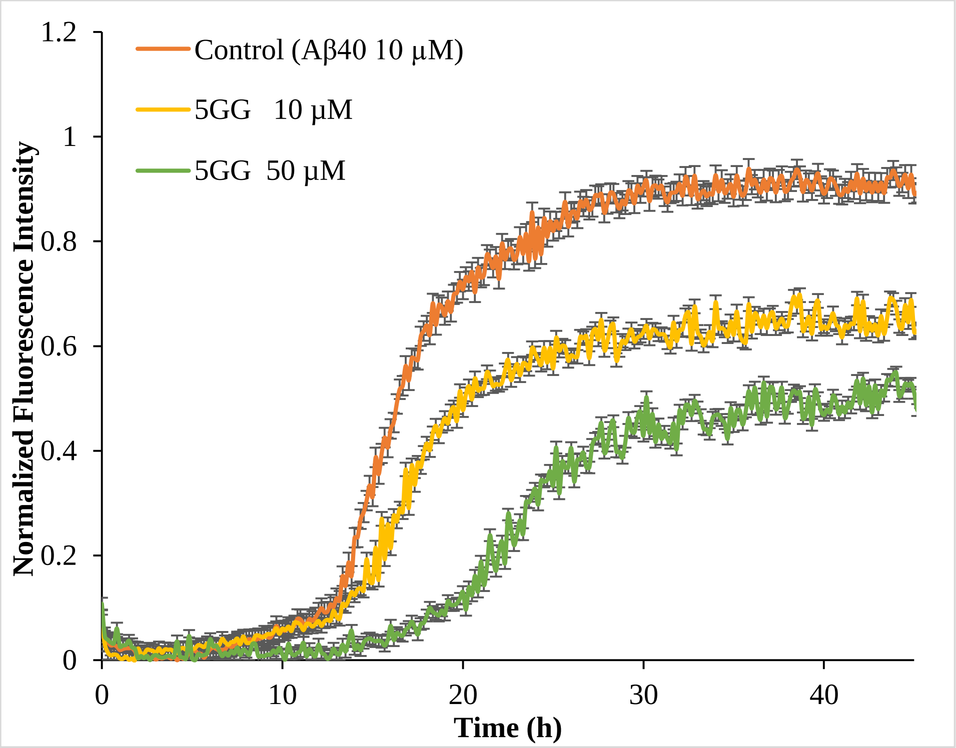

Figure S4a. APP$^{661\text{-}680}$(NL), **10** coincubated with Aβ40

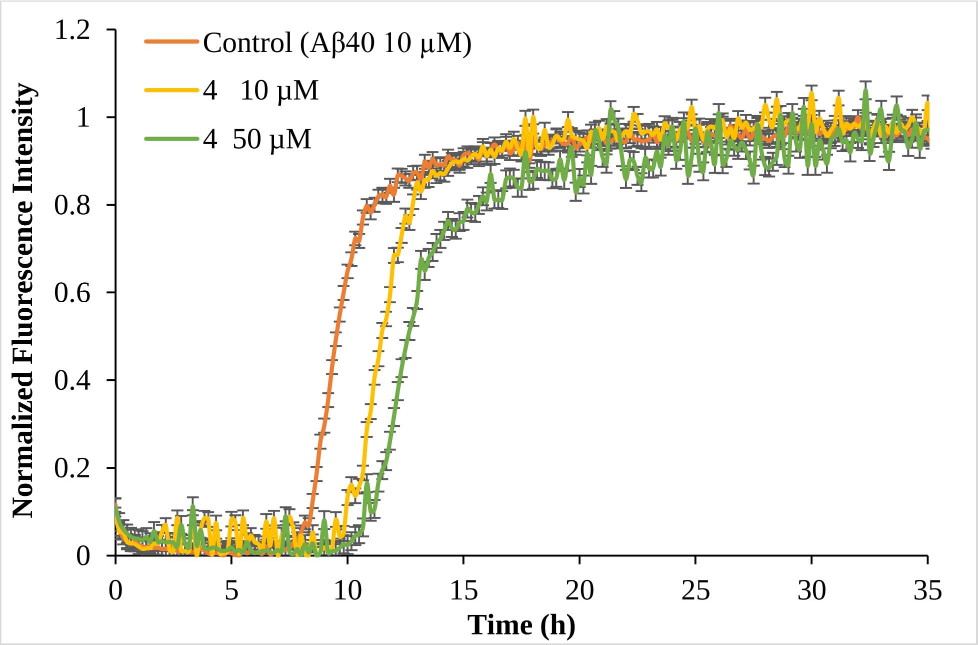

Figure S4b. APP$^{661-680}$(NL)-T*, **11** coincubated with Aβ40

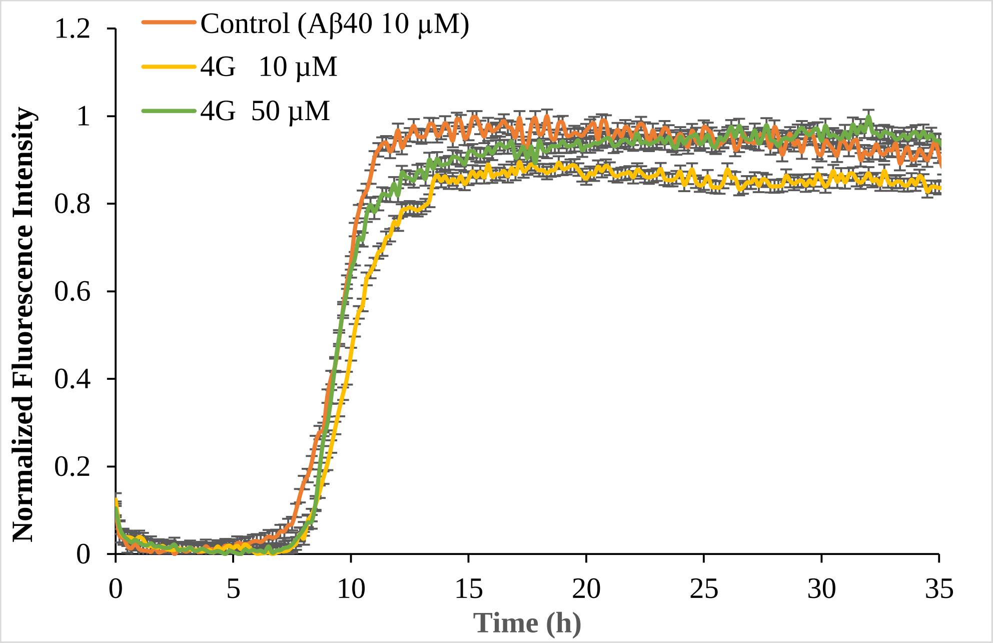

Figure S4c. APP$^{661-680}$(NL)-T*, S*, **12** coincubated with Aβ40

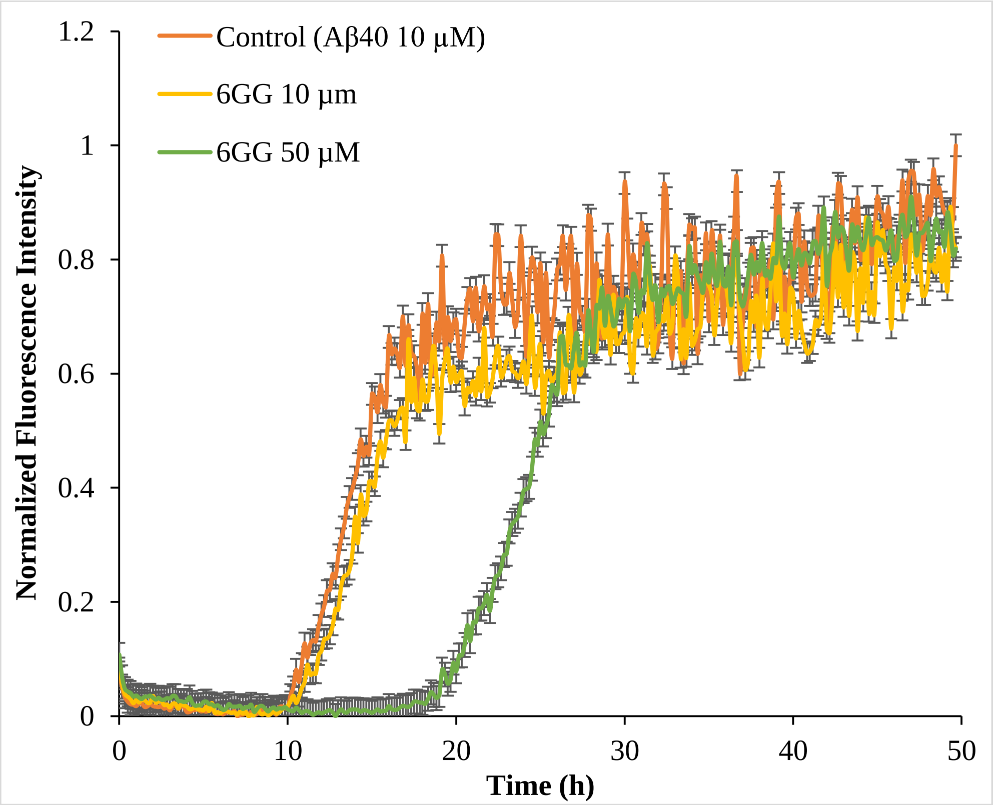

Figure S5a. APP$^{661\text{-}694}$, **13** coincubated with Aβ40

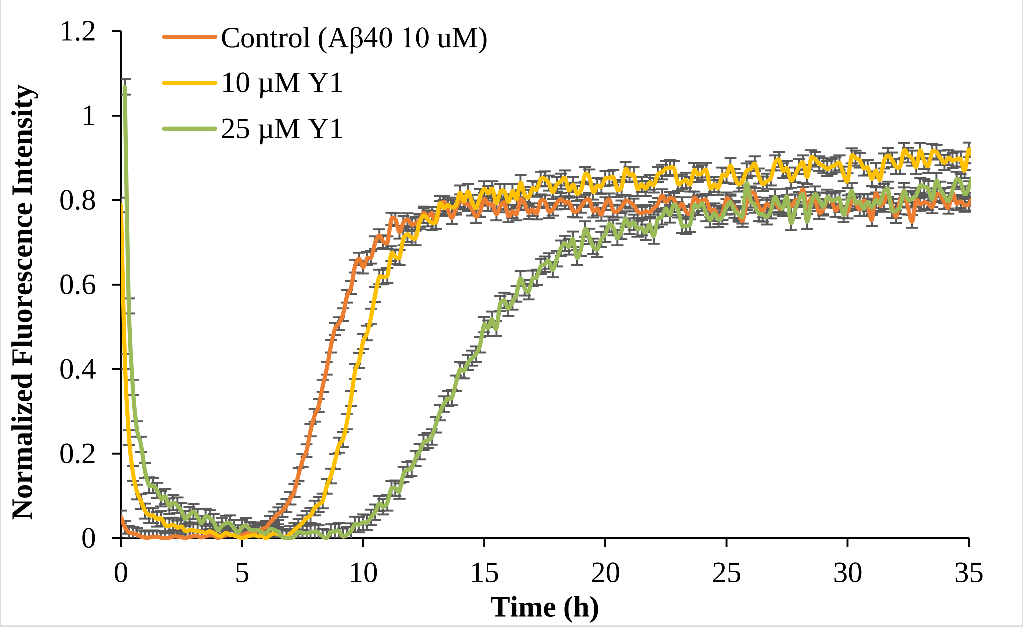

Figure S5b. APP$^{661\text{-}694}$, **13** coincubated with Aβ40 (saturated)

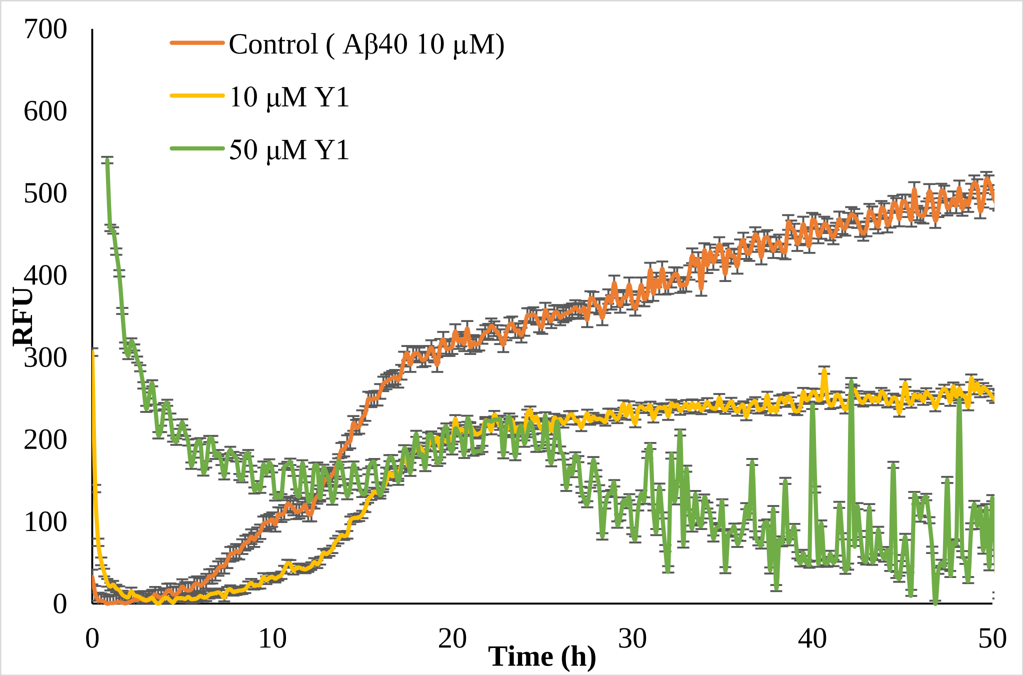

Figure S5c. $APP^{661\text{-}694}$-Y*, **14** coincubated with Aβ40

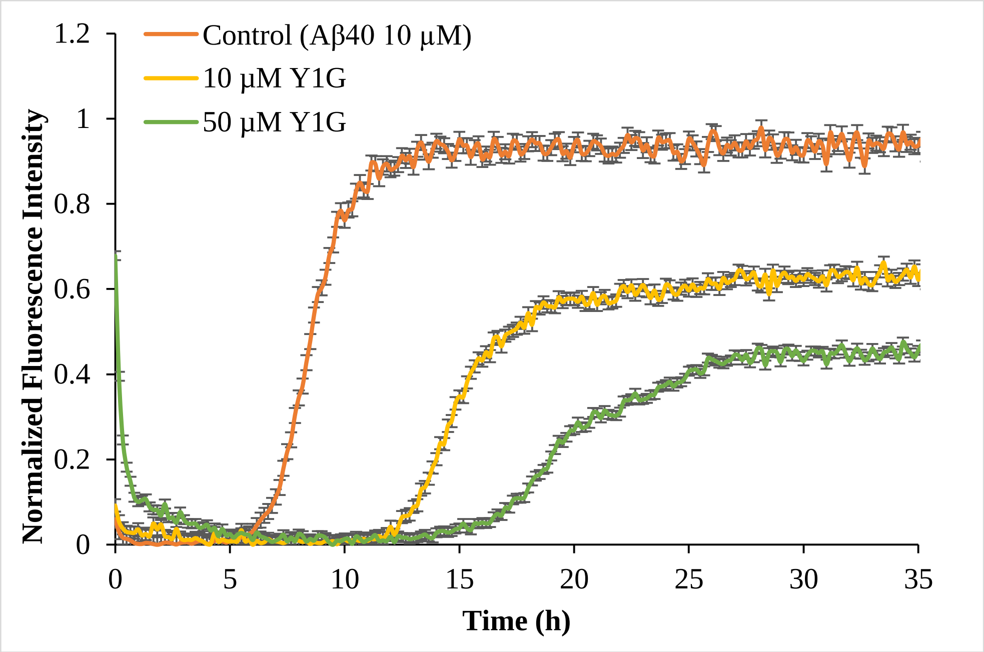

Figure S6a. $APP^{661\text{-}694}$(NL), **15** coincubated with Aβ40

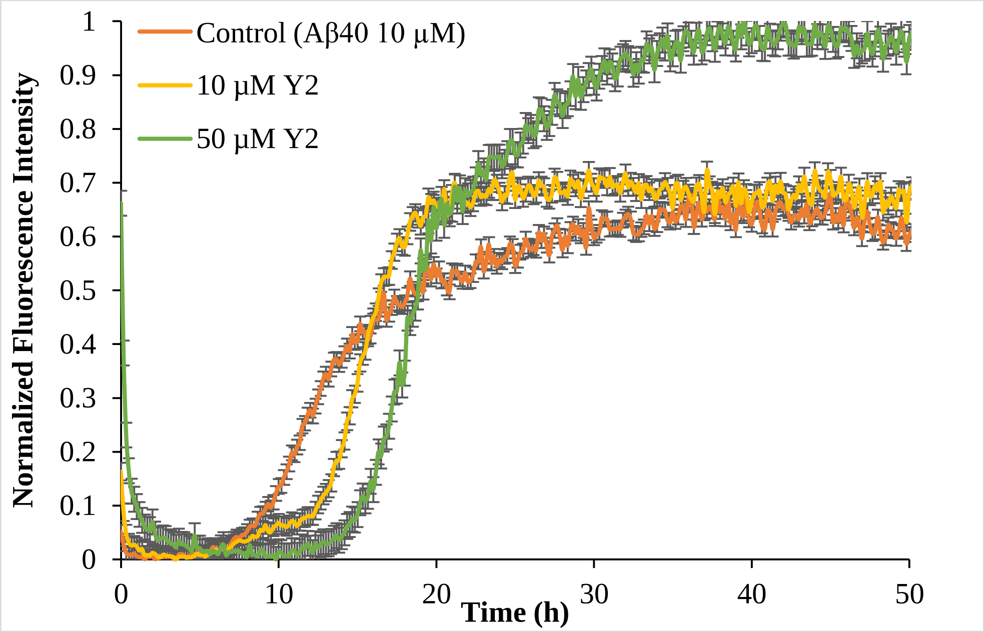

Figure S6b. APP$^{661\text{-}694}$(NL)-Y*, **16** coincubated with Aβ40

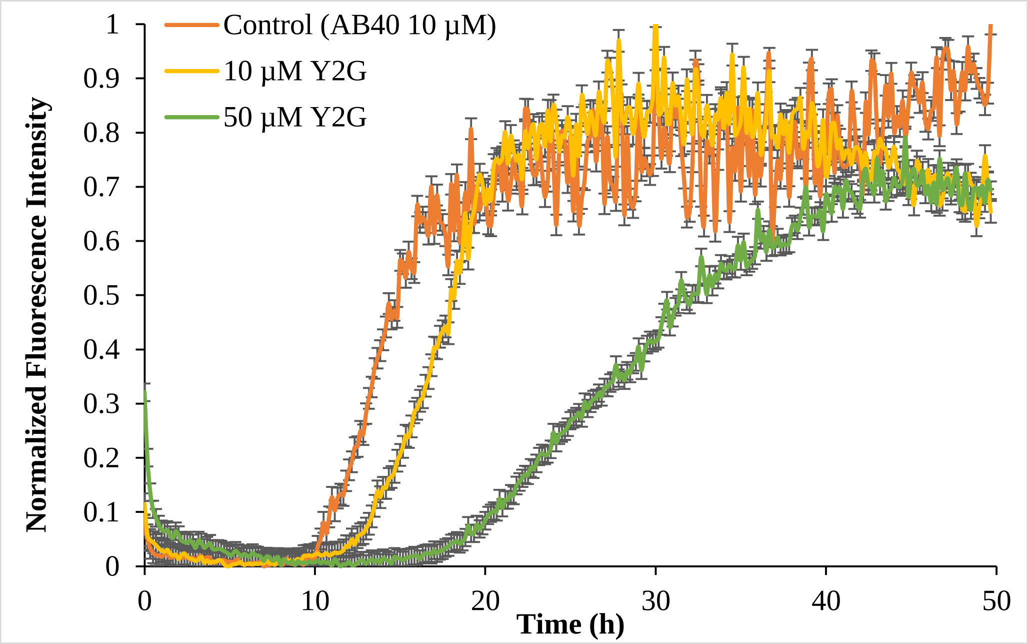

Supplement: Supplementary file 1 [file DataSheet1.PDF]
